# Mutant p53 protein accumulation is selectively targetable by proximity-inducing drugs

**DOI:** 10.1038/s41589-025-02051-7

**Published:** 2025-11-03

**Authors:** Ananthan Sadagopan, Maximilian Carson, Eriks J. Zamurs, Nicholas Garaffo, Heng-Jui Chang, Stuart L. Schreiber, Matthew Meyerson, William J. Gibson

**Affiliations:** 1 Broad Institute of MIT and Harvard, Cambridge, MA USA; 2 Department of Medical Oncology, Dana-Farber Cancer Institute, Boston, MA USA; 3 Division of Medical Sciences, Harvard Medical School, Boston, MA USA; 4 Department of Chemistry and Chemical Biology, Harvard University, Cambridge, MA USA; 5 Department of Medicine, Harvard Medical School, Boston, MA USA

**Keywords:** Cell death, Small molecules, Cancer therapy, Cell signalling

## Abstract

*TP53* mutant cancers are associated with approximately half of cancer deaths. The most common mechanism of p53 inactivation involves missense mutations. Such mutations in *TP53* result in a robust upregulation of the p53 protein. Here, we demonstrate an induced proximity approach to selectively kill *TP53* mutant cells. This approach uses the increased abundance of p53 protein in *TP53* mutant cancer cells to concentrate toxic molecules in these cells. We demonstrate this approach with a molecule that binds the Y220C mutant of p53 and concentrates a PLK1 inhibitor in cells harboring *TP53*^Y220C^ mutations. The resulting bifunctional molecule promotes formation of a p53^Y220C^–PLK1 ternary complex, mislocalizes PLK1, inhibits PLK1 activity, elicits selective G2/M arrest and induces apoptosis in *TP53*^Y220C^ cells while sparing wild-type *TP53* cells. These data exemplify a potentially generalizable framework for targeting *TP53* missense mutations by leveraging mutant p53 protein abundance to induce cell death, independent of p53’s transcriptional activity.

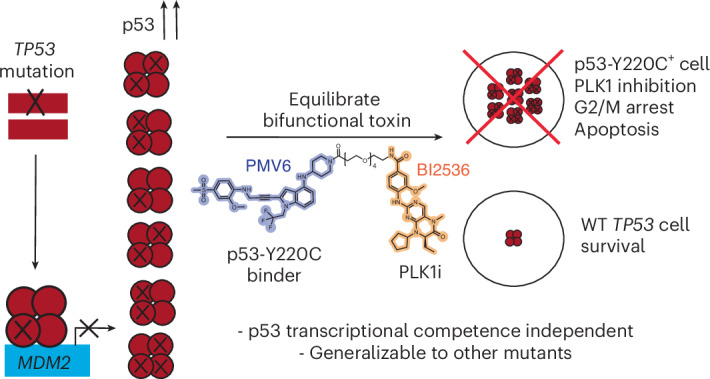

## Abstract (web summary)

Exploiting the high abundance of mutant p53 protein, a small molecule was developed that specifically binds to p53-Y220C mutants and delivers a mitosis blocker, killing *TP53*-mutant cancer cells while sparing healthy ones.

## Main

*TP53* is the most mutated gene in human cancer (36% according to the Pan-Cancer Analysis of Whole Genomes, 37% according to The Cancer Genome Atlas (TCGA) and 39% according to the American Association for Cancer Research GENIE project)^1–3^. *TP53* mutations have been shown to induce resistance to cytotoxic chemotherapy and are associated with significantly worse prognosis across nearly all cancers^4–6^. In total, *TP53*-mutant cancers are estimated to be associated with ~46% of cancer deaths (Supplementary Fig. 1; analysis across cBioPortal^7,8^). In the past, attempts have been made to therapeutically target *TP53*-mutant cancers in several ways^9^: delivery of wild-type (WT) *TP53* genes to cells with gene therapy or mRNA^10,11^, immunotherapy^12–14^ or small-molecule refolders of p53 protein^15,16^. Attempts to enact these strategies have invariably failed, and the activity of purported mutant-agnostic p53 refolders has consistently been shown to be off-target^17,18^. Furthermore, small-molecule refolding of p53 may not be possible for contact mutants of p53 specifically affecting residues that interact with the negatively charged phosphate backbone of DNA. New generalizable strategies are needed to specifically kill cancer cells harboring these mutations.

In contrast to mutations of most tumor suppressor genes, such as *APC*, *RB1* or *PTEN*, *TP53* mutations are typically missense mutations. These mutations act as dominant negative mutations that disallow DNA binding at consensus sequences and, therefore, poison the tetramer^19^. Because they act as dominant negatives, they function as a shorter evolutionary path to inactivating most p53 functions within the cells that harbor these mutations^20–22^. p53 typically acts as a central signal integrator for various cellular stress signals such as hypoxia, DNA damage or excessive oncogenic signaling^23,24^. When these mutations occur, the mechanisms that normally sense the above cellular stressors are typically intact and jointly work to activate p53 by increasing its abundance and promoting its nuclear translocation. Meanwhile the mutant p53 that accumulates is impotent to drive the transcription of its own destroyer, MDM2. The negative feedback loop that usually serves to tightly regulate p53 protein is broken and the half-life (*t*_1/2_) of p53 stretches from its typical ~5–20 min to several hours. Thus, p53 protein accumulates in cells with *TP53* missense mutations^25–29^.

Several recent examples have revealed the powerful effects of gain-of-function-induced proximity pharmacology such as transcriptional and epigenetic chemical inducers of proximity, velcrins, regulated induced proximity-targeting chimeras (RIPTACs) or cancer mutation-specific effectors of targeted protein degradation^30–33^. Application of gain-of-function approaches might provide a generalizable method to target deadly cancers associated with mutant *TP53*. Here, we develop gain-of-function small molecules that target the abundance of p53 protein, a characteristic of cancers harboring *TP53* missense mutants, to selectively kill cancer cells with high levels of mutant p53.

## Results

### *TP53*-mutant cells lack synthetic lethal dependencies

**Fig. 1:**
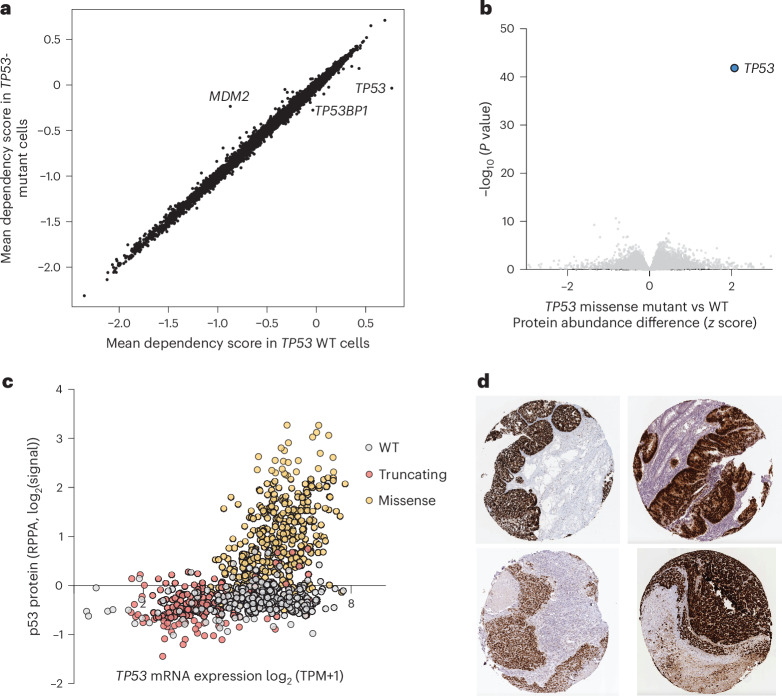
p53 protein abundance is the only genetic or proteomic distinction between *TP53*-mutant and WT cancer cells. **a**, Mean CRISPR dependency scores for genes across DepMap in *TP53* WT (*x* axis) versus *TP53*-mutant cells (*y* axis). There are no genes with (mean DepScore)_Mut_ < −0.5 and Δ(mean DepScore)_Mut-WT_ < −0.2. **b**, Proteome-wide volcano plot of proteins that are enriched or depleted in *TP53*-mutant cells versus *TP53* WT cells across DepMap (difference in mean *z*-scored quantitative proteomics abundance; *t*-test). **c**, *TP53* mRNA expression (log_2_(transcripts per million + 1)) versus p53 protein abundance (log_2_(signal) from RPPA) across DepMap. Cell lines are colored by *TP53* mutation status (WT, gray; truncating, red; missense, yellow). **d**, Representative immunohistochemistry of p53 in the Human Protein Atlas^66^ from resected tumors.

**Extended Data Fig. 1:**
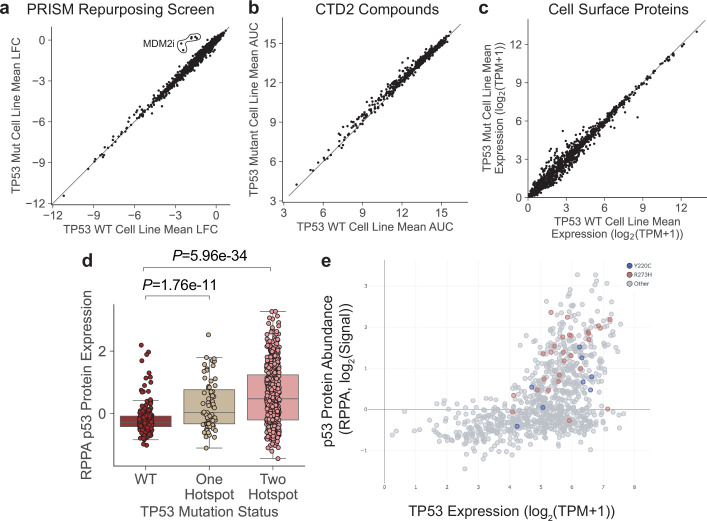
No small molecules specifically kill p53 mutant cancer cells or cell surface receptors upregulated on TP53 mutant cells. **a**, Mean log_2_(fold-change) for compounds in the PRISM repurposing library (23Q2) on the viability of *TP53* mutant (y-axis) and *TP53* wild-type cell lines (x-axis) across DepMap. MDM2 inhibitors are boxed. There are no molecules >2-fold more toxic on average to p53^Mut^ vs. p53^WT^ cells in the PRISM repurposing library. **b**, Mean area under the curve (AUC) for compounds in the CTD2 library on the viability of *TP53* mutant (y-axis) and *TP53* wild-type cell lines (x-axis) across DepMap. There are no molecules with Δ(Mean AUC)_Mut-WT_ < −0.5 in CTD2 screen. **c**, Mean mRNA expression (log_2_(TPM + 1)) for genes encoding cell surface proteins for *TP53* mutant (y-axis) and *TP53* wild-type cell lines across DepMap. **d**, p53 protein abundance (log_2_(RPPA Signal)) versus TP53 hotspot mutation status (WT, monoallelic, biallelic) across DepMap. **e**, p53 protein abundance (log_2_(RPPA Signal)) versus *TP53* hotspot mutation status for select hotspot mutations (R273H, Y220C) across DepMap.

Large-scale functional genomic studies have recently been undertaken to systematically determine the genetic vulnerabilities of nearly 1,000 cancer cell lines^34,35^. We sought to test the hypothesis that there exist synthetic lethal interactions with *TP53*-mutant cancer cells. We performed a genome-wide analysis of CRISPR dependencies in cells with *TP53* mutations versus WT cell lines. Unfortunately, there is no genetic dependency that is enriched in cancer cells with *TP53* loss-of-function mutations (Fig. 1a), consistent with a prior report^22^, nor any profiled small molecules selectively killing *TP53*-mutant cells (Extended Data Fig. 1a,b). One might instead hope that a subset of proteins are enriched in cells with *TP53* mutations, particularly cell surface proteins that might be amenable to targeting with a number of effector modalities such as chimeric antigen receptor T cells or antibody–drug conjugates. Unfortunately, no such cell-surface proteins are enriched in *TP53*-mutant cancers (Fig. 1b and Extended Data Fig. 1c).

### *TP53*-mutant cells strongly upregulate p53 protein

Quantitative proteomics of the Cancer Cell Line Encyclopedia (CCLE)^36^ shows that the only protein whose abundance is significantly increased in *TP53*-mutant cancers is p53 itself (Fig. 1b; Δ(*z*-scored abundance)_Mut-WT_ > 2, *q* < 0.05). p53 protein is expressed at a low level in *TP53* WT cancer cells but is elevated in cells with *TP53* missense mutations (Fig. 1c; >2-fold increase in mean reverse-phase protein array (RPPA) signal), for both monoallelic and biallelic mutations, as well as structural (for example, Y220C) and contact (for example, R273H) mutants (Extended Data Fig. 1d,e). Correspondingly, immunohistochemistry of p53 in tumor tissues shows abundant staining in cancer cells but not adjacent normal tissue (Fig. 1d). Taken together, these data demonstrate that the only genetic dependency or substantial proteomic difference between *TP53*-mutant cells and WT cells, detected to date, is the overabundance of p53 protein in *TP53*-mutant cells.

The increased abundance of the p53 protein in cancer cells with *TP53* missense mutations provides a unique therapeutic opportunity: a p53 concentration-dependent toxin could selectively kill *TP53*-mutant cancer cells in a way that is generalizable to multiple *TP53* missense mutations.

### Bifunctional molecules kill cells with p53 overabundance

**Extended Data Fig. 2:**
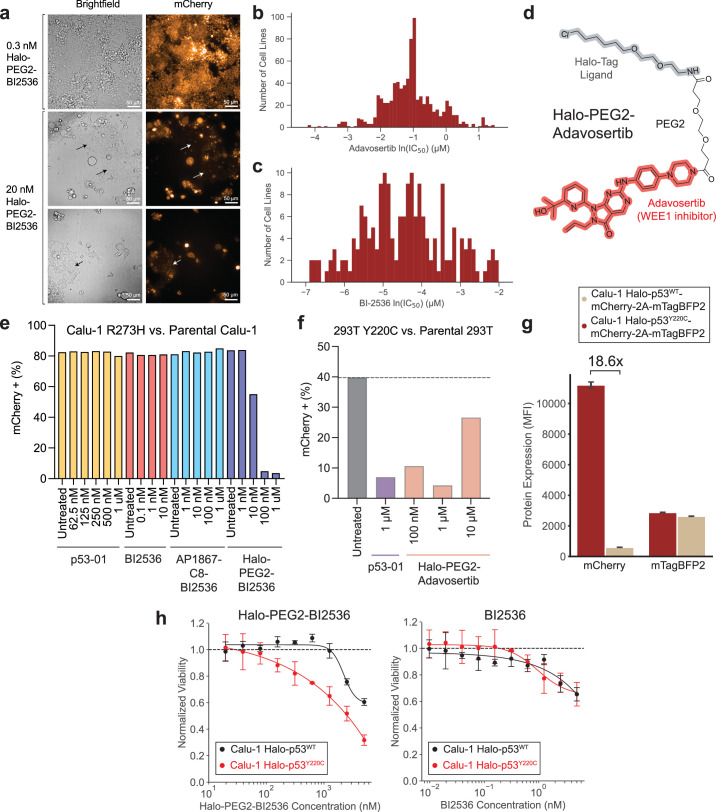
Halo bifunctionals selectively kill cells overexpressing Halo-p53. **a**, Brightfield and mCherry imaging of Halo-p53^R273H^(FL)-mCherry 293 T cells treated with Halo-PEG2-BI2536 (top row: 0.3 nM, bottom two rows: 20 nM) for 8 days. Scale bar is shown (50 μm). Experiment performed in duplicate; representative replicate shown. **b**, ln(IC_50_) for adavosertib in DepMap (PRISM OncRef), **c**, ln(IC_50_) for BI-2536 in DepMap (GDSC1). **d**, Structure of Halo-PEG2-adavosertib. **e**, Competition of Halo-p53^R273H^(FL)-mCherry vs. parental Calu-1 cells with p53-01, BI2536, AP1867-C8-BI2536, or Halo-PEG2-BI2536 for 7 days; analyzed for mCherry expression by flow cytometry; one replicate was performed. **f**, Competition of Halo-p53^Y220C^ΔTAD-mCherry vs. parental 293 T cells with p53-01 or Halo-PEG2-adavosertib for 8 days; analyzed for mCherry expression by flow cytometry. Experiment performed in duplicate; representative replicate shown. **g**, Calu-1 cell lines stably expressing Halo-p53^WT^(FL)-mCherry-2A-mTagBFP2-V5 or Halo-p53^Y220C^(FL)-mCherry-2A-mTagBFP2-V5 were established. mTagBFP2 and mCherry expression was assessed by FACS, and mean fluorescence intensity is plotted. N = 3 measurements/condition. **h**, Cell viability of Calu-1 cell lines established in panel **g** treated with Halo-PEG2-BI2536 (left) or BI-2536 (right) measured by cell-titer-glo after 4 days. N = 6 technical replicates/condition. Source data

We, therefore, hypothesized that, if the abundance of p53 protein could be translated into a proportionate cell death signal, cells with *TP53* missense mutations could be selectively killed. We hypothesized that bifunctional small molecules consisting of a p53 binder and a small-molecule toxin may be able to concentrate the toxin in cells overexpressing mutant *TP53* (ref. ^33^). Unfortunately, no high-affinity ligands for WT p53 exist. In the absence of such ligands, we used the Halo tag as a surrogate for a small-molecule binder of p53. Mutant p53-R273H was stably expressed in 293T cells fused to a Halo tag and mCherry for visualization. The fusion protein showed nuclear expression similar to native p53 (Extended Data Fig. 2a).

**Fig. 2:**
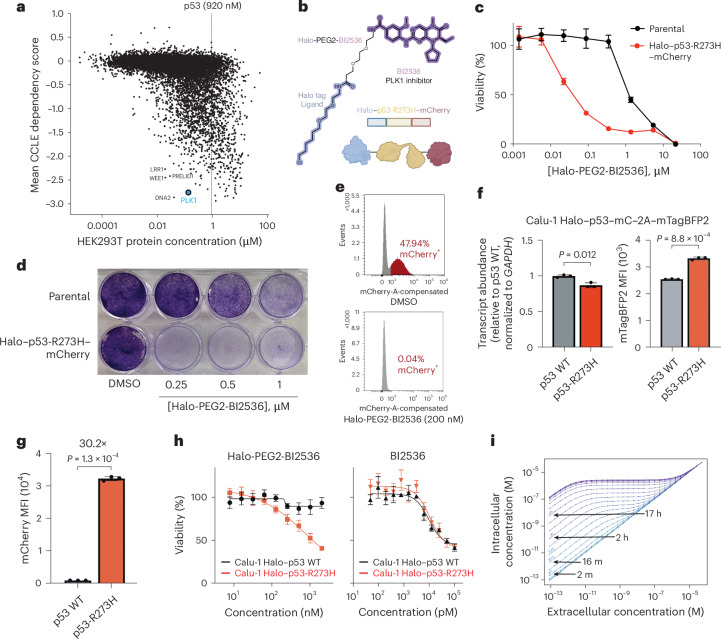
Bifunctional molecules liganding p53 fusion proteins selectively inhibit proliferation of *TP53*-mutant cells. **a**, Protein concentration in HEK293T cells profiled in OpenCell versus mean CRISPR dependency score across DepMap for all genes and protein products. **b**, Structure of small molecule (Halo-PEG2-BI2536) and fusion protein (Halo–p53-R273H (FL)–mCherry) constructs used in experiments. **c**, Cell viability of 293T cells stably expressing Halo–p53-R273H (FL)–mCherry versus parental 293T cells treated with Halo-PEG2-BI2536, measured by CTG after 5 days (*n* = 8 technical replicates per condition). **d**, Crystal violet staining of 293T cells stably expressing Halo–p53-R273H (FL)–mCherry versus parental 293T cells treated with Halo-PEG2-BI2536, analyzed after 4 days. The experiment was performed in triplicate; a representative replicate is shown. **e**, Competition experiment in which a 1:1 mixture of Halo–p53-R273H (FL)–mCherry and parental 293T cells were treated with Halo-PEG2-BI2536 (200 nM) for 7 days and analyzed for mCherry expression by FACS. The experiment was performed in triplicate; a representative replicate is shown. **f**, Calu-1 cell lines stably expressing Halo–p53 WT (FL)–mCherry–2A–mTagBFP2-V5 and Halo–p53-R273H (FL)–mCherry–2A–mTagBFP2-V5 were established and analyzed by RT–qPCR for expression of the transgene. Transcript abundance is normalized to *GAPDH* expression using the 2^−∆∆*Ct*^ method and is plotted relative to Calu-1 cells stably expressing Halo–p53 WT (FL)–mCherry–2A–mTagBFP2-V5. mTagBFP2 expression in these cell lines was also assessed by FACS and the MFI is plotted. **g**, mCherry expression in Calu-1 cell lines established in **f** assessed by FACS; the MFI is plotted. **h**, Cell viability of Calu-1 cell lines established in **f** treated with Halo-PEG2-BI2536 (left) or BI2536 (right) measured by CTG after 4 days (*n* = 6 technical replicates per condition for Halo-PEG2-BI2536 and *n* = 4 technical replicates per condition for BI2536). **i**, Modeling of final intracellular concentration at steady state for a freely diffusing bifunctional compound binding to p53 (Methods) based on p53 protein *t*_1/2_ and initial extracellular concentration (held constant); purple indicates a longer *t*_1/2_. Source data

To leverage the abundance of p53 protein for therapeutic purposes, a bifunctional molecule would ideally be designed such that its binding partner is less abundant than overexpressed mutant p53 protein and highly essential for cell proliferation. To determine which cytotoxin to append to a potential bifunctional molecule, we compared CRISPR gene essentiality scores across Dependency Map (DepMap)^37^ and absolute protein abundance profiled in OpenCell^38^ (Fig. 2a). We identified five targets with high essentiality and low abundance: DNA2, WEE1, LRR1, PRELID1 and PLK1 (Fig. 2a and Methods). Of these, WEE1 and PLK1 have ligands previously used to synthesize bifunctional small molecules for targeted protein degradation^39–41^. We functionalized WEE1 inhibitor adavosertib^42^ (half-maximal inhibitory concentration (IC_50_) = 5.2 nM for cell-free WEE1 inhibition) and an analog of PLK1 inhibitor BI2536 (ref. ^43^) (IC_50_ = 0.83 nM for cell-free PLK1 inhibition), both of which display potent antiproliferative effects across DepMap (adavosertib median IC_50_: 348 nM, BI2536 median IC_50_: 11.8 nM; Extended Data Fig. 2b,c), with a Halo tag ligand and PEG2 linker, yielding the bifunctional small molecules Halo-PEG2-adavosertib (**1**) and Halo-PEG2-BI2536 (**2**) (Fig. 2b and Extended Data Fig. 2d).

Halo-PEG2-BI2536 inhibited proliferation of 293T cells stably expressing Halo–p53-R273H (full length (FL))–mCherry at doses between 20 nM and 500 nM, with little impact on the proliferation of parental 293T cells (Fig. 2c,d; p53-R273H 293T IC_50_: 23 nM, parental 293T IC_50_: 1,143 nM). When competing Halo–p53-R273H (FL)–mCherry 293T cells with parental 293T cells in the presence of Halo-PEG2-BI2536 (200 nM), we observed a 1,200-fold decline in the population of mCherry^+^ cells after 1 week (0.04% versus 47.94% for DMSO control; Fig. 2e). These results were reproducible in a competition assay between the parental *TP53*-null (*TP53* homozygous deletion) Calu-1 cell line and Calu-1 cells stably expressing Halo–p53-R273H (FL)–mCherry (Extended Data Fig. 2e; half-maximal effective concentration (EC_50_) = 13 nM). Furthermore, treatment of Halo–p53-R273H (FL)–mCherry 293 T cells with low doses of Halo-PEG2-BI2536 resulted in selection for low expressors of the fusion protein (Extended Data Fig. 2a). Halo-PEG2-adavosertib also selectively inhibited the proliferation of Halo–p53-R273H (FL)–mCherry 293T cells in the competition assay at doses between 100 nM and 1 μM (Extended Data Fig. 2f; EC_50_ = 41 nM).

To assess the therapeutic window obtained by this strategy, we established Calu-1 cell lines stably expressing Halo–p53-R273H (FL)–mCherry and mTagBFP2 from the same transcript using 2A peptides. We established versions of this cell line for *TP53* WT and *TP53*^R273H^. We confirmed that these cell lines displayed approximately equal expression of the transgene by reverse transcription (RT)–qPCR for the transcript and fluorescence-activated cell sorting (FACS) for mTagBFP2 (Fig. 2f; mean [*TP53* WT mRNA]/[*TP53*^R273H^ mRNA] = 1.14; mean mTagBFP2 mean fluorescence intensity (MFI) p53 WT/p53-R237H = 0.77). We then compared expression of Halo–p53-R273H (FL)–mCherry between the cell lines using FACS for mCherry and found 30.2-fold greater expression of Halo–p53-R273H (FL)–mCherry compared to Halo–p53 WT (FL)–mCherry (Fig. 2g). We next assessed whether the 30-fold increase in p53 protein abundance was targetable. Indeed, Halo-PEG2-BI2536 had no effect on the proliferation of Calu-1 p53 WT cells at doses ≤ 2 μM. However, in Calu-1 p53-R237H cells, we observed notable viability declines starting at 125 nM, with an IC_50_ = 402 nM after 4 days of treatment. BI2536 alone lacked a differential proliferation effect (Fig. 2h). We repeated this experiment for the *TP53*^Y220C^ mutation. Calu-1 *TP53*^Y220C^ cells displayed 18.6-fold greater mCherry expression than p53 WT counterparts with similar mTagBFP2 expression levels, corresponding to a ~4-fold therapeutic window for Halo-PEG2-BI2536 (Extended Data Fig. 2g,h). This change in protein abundance is consistent with the reported p53-Y220C in cellulo *t*_1/2_ (that is, in the presence of cellular chaperones) of 10–13 h from cycloheximide chase experiments^44^ (compared to the WT p53 *t*_1/2_ of minutes in quiescent cells), implying that Y220C destabilizes an active conformation of p53 rather than reducing p53 protein abundance.

Prior reports suggested that the enhanced efficacy of similar bifunctional compounds can occur because of the accumulation of small molecules in cells expressing high concentrations of the protein target^33^. We, therefore, modeled reaction–diffusion kinetics of Halo-PEG2-BI2536 using ordinary differential equations (Methods) to gain more insights into the enhanced toxicity observed in the presence of a ligandable p53 fusion protein. Previously reported increases in p53 protein *t*_1/2_ upon mutation greatly influence the expected accumulation of the bifunctional small molecule^28,29^. In particular, an increase in *t*_1/2_ from the WT p53 *t*_1/2_ of 16 min to a mutant p53 *t*_1/2_ of 17 h is expected to increase final intracellular concentration of the small molecule at steady state by 2–3 orders of magnitude depending on the initial concentration (Fig. 2i). Together, these data suggest that, if a bifunctional molecule combining a cytotoxic molecule with a small-molecule ligand of p53 were developed, the high protein abundance of mutant p53 would allow for differential cell killing.

### Bifunctional molecules selectively kill p53-Y220C^+^ cells

**Fig. 3:**
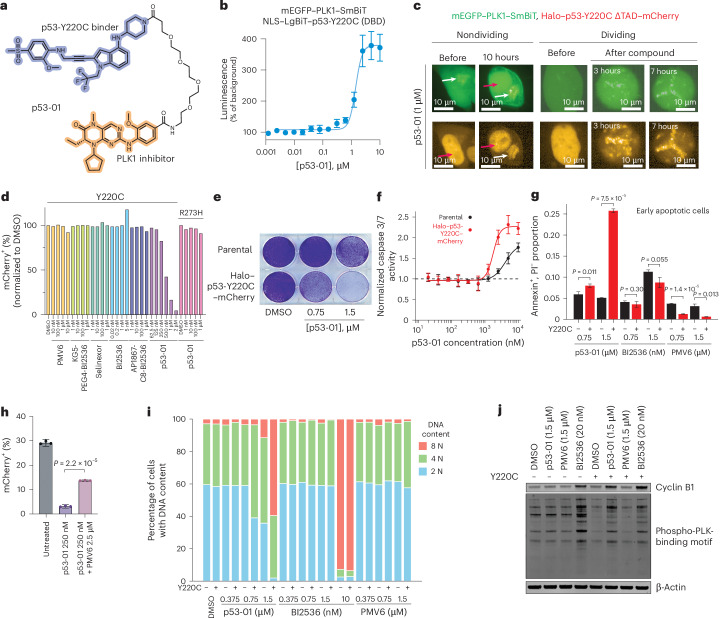
p53-Y220C–PLK1 bifunctional molecules selectively inhibit proliferation of *TP53*^Y220C^-mutant cells. **a**, Structure of p53-Y220C–PLK1 bifunctional compound (PMV6-PEG4-BI2536, p53-01) used in experiments. **b**, Nanoluciferase signal 1 day after dose titration of p53-01 in 293T cells cotransfected with NLS–LgBiT–p53-Y220C (DBD) and mEGFP–PLK1–SmBiT (*n* = 6 technical replicates per condition). **c**, Live-cell imaging of 293T cells cotransfected with Halo–p53-Y220C ΔTAD–mCherry and mEGFP–PLK1–SmBiT, treated with p53-01. The white arrow indicates the extranuclear region containing PLK1; the red arrow indicates the nucleus. Dividing and nondividing cells are shown. mCherry (p53) and EGFP (PLK1) channels are shown separately. Two replicates of this experiment were performed; a representative replicate is shown. **d**, Competition of 293T cells stably expressing Halo–p53-Y220C ΔTAD–mCherry versus parental 293T cells in the presence of various compounds (PMV6, KG5-PEG4-BI2536, selinexor, BI2536, AP1867-C8-BI2536 and p53-01). Right, competition of 293T cells stably expressing Halo–p53-R273H (FL)–mCherry versus parental 293T cells in the presence of p53-01. The mCherry^+^ percentage on day 8 of competition normalized to that of DMSO-treated cells is shown. Two replicates of this experiment were performed; a representative replicate is shown. **e**, Crystal violet staining of 293T cells stably expressing Halo–p53-Y220C ΔTAD–mCherry versus parental 293T cells treated with p53-01, analyzed after 5 days. The experiment was performed in triplicate; a representative replicate is shown. **f**, Caspase 3/7 glo of Halo–p53-Y220C ΔTAD–mCherry versus parental 293T cells treated with p53-01 after 1 day (*n* = 4 technical replicates per condition). **g**, Proportion of early apoptotic cells (annexin^+^, PI^−^) following 1 day of treatment with p53-01, BI2536 or PMV6 at the indicated concentrations in Halo–p53-Y220C ΔTAD–mCherry versus parental 293T cells. NS, not significant. **h**, Competition of 293T cells stably expressing Halo–p53-Y220C ΔTAD–mCherry versus parental 293T cells in the presence of p53-01 (250 nM) alone or in combination with PMV6 (2.5 μM). **i**, DNA content (assessed by flow cytometry of DAPI-stained cells) following treatment of palbociclib-synchronized Halo–p53-Y220C ΔTAD–mCherry versus parental 293T cells with p53-01, BI2536 or PMV6 at the indicated concentrations for 1 day. The experiment was performed in triplicate; one representative replicate is shown. **j**, Western blot for PLK1 targets including cyclin B1 and phospho-PLK-binding motif of double-thymidine-block-synchronized Halo–p53-Y220C ΔTAD–mCherry versus parental 293T cells treated with p53-01, BI2536 or PMV6 at the indicated concentrations for 8.5 h. Two replicates of this experiment were performed; a representative replicate is shown. Source data

**Extended Data Fig. 3:**
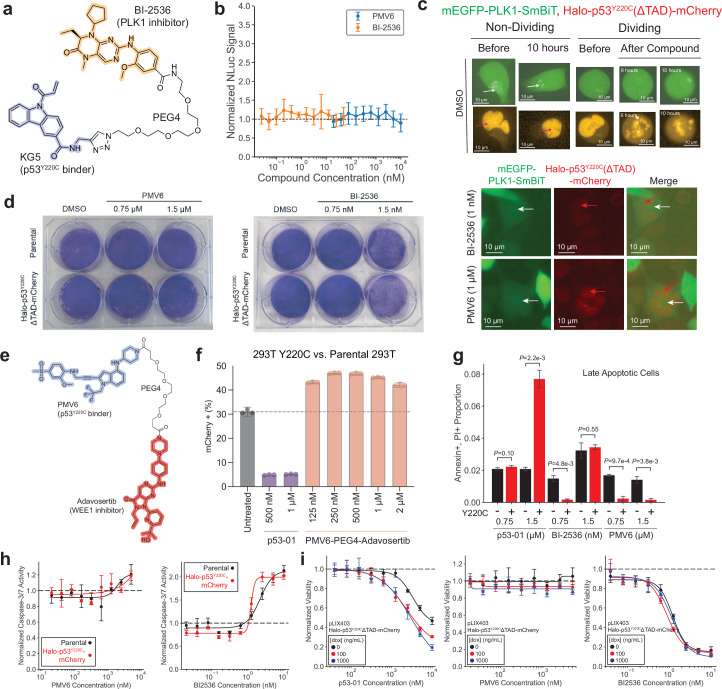
Cell killing is specific to p53-01. **a**, Structure of KG5-PEG4-BI2536. **b**, Normalized nanoluciferase signal one day after dose titration of PMV6 and BI-2536 in 293 T cells co-transfected with NLS-LgBiT-p53^Y220C^(DBD) and mEGFP-PLK1-SmBiT. N = 4 technical replicates/condition. **c**, Live cell imaging of 293 T cells co-transfected with Halo-p53^Y220C^ΔTAD-mCherry and mEGFP-PLK1-SmBiT and treated with DMSO, PMV6, or BI-2536 for 16 h. Two replicates of this experiment were performed; a representative replicate is shown. Scale bar is shown (10 μm). **d**, Crystal violet staining of 293 T cells stably expressing Halo-p53^Y220C^ΔTAD-mCherry versus parental 293 T cells treated with PMV6 or BI-2536, analyzed after 5 days. Experiment performed in triplicate; representative replicate shown. **e**, Structure of PMV6-PEG4-adavosertib. **f**, Competition of Halo-p53^Y220C^ΔTAD-mCherry vs. parental 293 T cells with p53-01 or PMV6-PEG4-adavosertib for 10 days; analyzed for mCherry expression by flow cytometry. N = 3 measurements/condition. **g**, Proportion of late apoptotic cells (Annexin+, PI+) following one day treatment with p53-01, BI-2536, or PMV6 at indicated concentrations in Halo-p53^Y220C^ΔTAD-mCherry vs. parental 293 T cells. n.s. not significant, **P < 0.01, ***P < 0.001 by t-test. N = 3 measurements/condition. **h**, Caspase-3/7 glo of Halo-p53^Y220C^ΔTAD-mCherry vs. parental 293 T cells treated with BI-2536 or PMV6 after one day. N = 4 technical replicates/condition. **i**, Normalized viability of 293 T cells expressing a dox inducible Halo-p53^Y220C^ΔTAD-mCherry treated with p53-01, PMV6, or BI-2536 for 4 days, induced with dox at various concentrations the day before beginning compound treatment (0 ng/mL, 100 ng/mL, 1000 ng/mL). N = 4 technical replicates/condition. Source data

To assess the impact of bifunctional molecules containing p53-binding and cytotoxic moieties in an endogenous cellular context, we synthesized bifunctional molecules that bind to p53-Y220C. This mutant indirectly inhibits DNA binding activity through the loss of DNA-binding domain (DBD) thermal stability^45^. The removal of a bulky tyrosine residue, creating a pocket, and its replacement with a ligandable cysteine make it an attractive target for small-molecule refolders meant to restore transcriptional activity^46–51^, as well as bifunctional cytotoxic strategies. We initially used acrylamide KG5 as the p53-Y220C binder in a PLK1-directed bifunctional; KG5 is a carbazole-based covalent fragment liganding > 95% of p53-Y220C at 10 μM (ref. ^49^). We synthesized KG5-PEG4-BI2536 (**3**) and did not observe any difference in cell survival between 293T cells stably expressing Halo–p53-Y220C ΔTAD–mCherry (transactivation domain deleted to remove confounding protein stabilization effects) and parental 293T cells in an 8-day competition assay (Fig. 3 and Extended Data Fig. 3a).

We surveyed the patent literature and identified a p53-Y220C-binding pharmacophore consisting of an *o*-anisidine moiety linked to an indole and piperidine (US20230024905A1, US10138219B2, WO2021262483A1, WO2021061643A1, WO2022213975A1 and WO2023016434A1). These binders are exemplified by PMV6 (**4**), a compound identified in a structure–activity relationship series by PMV Pharma, with close similarity to the recently disclosed structure of the investigational compound, rezatapopt, which has a *K*_d_ of ~2.5 nM for p53-Y220C (PMV6, p. 96: US20230024905A1)^52^. PMV6 induces an 8 °C thermal shift of p53-Y220C (p. 21: US20230024905A1). The cocrystal structure with p53-Y220C showed that the compound binds to a shallow groove created by the Y220C substitution, with the piperidine facing the solvent. To construct a bifunctional molecule containing PMV6 and BI2536, we functionalized PMV6 at the solvent-exposed piperidine, which was functionalized in other analogs that continued to bind p53-Y220C using a thermal shift assay (compounds 7 and 10, p. 21 and 96: US20230024905A1).

We synthesized PMV6-PEG4-BI2536 (**5**, p53-01; Fig. 3a) and confirmed that p53-01 but not binders PMV6 or BI2536 alone induce ternary complex formation between p53-Y220C and PLK1 using a NanoBiT assay in 293T cells cotransfected with mEGFP–PLK1–SmBiT and NLS–LgBiT–p53-Y220C (DBD) (Fig. 3b and Extended Data Fig. 3b; EC_50_ = 1.4 μM). As an alternative method to demonstrate ternary complex formation, we assessed protein colocalization upon compound treatment^53–56^. Live-cell imaging of 293T cells cotransfected with mEGFP–PLK1–SmBiT and Halo–p53-Y220C ΔTAD–mCherry revealed diffuse PLK1 localization throughout the cell, with strong enrichment in an extranuclear region (possibly the centrosome as previously reported^57^), whereas p53-Y220C was constitutively nuclear. In nonmitotic cells, p53-01 treatment resulted in PLK1 nuclear enrichment and colocalization with p53-Y220C, whereas p53-Y220C also entered the extranuclear region colocalizing with PLK1. In untreated mitotic cells, p53-Y220C appeared localized to chromatin, whereas PLK1 was localized elsewhere in the cell. Upon p53-01 treatment, PLK1 became colocalized with p53-Y220C on chromatin; binders alone had no impact on localization (Fig. 3c and Extended Data Fig. 3c). We speculate that this mislocalization could disrupt the function of PLK1 as a mitotic kinase beyond simple steric blockade of its active site.

We performed an 8-day growth competition experiment between Halo–p53-Y220C ΔTAD–mCherry and parental 293T cells and observed strong selection against p53-Y220C^+^ cells (mCherry^+^) when p53-01 was dosed between 250 nM and 2 μM (*E*_max_: 21.5-fold decline in the mCherry^+^ fraction; EC_50_ = 443 nM). We did not observe any activity from nonfunctionalized binders or control compounds, including PMV6, BI2536, AP1867-C8-BI2536 (**6**) or selinexor. We also did not observe any activity of p53-01 in either 293T or Calu-1 cells expressing Halo–p53-R273H (FL)–mCherry (Fig. 3d and Extended Data Fig. 2e). The differential antiproliferative activity of p53-01 in Halo–p53-Y220C ΔTAD–mCherry 293T cells was validated with crystal violet staining (Fig. 3e and Extended Data Fig. 3d). An analogous bifunctional molecule constructed with a WEE1 inhibitor (**7**, PMV6-PEG4-adavosertib) was unable to selectively inhibit proliferation of p53-Y220C^+^ cells (Extended Data Fig. 3e,f).

We next monitored for induction of apoptosis using caspase 3/7 glo and annexin–PI staining. p53-01 but not either binder alone differentially induced apoptosis in Halo–p53-Y220C ΔTAD–mCherry versus parental 293T cells (Fig. 3f,g and Extended Data Fig. 3g,h). To confirm the effects of our system were dependent on protein dosage, we used a doxycycline-inducible Halo–p53-Y220C ΔTAD–mCherry construct in 293T cells and were able to increase the potency of p53-01 approximately twofold without impacting the potency of either PMV6 or BI2536 (Extended Data Fig. 3i). To confirm that the effects were mediated by compound binding to p53-Y220C, we repeated the Halo–p53-Y220C ΔTAD–mCherry and parental 293T competition experiment using a tenfold excess of PMV6, observing partial rescue (p53-01 alone at 250 nM: 8.9-fold decline in mCherry^+^ percentage; p53-01 + PMV6 at 2.5 μM: 2.1-fold decline in mCherry^+^ percentage; *P* < 0.001; Fig. 3h).

PLK1 activity is critical for mitotic progression and its inhibitor, BI2536, was previously shown to induce G2/M arrest and genome doubling^58^. We monitored DNA content following treatment of synchronized Halo–p53-Y220C ΔTAD–mCherry and parental 293T cells with p53-01 or binders for 1 day. Low-dose p53-01 (0.75 μM) resulted in the accumulation of Halo–p53-Y220C ΔTAD–mCherry 293T cells with 4 N DNA content, leaving parental cells unaffected, whereas a higher dose (1.5 μM) induced genome doubling (accumulation of cells with 8 N DNA content) in ~60% of Halo–p53-Y220C ΔTAD–mCherry 293T but <10% of parental cells, phenocopying the high-dose BI2536 (10 nM) treatment (Fig. 3i). To confirm the effect was because of differential PLK1 inhibition, we performed western blots for PLK1 substrates including cyclin B1 (which accumulates upon PLK1 inhibition) and using an antibody to the phospho-PLK1-binding motif (PLK1-binding partners and substrates). p53-01 once again phenocopied BI2536 specifically in Halo–p53-Y220C ΔTAD–mCherry 293T but not in parental cells, with PMV6 having no effect (Fig. 3j). Together, these data indicate that a bifunctional molecule that both binds to p53-Y220C and binds to and inhibits PLK1 can selectively inhibit proliferation of cells expressing *TP53*^Y220C^ because of enhanced PLK1 inhibition, leading to mitotic arrest.

### Activity is p53-Y220C dependent without reactivation

**Fig. 4:**
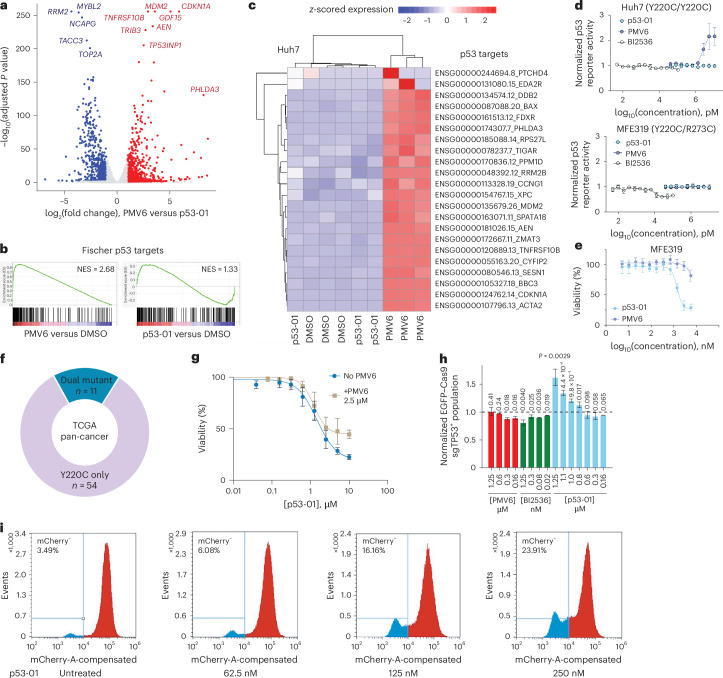
p53-Y220C–PLK1 bifunctionals are active in endogenous settings and do not reactivate p53. **a**, Huh7 cells homozygous for the *TP53*^Y220C^ mutation were treated with PMV6 (4 μM) or p53-01 (4 μM) for 24 h and subject to RNA-seq. The log_2_(fold changes) in transcript abundance and log_10_(adjusted *P* values) computed by DESeq2 are plotted. The top differentially expressed genes are labeled (*n* = 3 replicates per condition). **b**, Preranked GSEA using DESeq2 *t* statistic as rank metric for PMV6 versus DMSO (left) and p53-01 versus DMSO (right) comparisons on MSigDB Fischer gene set of direct p53 targets. NESs are listed. **c**, Gene expression analysis of p53 targets from high-confidence p53 target set. Samples are arranged in columns by hierarchical clustering (Euclidean distance) and rows scaled to *z* scores (colors). **d**, Luciferase activity following PMV6, BI2536 or p53-01 treatment (16 h) of Huh7 (left) or MFE319 (right) cell lines stably expressing a p53 luciferase transcriptional reporter. Luciferase signal is normalized to untreated cells (*n* = 6 technical replicates per condition for p53-01 and PMV6; *n* = 3 technical replicates per condition for BI2536). **e**, Cell viability of MFE319 cells treated with PMV6 or p53-01, measured by CTG after 4 days (*n* = 4 technical replicates per condition). **f**, Donut plot of cancers in TCGA Pan-Cancer Atlas studies in cBioPortal with *TP53*^Y220C^ mutations with or without additional *TP53* mutations. **g**, Cell viability of MFE319 cells treated with p53-01 alone or in combination with PMV6 (2.5 μM), measured by CTG after 4 days (*n* = 4 technical replicates per condition). **h**, Competition of MFE319 cells stably expressing sgTP53–Cas9–EGFP versus parental MFE319 cells in the presence of p53-01, PMV6 or BI2536. The EGFP^+^ percentage on day 9 of competition normalized to that of DMSO-treated cells is shown. **i**, Halo–p53-Y220C ΔTAD–mCherry 293T cells were cultured in the presence of p53-01 for 3 weeks. The percentage of mCherry^−^ cells was analyzed by FACS. Two replicates of this experiment were performed; a representative replicate is shown. Source data

**Extended Data Fig. 4:**
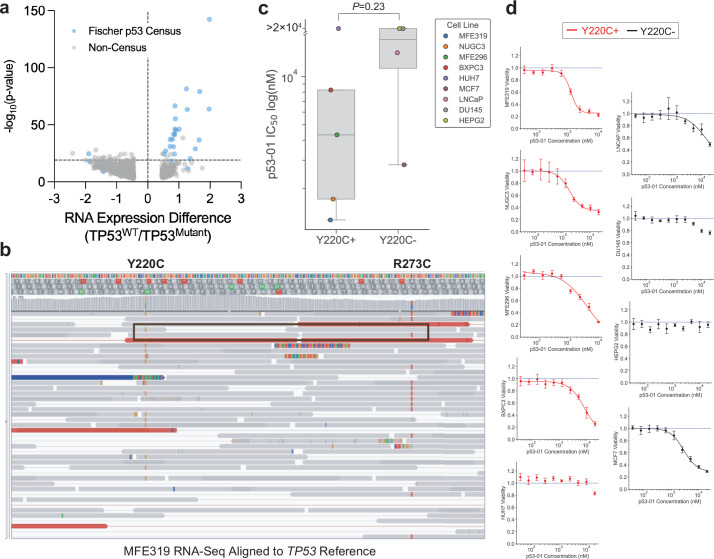
p53-01 is active in endogenous settings. **a**, Difference in mRNA expression (log_2_FC) between *TP53* WT and mutant cell lines in DepMap for all genes. *P*-values computed by t-test. Fischer census p53 targets are colored in blue. **b**, Analysis of RNA-seq reads aligned to p53 transcript sequence in MFE319 and visualized in IGV. Paired reads boxed in red span residues 220-273 demonstrate the Y220C and R273C mutations are present in *trans*. **c**, Distribution of log(IC_50_) of p53-01 across a panel of Y220C+ (MFE319, NUGC-3, MFE296, BxPC-3, Huh7) and Y220C- cell lines (MCF7, LNCaP, DU145, HepG2) after 4 day treatment. *P*-value computed by heteroscedastic t-test; n.s., not significant. **d**, Individual viability-dose curves for p53-01 across a panel of Y220C+ (MFE319, NUGC-3, MFE296, BxPC-3, Huh7) and Y220C- cell lines (MCF7, LNCaP, DU145, HepG2) after 4 day treatment. Cell lines in red are Y220C+, those in black are Y220C-. N = 4 technical replicates/condition. Source data

We next analyzed whether the mechanism of action for p53-01 is distinct from that for PMV6, a reactivator of p53-Y220C. We performed RNA sequencing (RNA-seq) of Huh7 hepatocellular carcinoma cells (homozygous *TP53*^Y220C/Y220C^) treated with p53-01 (4 μM) and PMV6 (4 μM) for 1 day. We observed strong induction of a p53 signature by PMV6, including >10-fold upregulation of p53 targets such as *MDM2*, *CDKN1A*, *GDF15* and PUMA (*BBC3*) and downregulation of genes essential for proliferating cells such as *TOP2A*. Manual inspection of a set of high-confidence p53 transcriptional targets and gene set enrichment analysis (GSEA) of a larger group of consensus p53 targets confirmed strong upregulation of these genes by PMV6 (normalized enrichment score (NES) = 2.68) but not p53-01 (NES = 1.33) (Fig. 4a–c and Extended Data Fig. 4a). Thus, p53-01 does not appear to act through a transcriptional mechanism of p53 reactivation.

We subsequently established p53-Y220C^+^ cell lines stably expressing a p53 luciferase reporter^59^. In Huh7 cells (*TP53*^Y220C/Y220C^), PMV6 was able to induce activity of the reporter at doses of 1.25–10 μM (EC_50_ = 2.4 μM), whereas p53-01 and BI2536 had no such effect. We repeated the p53 reporter experiments in the p53-Y220C^+^ endometrial cancer cell line MFE319, where no induction of luciferase activity for any compound (p53-01, PMV6 or BI2536) was observed (Fig. 4d). Note that MFE319 has both *TP53*^Y220C^ and *TP53*^R273C^ mutations reported in the CCLE dataset^35^. Analysis of paired RNA-seq reads in this cell line revealed that the mutations were in *trans* (Extended Data Fig. 4b). It is possible that the additional dominant negative *TP53* mutation in this cell line prevents p53 reactivation by p53-Y220C refolders (for example, PMV6), plausibly because of poisoning of the tetramer even in the presence of correctly functioning p53-Y220C proteins. The strategy presented in this paper, using p53-Y220C to concentrate a toxin in cells, is unlikely to be impacted by the additional *TP53*^R273C^ mutation. This would result in a ~20% greater scope of treatable cancers for PLK1-p53-Y220C bifunctional molecules compared to p53-Y220C reactivators (Fig. 4e,f; as determined by analysis of *TP53*^Y220C^-mutant cancers in TCGA^60^ with additional *TP53* mutations).

Lastly, we wanted to assess whether the effects of p53-01 in MFE319 were dependent on its interaction with p53-Y220C. Indeed, we were able to partially compete the decline in cell viability induced by p53-01 in MFE319 using PMV6 (Fig. 4g). Additionally, we established MFE319 *TP53*-knockout cells by stably expressing sgTP53–Cas9–EGFP. In competition with parental MFE319 cells, p53-01 was able to significantly enrich an EGFP^+^ population at doses between 800 nM and 1.25 μM. BI2536 and PMV6 had no such activity (Fig. 4h). As further evidence of on-target activity, we cultured Halo–p53-Y220C ΔTAD–mCherry 293T cells for 3 weeks in the presence of low doses of p53-01 (≤250 nM) and observed the expansion of an mCherry^−^ population (Fig. 4i; 6.9-fold expansion of mCherry^−^ fraction at 250 nM). Furthermore, p53-01 trended toward superior activity across a panel of p53-Y220C^+^ cancer cell lines compared to p53-Y220C^−^ counterparts (Extended Data Fig. 4c,d). Altogether, these data indicate that p53-01 has activity in cell lines expressing *TP53*^Y220C^, distinct from PMV6 (not through p53 reactivation), and its activity is dependent on its interaction with p53-Y220C.

### Linker optimization improves efficacy of bifunctionals

**Fig. 5:**
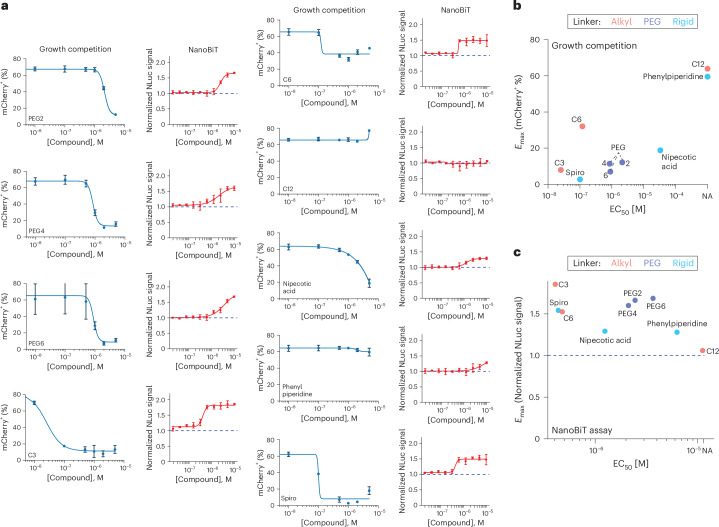
Linker optimization yields more potent p53-Y220C–PLK1 bifunctionals. **a**, Left, mCherry percentage versus dose for individual compounds from the growth competition assay between Halo–p53-Y220C ΔTAD–mCherry versus parental 293T cells on day 10. Right, normalized nanoluciferase signal from NanoBiT assay versus dose for individual compounds. NanoBiT experiments were set up in 293T cells cotransfected with NLS–LgBiT–p53-Y220C (DBD) and mEGFP–PLK1–SmBiT, treated with compound for 24 h (*n* = 3 replicates per condition for viability competition; *n* = 4 replicates per condition for NanoBiT). **b**, EC_50_ from the growth competition experiment is plotted against *E*_max_ (calculated as the lowest percentage of mCherry^+^ cells in the competition assay observed at a tested dose). **c**, EC_50_ from the NanoBiT experiment is plotted against *E*_max_ (maximum normalized luciferase signal observed at a tested dose, normalized to DMSO-treated cells). Source data

We created a library of nine PMV6-BI2536 bifunctional compounds with varying linker lengths (4.33–22.22 Å, compounds **5** and **8**–**15**) and assessed their efficacy in competition assays between parental and Halo–p53-Y220C ΔTAD–mCherry 293 T cells (Table 1). We observed no consistent effect of linker length on compound efficacy in general, although shorter alkyl linkers outperformed longer alkyl linkers. PEG(2,4,6) linkers all performed similarly. The most potent compound, PMV6-C3-BI2536 (**10**, β-alanine linker), was over 1.5 orders of magnitude more potent than p53-01 in the competition assay (PMV6-C3-BI2536 EC_50_: 25 nM, p53-01 EC_50_: 850 nM; Fig. 5a,b). Activity of compounds strongly correlated with their ability to form ternary complexes in the mEGFP–PLK1–SmBiT and NLS–LgBiT–p53-Y220C (DBD) NanoBiT system (Fig. 5a,c). These data indicate that substantial improvements to the efficacy of p53-Y220C–PLK1 bifunctional compounds are possible going forward.

**Table 1:** Effect of linker on activity of p53-Y220C–PLK1 bifunctionals

Linker	Length (Å)	Linker type	Competition EC_50_ (µM)	NanoBiT EC_50_ (µM)
PEG2	10.96	PEG	2.13	2.45
PEG4	16.01	PEG	0.85	2.12
PEG6	22.22	PEG	0.90	3.68
C12	12.71	Alkyl	NA	NA
C6	8.08	Alkyl	0.12	0.48
C3	4.33	Alkyl	0.025	0.41
Nipecotic acid	5.96	Rigid	33.2	1.24
Phenylpiperidine	9.35	Rigid	NA	6.28
Azaspiroheptane	6.14	Rigid	0.101	0.44

Summary of linkers tested. The linker length, EC_50_ from Halo–p53-Y220C ΔTAD–mCherry versus parental 293T growth competition and EC_50_ from NanoBiT experiment are listed (*n* = 3 replicates per condition for viability competition; *n* = 4 replicates per condition for NanoBiT). Growth competition assays were analyzed on day 10. NanoBiT experiments were set up in 293T cells cotransfected with NLS–LgBiT–p53-Y220C (DBD) and mEGFP–PLK1–SmBiT, treated with compound for 24 h. NA indicates that the EC_50_ could not be computed because of a lack of potency.

## Discussion

*TP53* mutations remain the dominant mutation associated with death from human cancers^5^. While there have been prior reports of mutation-agnostic small-molecule refolders of p53, these molecules have later been found to lack the desired activity^17,18^. Our strategy reveals one critical insight: p53-targeted drugs need not restore native p53 function but can instead use differential p53 protein abundance or mutant-specific ligands to bring about a *TP53*-mutant selective therapy. Here, we use the high intracellular concentration of the missense mutant p53 protein to induce cancer-selective cell death. Our work is conceptually aligned with RIPTACs but uses p53 as a target protein instead of lineage oncogenes such as androgen receptor^33^. Selectivity arises from the pathognomonic overabundance of missense mutant p53 (a broken MDM2 feedback loop) rather than lineage expression and selectivity can be made mutant specific using a Y220C binder, enabling killing without restoring canonical p53 transcriptional activity.

The molecules in this report are far from pharmacologically optimized. We anticipate that more potent and selective molecules may be achieved in the future. Because the bivalent compounds presented here include a nonspecific small-molecule toxin, they are able to induce toxicity in cells that do not bear *TP53* mutations when administered at sufficiently high doses. Future generations of molecules may have no toxicity in the absence of p53 proteins by working like other molecular glues, using mutant p53 as a ‘presenter protein’ whose surface may form a complementary interaction with essential components of cellular machinery. The prior observation that members of the manumycin polyketide family of natural products are molecular glues between p53 and UBR7 (ref. ^61^) indicates that molecular glues involving p53 are indeed possible.

It is important to recognize that the compounds we describe are only capable of binding to the Y220C mutant of p53, which possesses a unique cavity not found on the native p53 protein or other mutants. More work will be needed to extend this strategy to other missense mutants. Future p53-selective therapies may bind to protein folds found on WT p53 proteins^62^ and use elevated p53 protein abundance to generate compounds that selectively target the majority of cancers carrying *TP53* missense mutations. Because most sources of cell stress can induce the accumulation of p53, the optimal pan-p53-selective compounds are likely to be covalent and dosed in pulses such that the therapeutic window between cancer and normal tissue is maximized. PLK1 inhibition in cells with high levels of WT p53 may be tolerated as these cells could be growth arrested. Other future compounds may bind selectively to mutant p53 proteins whose surface allows for selective small-molecule binding. Furthermore, targeting certain essential genes may promote the accumulation of mutant p53 protein, as was shown for WEE1 degraders recently^63^, possibly enabling larger therapeutic windows.

If clinical-grade compounds that can specifically kill cells with increased p53 abundance can be generated, they may enjoy roles outside of cancer therapy. In one example, p53 is highly expressed in senescent cells and deficiency of MDM2 causes a progeroid syndrome in humans^64,65^. Future p53-targeting compounds of this class could function as senolytics.

New targeted therapies are inevitably followed by the emergence of on-target and off-target resistance mechanisms. As we demonstrated, cancer cells evolved resistance by decreasing mutant p53 protein expression upon chronic exposure to intermediate doses of these compounds. These results reflect the observation that antigen loss follows exposure to cytotoxic agents whose activity depends on antigen presence. Nonetheless, we hope that, in the fullness of time, gain-of-function p53-selective small molecules will allow for sufficient specific killing of cancer cells such that, in combination with other therapies, a greater fraction of persons will achieve meaningful remissions.

## Methods

### General methods

Measurements and experiments were performed in triplicate unless otherwise specified. All statistical tests were two-tailed; heteroscedastic *t*-tests were used for pairwise comparisons unless otherwise specified. Points with error bars are plotted as the mean ± s.d. unless otherwise specified; lines in box plots represent the first quartile to 1.5× the interquartile range (IQR), the first quartile, the median, the third quartile and the third quartile to 1.5× the IQR.

### Cancer genomics analyses

CRISPR dependency scores, RPPA *z*-scored protein expression, RNA-seq, *TP53* mutation calls and compound sensitivity data were downloaded from the DepMap portal (https://depmap.org/portal/)^37^. Human cell surface proteins (*n* = 1,492) were previously reported^67^. Protein abundance in 293T cells was downloaded from OpenCell^38^. Immunohistochemistry images were downloaded from the Human Protein Atlas^66^. To identify genes that were both essential and had low protein abundance, we filtered to those with mean Chronos scores ≤ −2 and with protein abundance ≤ 920 nM (p53 protein abundance in 293T cells). We ranked the remaining genes on the basis of log_10_(protein concentration) + mean Chronos score and identified those with the lowest score using this metric.

### MFE319 *TP53* mutation analysis

MFE319 paired RNA-seq reads were downloaded from the CCLE (SRR8615235)^35^. The reads were aligned directly to the p53 major isoform mRNA transcript fasta (NM_000546.6) using STAR^68^. The resulting BAM file was visualized in Integrative Genomics Viewer^69^. Read pairs spanning both mutant residues (Y220C and R273C) were used to infer the mutations were in *trans*.

### Modeling of compound accumulation

We assume that the cell membrane is permeable to the bifunctional small molecule, allowing it to freely diffuse. We also assume that p53 is in greater abundance than the other target bound by the bifunctional small molecule, such that the binding of the other target does not contribute meaningfully to intracellular trapping of the molecule. We define the following variables: [M_A0_], the initial concentration of the bifunctional small molecule outside of the cell (which is a parameter we vary); [M_B0_] = 0, the initial concentration of the bifunctional small molecule inside the cell; [P_0_] = 1 × 10^−6^ M, the initial intracellular concentration of p53 (defined according to abundance from OpenCell^38^); [MP_0_] = 0, the initial intracellular concentration of the molecule–p53 complex. We also define constants for diffusion (*k*_diff_ = 1 × 10^3^ s^−1^), binding of a pharmaceutically optimized bifunctional molecule to p53 (*k*_bind_ = 8.7 × 10^5^ M^−1^ s^−1^), dissociation of a pharmaceutically optimized bifunctional molecule from p53 (*k*_unbind_ = 1 × 10^−6^ s^−1^) and p53 protein *t*_1/2_ (which is a parameter we vary).

We define the following rates:

d[M_A_]/dt = 0

d[M_B_]/dt = −*k*_diff_[M_B_] + *k*_diff_[M_A_] − *k*_bind_[M_B_][P] + *k*_unbind_[MP]

d[MP]/dt = *k*_bind_[M_B_][P] − *k*_unbind_[MP] − [MP] × (ln 2)/(*t*_1/2_)

d[P]/dt = −*k*_bind_[M_B_][P] + *k*_unbind_[MP] − [P] × (ln 2)/(*t*_1/2_) + *r*_p53 production_

Note that the constant rate of p53 protein production, *r*_p53 production_, was estimated using the concentration of p53 missense mutant protein at steady state in a cell.

d[p53]/dt = protein production rate − protein degradation rate = 0

d[p53_missense_]/dt = protein production rate − [p53_missense_] × ln(2)/*t*_1/2-p53missense_ = 0

Protein production rate = [p53_missense_] × ln(2)/*t*_1/2-p53missense_, with *t*_1/2-p53missense_ = 24 h

We ran an ODE solver in R (package: deSolve) using these parameters and calculated final intracellular ([M_B_] + [MP]) and extracellular ([M_A_]) bifunctional small-molecule concentrations at *t* = 600 h.

### Cell culture

The 293T (CRL-3216), Calu-1 (HTB-54), BxPC-3 (CRL-1687), HepG2 (HB-8065), DU145 (HTB-81), LNCaP (CRL-1740) and MCF7 cells (HTB-22) were obtained from the American Type Culture Collection; the MFE319 (ACC 423) and MFE296 (ACC 419) cells were obtained from the German Collection of Microorganisms and Cell Cultures; the Huh7 (JCRB0403) and NUGC-3 (JCRB0822) cells were obtained from the Japanese Collection of Research Bioresources. All cell lines were cultured in DMEM supplemented with 10% FBS, 100 IU per ml penicillin and 100 μg ml^−1^ streptomycin at 37 °C in 5% CO_2_.

### Plasmids

Plasmids were ordered as codon-optimized entry vectors from TWIST (pTwist-ENTR). This includes the following constructs: Halo–p53-R273H (FL)–mCherry, Halo–p53-Y220C (ΔTAD)–mCherry, Halo–p53-Y220C (FL)–mCherry, Halo–p53 WT (FL)–mCherry–2A–mTagBFP2-V5, Halo–p53-R273H (FL)–mCherry–2A–mTagBFP2-V5, Halo–p53-Y220C (FL)–mCherry–2A–mTagBFP2-V5, NLS–LgBiT–p53-Y220C (DBD) and mEGFP–PLK1–SmBiT. p53-Y220C was ordered with stabilizing substitutions (M133L;V203A;N239Y;N268D)^45^ in all cases except for the Halo–p53-Y220C (FL)–mCherry–2A–mTagBFP2-V5 construct. Plasmids were Gateway-cloned into lentiviral EF1a expression vector pLEX307 or pLIX403 using LR clonase II (Invitrogen). The p53 reporter (Addgene, 90363)^59^ and Cas9–EGFP plasmid (Addgene, 82416) were purchased from Addgene^70^. DNA oligos encoding the *TP53* sgRNA (top strand: CACCGCAGAATGCAAGAAGCCCAGA, bottom strand: AAACTCTGGGCTTCTTGCATTCTGC) were ordered from Azenta and restriction-cloned into the Cas9–EGFP plasmid. Sequences were verified through PlasmidSaurus whole-plasmid sequencing.

### Transient transfection and stable cell line creation

Plasmids were transfected into 293T cells using TransIT-LT1 transfection reagent following the manufacturer’s protocol. Lentivirus was generated transfecting psPAX2 (Addgene, 12260), pMD2.G (Addgene, 12259) and the cloned pLEX307/pLIX403 plasmid (Addgene, 41392) (3:3:2 ratio) into 293T cells. Lentivirus was collected 2 days after transfection and stable cell lines were established infecting with filtered lentivirus and polybrene (10 μg ml^−1^). Cells were switched to selection medium 2 days after infection.

### CellTiter-Glo (CTG)

Cells were plated in 384-well plates (typically 1,500 cells per well) with varying concentrations of compounds, with a total volume of 50 μl per well. CTG reagent was added (25 μl) to the solutions, mixed and read out using an EnVision 2105 multimode plate reader.

### Crystal violet

The 293T cells were plated in six-well or 12-well plates at low density (~10% confluence) and treated with compound for the indicated duration. Cells were rinsed once with PBS and then stained with crystal violet (0.5% m/v) in 20% methanol–water for 10 min. Cells were washed with water five times and air-dried overnight.

### Flow-cytometry-based competition assays

Cells with and without a fluorescent marker (for example, Halo–p53–mCherry) were mixed and the resulting solution was plated in six-well plates or 10-cm dishes at low density; compound was added at varying concentrations. After reaching confluence, cells were passaged and retreated with compound if needed. Otherwise, cells were trypsinized, washed and resuspended in complete growth medium in 96-well plates or tubes for flow cytometry. Untreated cell mixtures were used to establish gates separating the fluorophore^+/−^ populations. Analysis was conducted on a CytoFLEX LX flow cytometer.

### NanoBiT assay

The 293T cells were seeded in six-well plates and cotransfected with NLS–LgBiT–p53-Y220C (DBD) and mEGFP–PLK1–SmBiT (1:1). After 1 day, the cells were passaged into 384-well plates and treated with varying concentrations of compound. Then, 1 day after treatment, nanoluciferase substrate was added to the cells and luminescence was monitored using an EnVision 2105 multimode plate reader.

### Colocalization assay and microscopy

The 293T cells were seeded in six-well plates and cotransfected with Halo–p53-Y220C ΔTAD–mCherry and mEGFP–PLK1–SmBiT (1:1). After 1 day, the cells were passaged into 96-well plates in FluoroBrite DMEM for microscopy. The cells were imaged with the Opera Phenix Plus high-content screening system (PerkinElmer) before compound treatment and as a time series following compound treatment.

### Caspase 3/7 glo

Cells were plated in 384-well plates (typically 1,500 cells per well) with varying concentrations of compounds with a total volume of 50 μl per well. After 1 day, caspase 3/7 glo reagent (Promega) was added following the manufacturer’s protocol and read out using an EnVision 2105 multimode plate reader.

### Annexin–PI staining

Cells were plated in six-well plates and treated with varying concentrations of compounds for 1 day. Cells were stained with annexin V–AF488 and PI following the manufacturer’s instructions (Thermo Fisher, V13241) and analyzed by flow cytometry (Beckman CytoFLEX LX), using untreated cells to establish gates.

### Cell-cycle analysis

Cells were split into a 10-cm dish at low confluency (20%) and grown overnight. Cells were synchronized using overnight treatment with palbociclib (150 nM), washed and split into a six-well plate for treatment with compound for 24 h. Cells were harvested by trypsinization, washed twice with cold 1× PBS, fixed by dropwise addition of ice-cold 70% ethanol while vortexing and incubated overnight (4 °C). Fixed samples were centrifuged at 1,000*g* for 5 min and 70% ethanol was removed. Cells were washed twice with 1× PBS + 1% BSA before DNA labeling with 1 µg ml^−1^ DAPI for 10 min at room temperature, before being analyzed for DNA content by flow cytometry (Beckman CytoFLEX LX).

### Western blotting

Cells were split into a 10-cm dish at low confluency (20%) and grown overnight. Cells were synchronized with double thymidine block. Cells were treated with thymidine (2 mM) overnight and released into fresh medium for 8.5 h and then retreated with thymidine (2 mM) overnight. Cells were split into a six-well plate and released into medium containing compound for 8.5 h. Cells were pelleted and protein lysates were created using RIPA buffer containing Halt phosphatase and protease inhibitor (Thermo Fisher, 78440). Lysates were clarified by centrifugation (21,000*g* for 15 min) and the supernatant was denatured in 1× Lamelli buffer containing DTT by heating at 95 °C for 5 min. The resulting protein was loaded onto a Tris–acetate gel, transferred onto a PVDF membrane and treated with primary antibodies: cyclin B1 (D5C10, Cell Signaling Technologies, 12231T; 1:1,000) and phospho-PLK-binding motif (D73F6, Cell Signaling Technologies, 5243T; 1:1,000). The membrane was stained with IRDye 800CW goat anti-rabbit IgG secondary antibody (Licor Biosciences; 1:20,000) and imaged on an Odyssey DLx.

### Huh7 RNA-seq and analysis

Huh7 cells were passaged into six-well plates and treated with PMV6 (4 μM), p53-01 (4 μM) or DMSO for 24 h. Cells were washed once with cold PBS. Subsequently, TRIzol (Invitrogen) was added to cells and, following the manufacturer’s protocol, RNA was extracted. RNA concentration was monitored using a Qubit fluorometer (Thermo Fisher) and RNA integrity was analyzed using an Agilent Bioanalyzer. The NEBNext Ultra II RNA library prep kit (Illumina) was used to prepare an RNA-seq library. A NovaSeq 6000 machine (Illumina) was used for paired-end 150-bp RNA-seq. STAR/RSEM^68,71^ was used to align RNA-seq reads to the GENCODE v38 transcript ref.^72^ and generate a count matrix. Raw counts were rounded and DESeq2 was used for downstream analysis^73^. A preranked GSEA^74^ was performed using the DESeq2 *t* statistic (versus DMSO-treated cells) as a rank metric using the Fischer p53 targets geneset^75^.

### P53 reporter assay

Huh7 and MFE319 cell lines stably expressing a p53 reporter (Addgene, 90363)^59^ were generated. Cells were plated in 384-well plates (1,500 cells per well) and treated with varying concentrations of compound. Then, 16 h after treatment, firefly luciferase substrate was added to the cells and luminescence was monitored using an EnVision 2105 multimode plate reader.

### RT–qPCR

Calu1 cells stably expressing Halo–p53 (FL)–mCherry–2A–mTagBFP2-V5 were passaged into six-well plates. RNA was extracted using a RNeasy Plus mini kit (Qiagen) following the manufacturer’s instructions. RNA (500 ng) was reverse-transcribed to complementary DNA (cDNA) using SuperScript VILO master mix following the manufacturer’s instructions (total volume: 20 μl). The resulting solution was diluted 1:5 with water and 2 μl of cDNA was used in each qPCR reaction in 384-well PCR plates (AB1384W). Then, 2× Power SYBR green PCR master mix (Fisher) and primers (150 nM final concentration) were added to a total volume of 15 μl. Primers used were as follows: Halo–p53 (FL)–mCherry–2A–mTagBFP2-V5 forward, CAGGACGGCTGCCTTATTTA; Halo–p53 (FL)–mCherry–2A–mTagBFP2-V5 reverse, AGACGGCAGATCGCAATATC; GAPDH forward, GTCTCCTCTGACTTCAACAGCG; GAPDH reverse, ACCACCCTGTTGCTGTAGCCAA. Amplification was performed following the manufacturer’s protocol on a QuantStudio 7 Flex real-time PCR system.

### Chemical synthesis

The synthesis and characterization of the small molecules reported in this paper are described in Supplementary Note 1.

### Reporting summary

Further information on research design is available in the Nature Portfolio Reporting Summary linked to this article.

## Online content

Any methods, additional references, Nature Portfolio reporting summaries, source data, extended data, supplementary information, acknowledgements, peer review information; details of author contributions and competing interests; and statements of data and code availability are available at 10.1038/s41589-025-02051-7.

## Supplementary information

Supplementary Information Supplementary Fig. 1 and Note 1.

Reporting Summary

## Source data

Source Data Fig. 2 Statistical source data.

Source Data Fig. 3 Statistical source data.

Source Data Fig. 3 Uncropped blots.

Source Data Fig. 4 Statistical source data.

Source Data Fig. 5 Statistical source data.

Source Data Extended Data Fig. 2 Statistical source data.

Source Data Extended Data Fig. 3 Statistical source data.

Source Data Extended Data Fig. 4 Statistical source data.

## Data Availability

The data supporting the findings of this study are available within the paper and its Supplementary Information. RNA-seq raw data (FASTQ, BAM) were deposited to Dropbox (https://www.dropbox.com/scl/fo/1c2c9wvccret30y0j8x7w/AKw3XZ5S0YjUnMws3Me6gOM?rlkey=n83j73hitpkamvar9pyfeknoz&st=pye3w9kw&dl=0). Should any raw data files be needed in another format, they are available from the corresponding author upon reasonable request. Source data are provided with this paper.
