# Publisher Correction: Mutant p53 protein accumulation is selectively targetable by proximity-inducing drugs

**DOI:** 10.1038/s41589-025-02095-9

**Published:** 2025-11-12

**Authors:** Ananthan Sadagopan, Maximilian Carson, Eriks J. Zamurs, Nicholas Garaffo, Heng-Jui Chang, Stuart L. Schreiber, Matthew Meyerson, William J. Gibson

**Affiliations:** 1 Broad Institute of MIT and Harvard, Cambridge, MA USA; 2 Department of Medical Oncology, Dana-Farber Cancer Institute, Boston, MA USA; 3 Division of Medical Sciences, Harvard Medical School, Boston, MA USA; 4 Department of Chemistry and Chemical Biology, Harvard University, Cambridge, MA USA; 5 Department of Medicine, Harvard Medical School, Boston, MA USA

**Keywords:** Cell death, Small molecules, Cancer therapy, Cell signalling

Correction to: *Nature Chemical Biology* 10.1038/s41589-025-02051-7, published online 3 November 2025.

In the version of the article initially published, the *x*-axis label for Fig. 4e was “log_10_(concentration), pM” but should have been “log_10_(concentration), nM” and has now been corrected in the HTML and PDF versions of the article.

