# Supplementary material for: Mutant p53 protein accumulation is selectively targetable by proximity-inducing drugs
Source: Nat Chem Biol. 2025 Nov 3;22(5):783–92. doi: 10.1038/s41589-025-02051-7 (PMC13128454; doi:10.1038/s41589-025-02051-7)
Supplement: Supplementary file 1 — Supplementary Fig. 1 and Note 1. [file 41589_2025_2051_MOESM1_ESM.pdf]

nature chemical biology

Supplementary information

https://doi.org/10.1038/s41589-025-02051-7

Mutant p53 protein accumulation is selectively targetable by proximity-inducing drugs

In the format provided by the authors and unedited

SUPPLEMENTARY INFORMATION

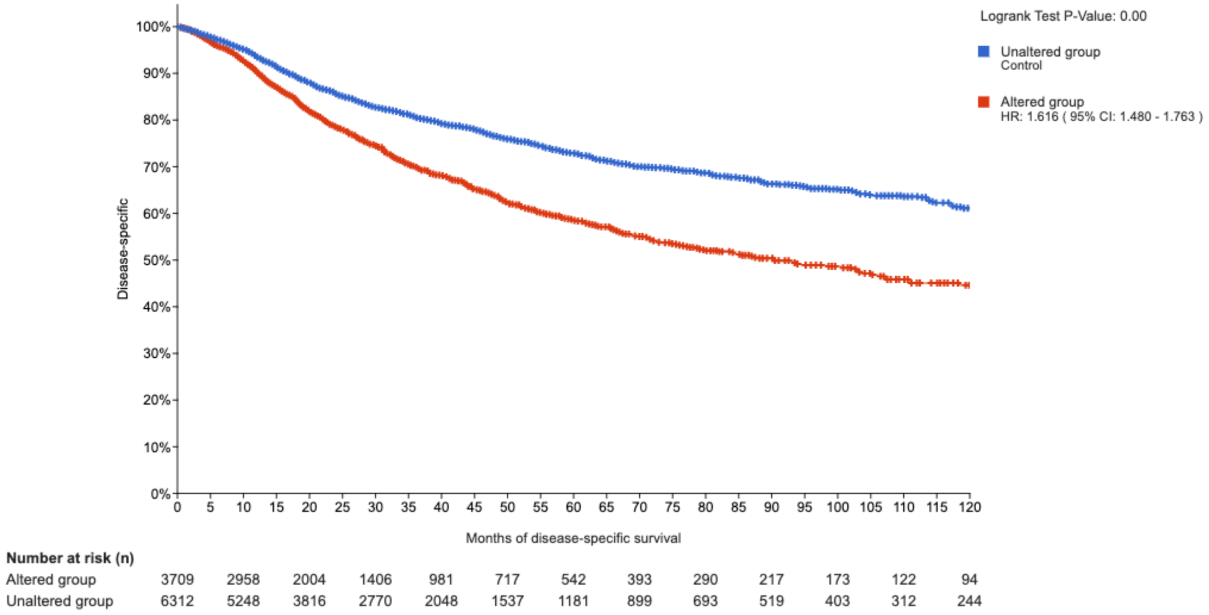

Supplementary Figure 1 | TP53 mutant cancers are associated with approximately half of cancer deaths. To estimate the fraction of cancer deaths that occur in patients with detectable TP53 mutations, we queried the cBioPortal database[7,8], combining all non-redundant studies. This query retrieved 224 studies, 80,085 samples from 76,363 patients. 32.1% of patients harbored a TP53 mutation (28,102 total mutations detected). Survival data were only available for 33,176 patients, 10,350 of whom had a TP53 alteration (31.2%). Disease specific survival data were only available for 10,021 patients, 3,709 of whom had a TP53 alteration (37.0%). The 10-year disease-specific survival rate was 61.10% in the TP53 WT group and 44.60% in the TP53 mutation group (HR: 1.62; CI-95%: 1.48-1.763; $p<1e-10$). The number of disease-specific deaths that occurred in the TP53 mutant group was 1,016 and the number of deaths in the WT group was 1,212. The fraction of cancer-specific deaths that occurred in TP53 mutant patients was 45.6%.

Supplementary Note 1

General Procedures. Commercial reagents, without additional purification, were used for all syntheses. Round-bottom flasks were used for reactions with Teflon-coated magnetic stir bars. Reaction progress was monitored using ultra-performance liquid chromatography mass spectrometry on a Waters ACQUITY UPLC I-Class 15 PLUS System with an ACQUITY SQ Detector 2. Nuclear magnetic resonance (NMR) spectra were generated at the Broad Institute of MIT and Harvard, using a Bruker AVANCE III HD 400 MHz spectrometer at room temperature (^{1}H NMR, 400 MHz; ^{13}C NMR 101 MHz). Chemical shifts for 1H NMR and 13C NMR are given in parts per million (ppm), referenced to residual solvent signals. DMSO-d6 and CD_3CN were acquired from Cambridge Isotope Laboratories, Inc. and Oakwood Products, Inc., respectively. ^{1}H NMR data is reported as follows: chemical shift value in ppm, multiplicity (s = singlet, d = doublet, t = triplet, dd = doublet of doublets, and m = multiplet), coupling constant value in Hz, and integration value. Electrospray ionization-high-resolution mass spectrometry (ESI-HRMS) was performed at the Harvard Center for Mass Spectrometry.

Organic Synthesis:

PMV6.

16 **17** **18** **4**

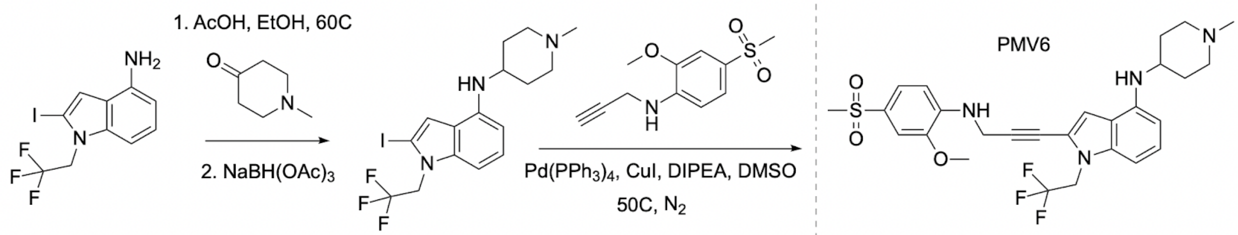

2-(3-((2-methoxy-4-(methylsulfonyl)phenyl)amino)prop-1-yn-1-yl)-N-(1-methylpiperidin-4-yl)-1-(2,2,2-trifluoroethyl)-1H-indol-4-amine (PMV6, PMV6-NMe). To a solution of 2-iodo-1-(2,2,2-trifluoroethyl)-1H-indol-4-amine (50 mg, 0.147 mmol, eNovation Chemicals) in ethanol (5 mL), 1-methyl-4-piperidone (100 µL, 0.867 mmol) and acetic acid (50 µL, 0.874 mmol) were added, and the mixture was heated to 60 °C with strong stirring. After 30 minutes, sodium triacetoxyborohydride (300 mg, 1.415 mmol) was added and allowed to react overnight. After completion, the reaction was purified by reverse-phase HPLC (acetonitrile:water gradient up to 90%) to yield 2-iodo-N-(1-methylpiperidin-4-yl)-1-(2,2,2-trifluoroethyl)-1H-indol-4-amine as an off-white solid (26 mg, 0.0595 mmol, 40.5% yield). Subsequently, 2-iodo-N-(1-methylpiperidin-4-yl)-1-(2,2,2-trifluoroethyl)-1H-indol-4-amine (10 mg, 0.0229 mmol), 2-methoxy-4-(methylsulfonyl)-N-(prop-2-yn-1-yl)aniline (10 mg, 0.0418 mmol, 1ClickChemistry), $Pd(PPh_3)_4$ (4 mg, 0.00346 mmol), and CuI (2 mg, 0.0105 mmol) were combined and dissolved in DMSO. The mixture was heated to 50 °C with strong stirring, as nitrogen was bubbled through the mixture. DIPEA (20 µL, 0.115 mmol) was added to the mixture and it was allowed to react overnight under a nitrogen atmosphere. After completion, the reaction was purified by reverse-phase HPLC (acetonitrile:water gradient up to 90%) to yield PMV6 as a light brown solid (3.1 mg, 0.00566 mmol, 24.7% yield). ^{1}H NMR (400 MHz, DMSO-d6) δ 7.39 (dd, J = 8.4, 2.0 Hz, 1H), 7.26 (d, J = 2.1 Hz, 1H), 7.09 (s, 1H), 7.00 (t, J = 8.0 Hz, 1H), 6.89 (d, J = 8.4 Hz, 1H), 6.68 (d, J = 8.2 Hz, 1H), 6.49 (t, J = 6.2 Hz, 1H), 6.16 (d, J = 7.8 Hz, 1H), 5.49 (d, J = 7.9 Hz, 1H), 4.93 (q, J = 9.0 Hz, 2H), 4.36 (d, J = 6.2 Hz, 2H), 3.90 (s, 3H), 3.10 (s, 3H), 2.79 (d, J = 11.2 Hz, 2H), 2.19 (s, 3H), 2.08-1.98 (m, 2H), 1.92 (d, J = 12.6 Hz, 2H), 1.55-1.42 (m, 2H), 1.28-1.11 (m, 1H). HRMS (top: observed, bottom: theoretical isotope pattern; M+H, $C_{27}H_{31}F_3N_4O_3S$+H] m/z theoretical 549.2142, found 549.2138.

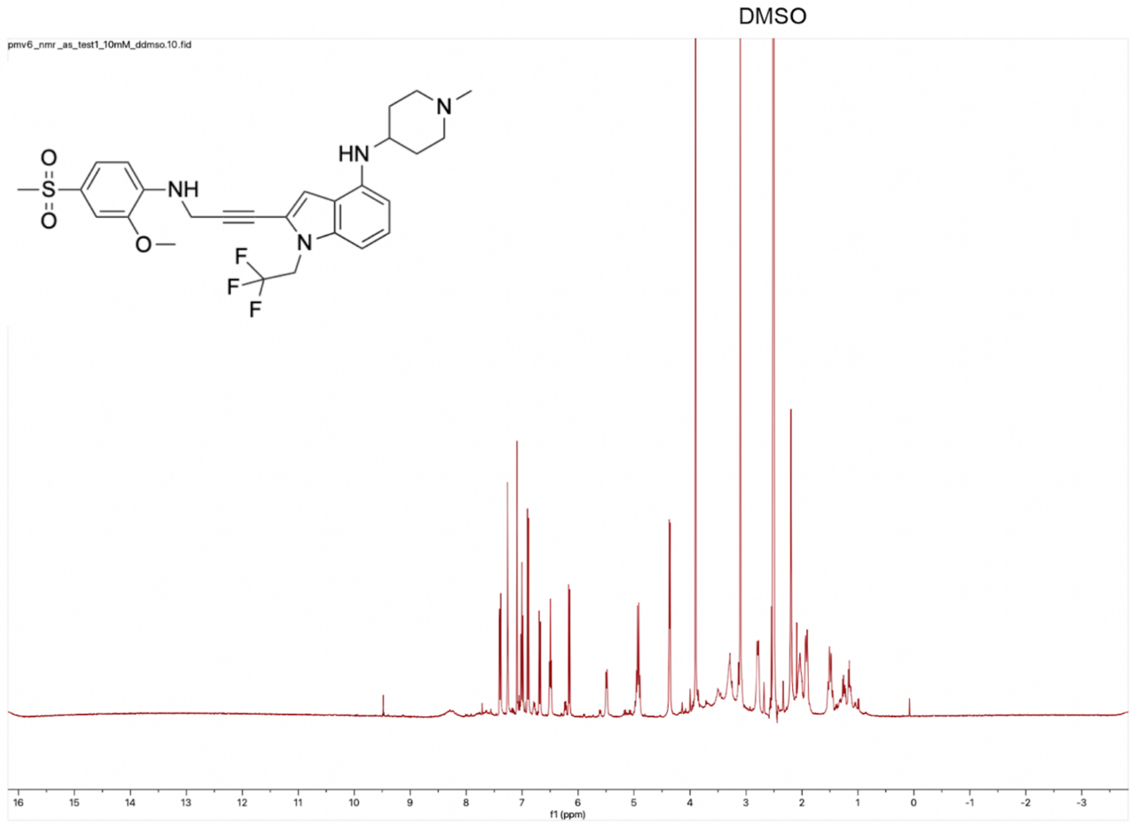
DMSO
pmv6_nmr_as_test1_10mM_ddmso.10.fid
f1 (ppm)

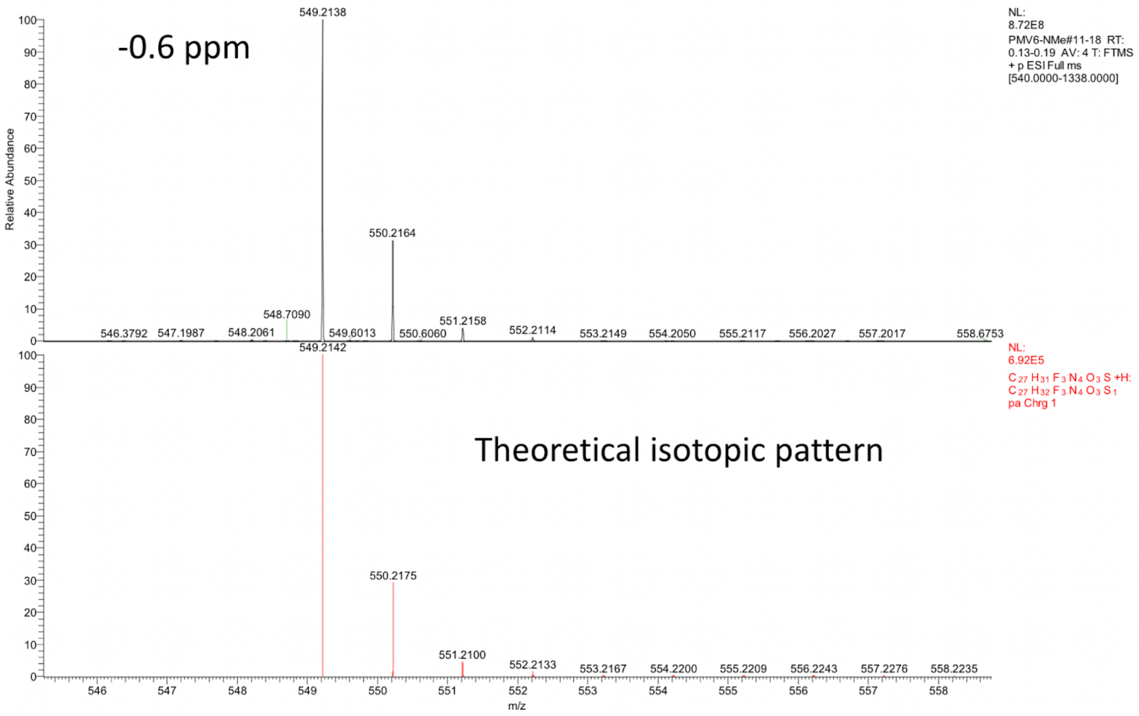
-0.6 ppm
NL: 8.72E8
PMV6-NMe#11-18 RT: 0.13-0.19 AV: 4 T: FTMS + p ESI Full ms [540.0000-1338.0000]
549.2138
550.2164
551.2158
NL: 6.92E5
C 27 H 31 F 3 N 4 O 3 S +H: C 27 H 32 F 3 N 4 O 3 S 1 pa Chrg 1
549.2142
550.2175
551.2100
Theoretical isotopic pattern
Relative Abundance
m/z

PMV6-NBoc.

16 **19** **18** **20**

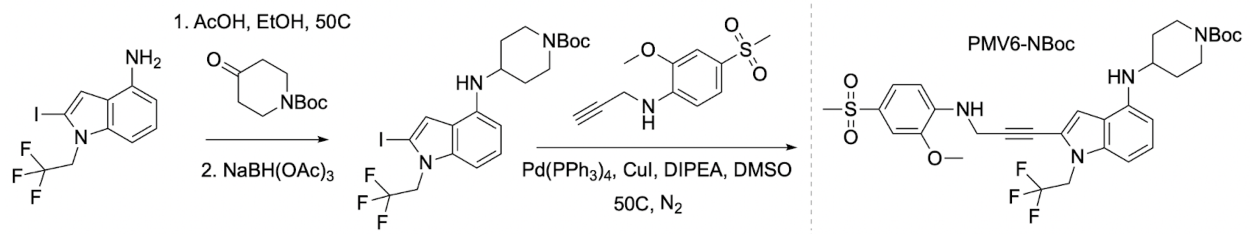

tert-butyl-4-((2-(3-((2-methoxy-4-(methylsulfonyl)phenyl)amino)prop-1-yn-1-yl)-1-(2,2,2-trifluoroethyl)-1H-indol-4-yl)amino)piperidine-1-carboxylate (PMV6-NBoc). To a solution of 2-iodo-1-(2,2,2-trifluoroethyl)-1H-indol-4-amine (45 mg, 0.133 mmol, eNovation Chemicals) in ethanol (5 mL), 1-Boc-4-piperidone (95 µL, 0.537 mmol) and acetic acid (50 µL, 0.874 mmol) were added, and the mixture was heated to 50 °C with strong stirring. After 30 minutes, sodium triacetoxyborohydride (108 mg, 0.510 mmol) was added and allowed to react overnight. After completion, the reaction was purified by reverse-phase HPLC (acetonitrile:water gradient up to 90%) to yield tert-butyl-4-((2-iodo-1-(2,2,2-trifluoroethyl)-1H-indol-4-yl)amino)piperidine-1-carboxylate as an off-white solid (18 mg, 0.0344 mmol, 25.9% yield). All of this product, 2-methoxy-4-(methylsulfonyl)-N-(prop-2-yn-1-yl)aniline (16.5 mg, 0.0690 mmol, 1ClickChemistry), $Pd(PPh_3)_4$ (4 mg, 0.00346 mmol), and CuI (2 mg, 0.0105 mmol) were combined and dissolved in DMSO. The mixture was heated to 50 °C with strong stirring, as nitrogen was bubbled through the mixture. DIPEA (36 µL, 0.207 mmol) was added to the mixture and it was allowed to react overnight under a nitrogen atmosphere. After completion, the reaction was purified by reverse-phase HPLC (acetonitrile:water gradient up to 90%) to yield PMV6-NBoc as a brown solid (9.8 mg, 0.0155 mmol, 45.0% yield). ^{1}H NMR (400 MHz, DMSO-d6) δ 7.39 (dd, *J* = 8.4, 2.0 Hz, 1H), 7.26 (d, *J* = 2.0 Hz, 1H), 7.06 (s, 1H), 7.01 (t, *J* = 8.0 Hz, 1H), 6.89 (d, *J* = 8.4 Hz, 1H), 6.70 (d, *J* = 8.3 Hz, 1H), 6.49 (t, *J* = 6.2 Hz, 1H), 6.22 (d, *J* = 7.8 Hz, 1H), 5.52 (d, *J* = 7.9 Hz, 1H), 4.93 (q, *J* = 9.1 Hz, 2H), 4.36 (d, *J* = 6.2 Hz, 2H), 3.90 (s, 3H), 3.10 (s, 3H), 2.96-2.80 (m, 2H), 1.93 (d, *J* = 11.6 Hz, 2H), 1.41 (s, 9H), 1.39-1.22 (m, 4H), 0.91-0.79 (m, 1H). HRMS (top: observed, bottom: theoretical isotope pattern; M+H, $C_{31}H_{37}F_3N_4O_5S$+H] m/z theoretical 635.2510, found 635.2499.

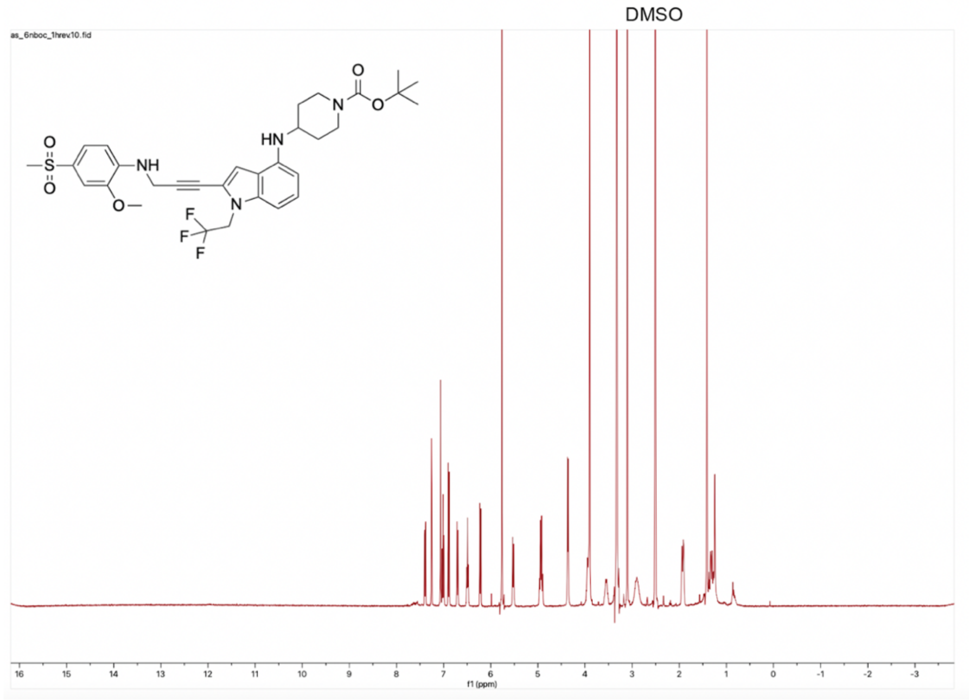

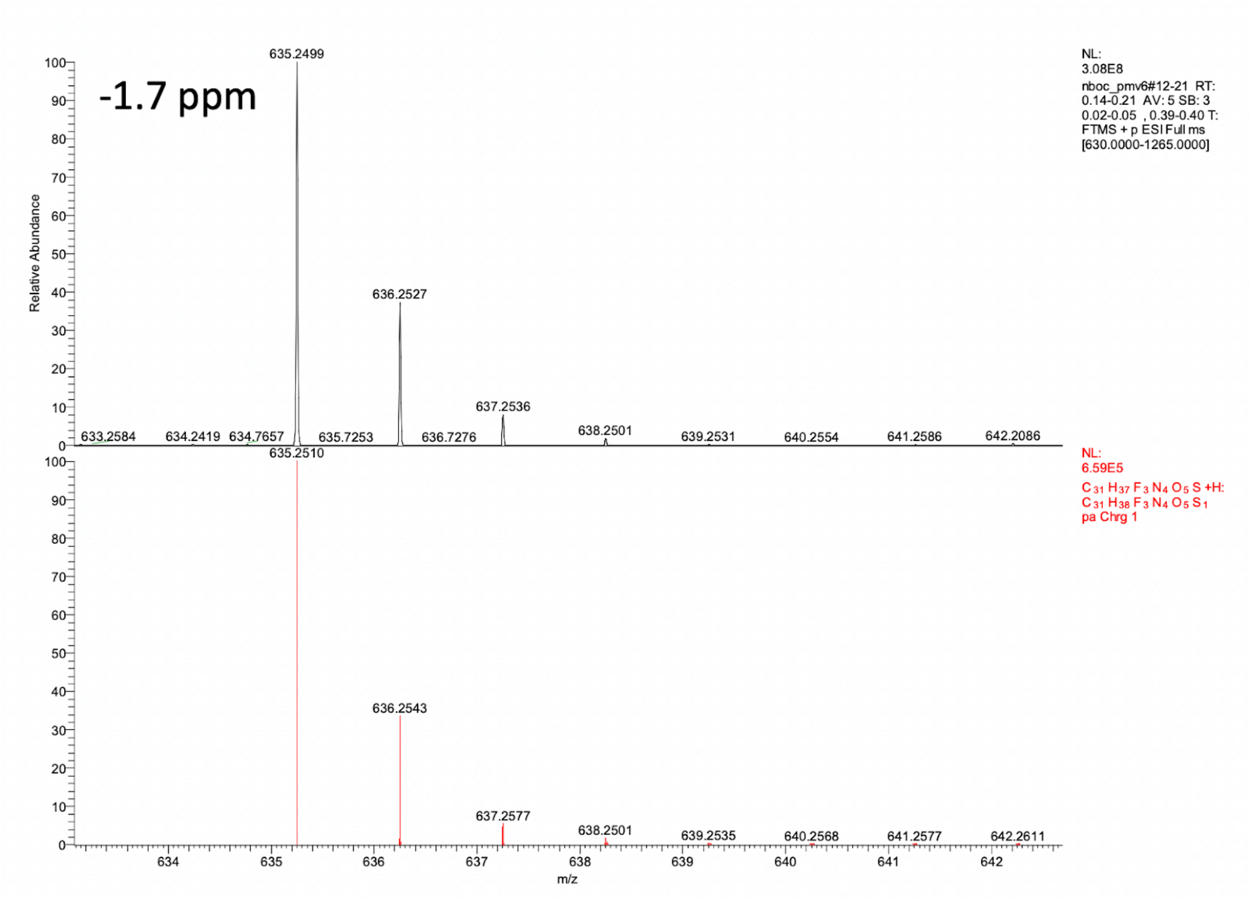

PMV6-PEG4-BI2536 (p53-01).

20 **5**

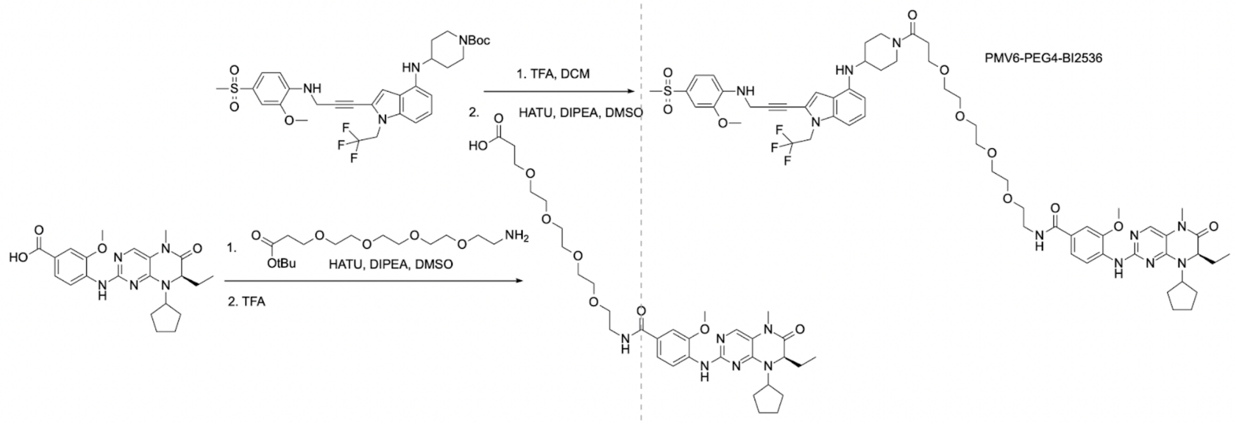

21 **22**

(R)-4-((8-cyclopentyl-7-ethyl-5-methyl-6-oxo-5,6,7,8-tetrahydropteridin-2-yl)amino)-3-methoxy-N-(15-(4-((2-(3-((2-methoxy-4-(methylsulfonyl)phenyl)amino)prop-1-yn-1-yl)-1-(2,2,2-trifluoroethyl)-1H-indol-4-yl)amino)piperidin-1-yl)-15-oxo-3,6,9,12-tetraoxapentadecyl)benzamide (PMV6-PEG4-

BI2536, PMV6-PEG4-BI, p53-01). To a solution of (R)-4-(8-cyclopentyl-7-ethyl-5-methyl-6-oxo-5,6,7,8-tetrahydropteridin-2-ylamino)-3-methoxybenzoic acid (10 mg, 0.0235 mmol, 1ClickChemistry) in DMSO (1 mL), H_2N-PEG4-$CH_2CH_2CO_2$tBu (20 µL, 0.0755 mmol), HATU (12 mg, 0.0315 mmol), and DIPEA (20 µL, 0.115 mmol) were added. After stirring for 15 minutes, trifluoroacetic acid (2 mL) was added and the reaction was stirred for 1 hour. After completion, the reaction was purified by reverse-phase HPLC (acetonitrile:water gradient up to 90%) to yield (R)-1-(4-((8-cyclopentyl-7-ethyl-5-methyl-6-oxo-5,6,7,8-tetrahydropteridin-2-yl)amino)-3-methoxyphenyl)-1-oxo-5,8,11,14-tetraoxa-2-azaheptadecan-17-oic acid (BI2536-PEG4-acid) as a white solid (10 mg, 0.0149 mmol, 63.1% yield). tert-butyl-4-((2-(3-((2-methoxy-4-(methylsulfonyl)phenyl)amino)prop-1-yn-1-yl)-1-(2,2,2-trifluoroethyl)-1H-indol-4-yl)amino)piperidine-1-carboxylate (PMV6-NBoc, 8.2 mg, 0.0129 mmol) was dissolved in DCM (1 mL), and trifluoroacetic acid (2 mL) was added to the solution. After stirring for 1 hour, solvents were removed under reduced pressure. The residue was redissolved in DMSO (1 mL) and BI2536-PEG4-acid (10 mg, 0.0149 mmol), HATU (6 mg, 0.0158 mmol), and DIPEA (20 µL, 0.115 mmol) were added to the solution. After completion (30 minutes), the reaction was purified by reverse-phase HPLC (acetonitrile:water gradient up to 90%) to yield PMV6-PEG4-BI2536 as a brown solid (5.6 mg, 0.00471 mmol, 36.5% yield). ^{1}H NMR (400 MHz, DMSO-d6) δ 8.47 – 8.37 (m, 2H), 7.85 (s, 1H), 7.61 (s, 1H), 7.50 (d, *J* = 7.3 Hz, 2H), 7.39 (dd, *J* = 8.3, 2.0 Hz, 1H), 7.26 (d, *J* = 2.0 Hz, 1H), 7.07 (s, 1H), 7.01 (t, *J* = 8.0 Hz, 1H), 6.89 (d, *J* = 8.4 Hz, 1H), 6.71 (d, *J* = 8.2 Hz, 1H), 6.49 (t, *J* = 6.3 Hz, 1H), 6.22 (d, *J* = 7.9 Hz, 1H), 5.53 (d, *J* = 8.0 Hz, 1H), 4.93 (q, *J* = 9.1 Hz, 2H), 4.39 – 4.27 (m, 4H), 4.24 (dd, *J* = 7.6, 3.6 Hz, 1H), 3.91 (d, *J* = 14.4 Hz, 6H), 3.87 (s, 1H), 3.61 (t, *J* = 6.7 Hz, 2H), 3.56 – 3.50 (m, 6H), 3.49 (d, *J* = 3.1 Hz, 7H), 3.44 (dd, *J* = 12.6, 7.0 Hz, 2H), 3.25 (s, 3H), 3.18 (s, 3H), 3.14 (s, 1H), 3.10 (s, 3H), 2.76 (t, *J* = 12.0 Hz, 1H), 2.57 (t, *J* = 6.7 Hz, 2H), 2.07 – 1.84 (m, 5H), 1.83 – 1.71 (m, 3H), 1.70 – 1.55 (m, 3H), 1.42 – 1.22 (m, 2H), 0.76 (t, *J* = 7.4 Hz, 3H). ^{13}C NMR (101 MHz, DMSO-d6) δ 168.96, 166.29, 163.40, 154.79, 151.96, 147.15, 146.58, 141.83, 140.99, 138.80, 138.30, 132.68, 129.04 – 124.35 (m), 123.28 – 114.61 (m), 109.63, 109.10, 108.23, 107.61, 100.02, 99.10, 93.63, 73.76, 71.79 – 68.74 (m), 69.59, 67.35, 60.28, 58.85, 56.36 (d, *J* = 20.4 Hz), 49.23 (d, *J* = 32.7 Hz), 44.63 (d, *J* = 33.4 Hz), 33.29, 32.94, 32.68, 29.19, 28.87, 28.25, 26.95, 23.64, 23.39, 9.32. HRMS (top: observed, bottom: theoretical isotope pattern; M+H, $C_{59}H_{75}F_3N_{10}O_{11}S$+H] m/z theoretical 1189.5362, found 1189.5350.

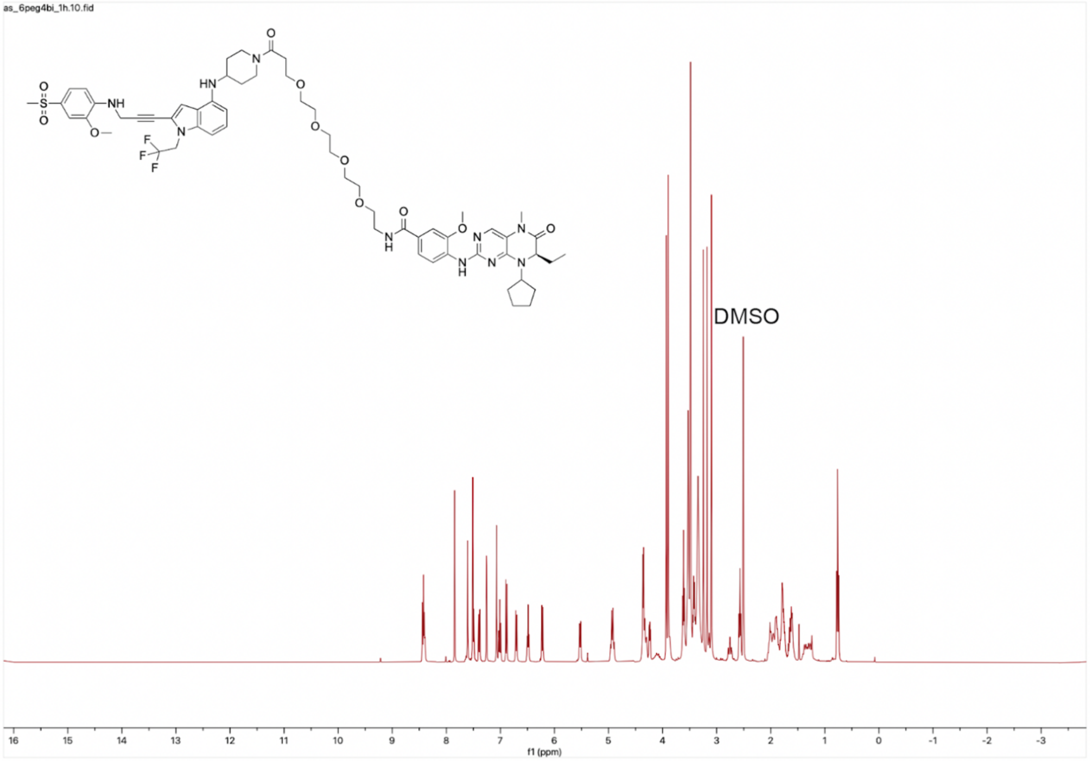

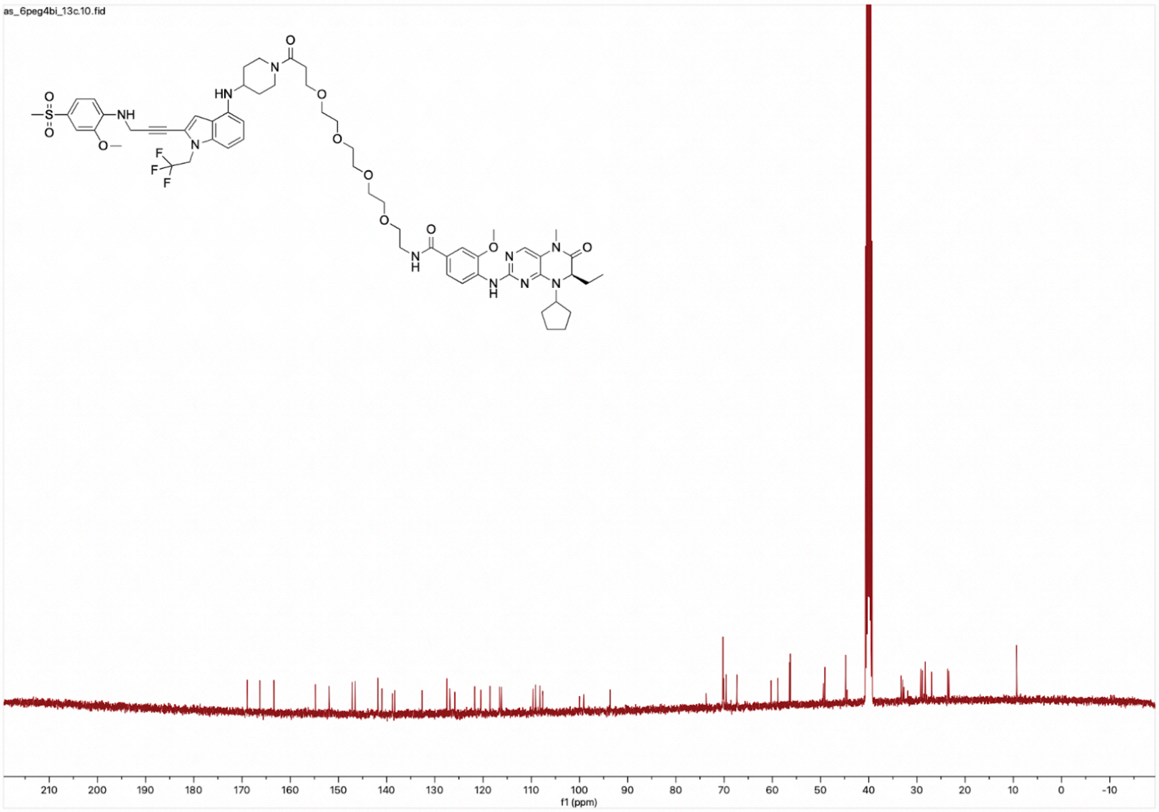
as_6peg4bi_13c.10.fid
210 200 190 180 170 160 150 140 130 120 110 100 90 80 70 60 50 40 30 20 10 0 -10
f1 (ppm)

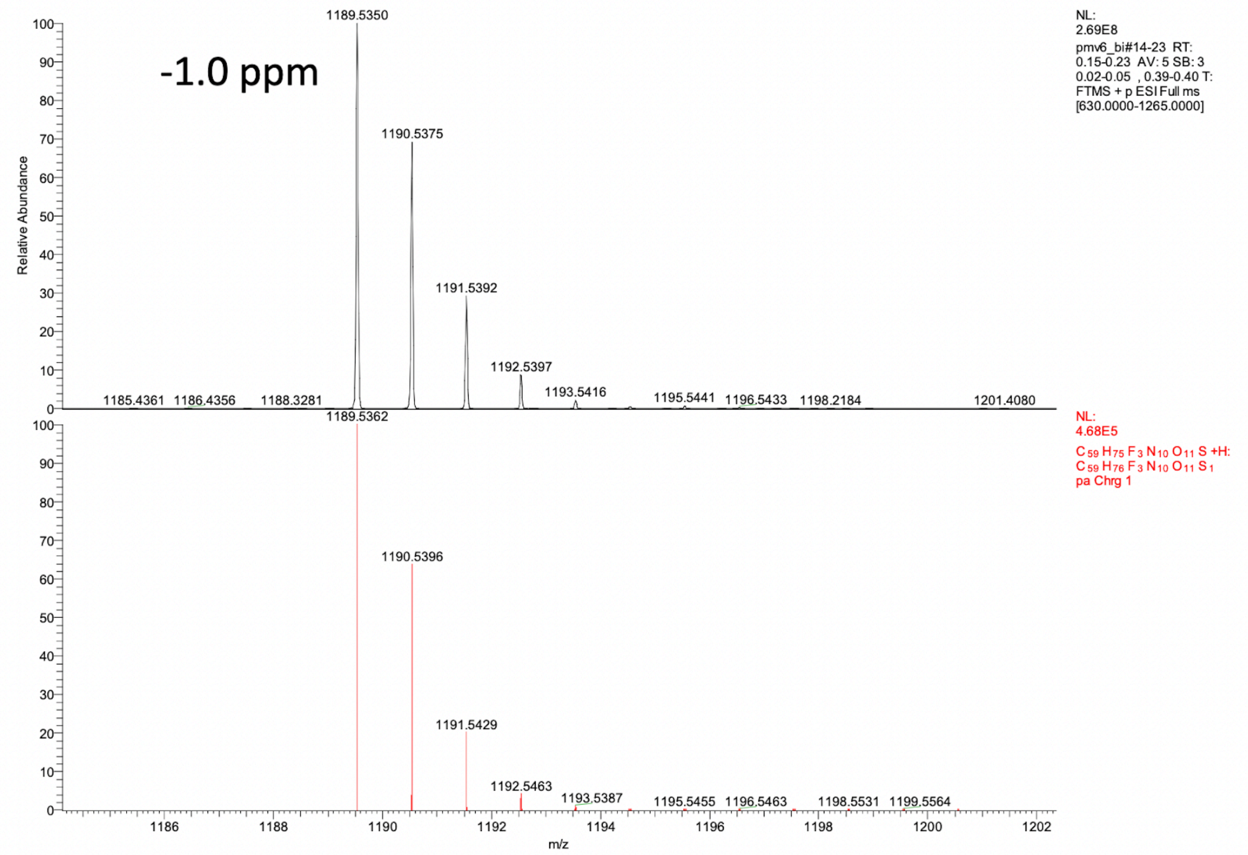
-1.0 ppm
1189.5350
1190.5375
1191.5392
1192.5397
1193.5416
1185.4361 1186.4356 1188.3281
1195.5441 1196.5433 1198.2184 1201.4080
NL:
2.69E8
pmv6_bi#14-23 RT:
0.15-0.23 AV: 5 SB: 3
0.02-0.05 , 0.39-0.40 T:
FTMS + p ESI Full ms
[630.0000-1265.0000]
Relative Abundance
1189.5362
1190.5396
1191.5429
1192.5463
1193.5387
1195.5455 1196.5463 1198.5531 1199.5564
NL:
4.68E5
C59 H75 F3 N10 O11 S +H:
C59 H76 F3 N10 O11 S1
pa Chrg 1
m/z

Other PMV6-BI2536 bifunctionals.

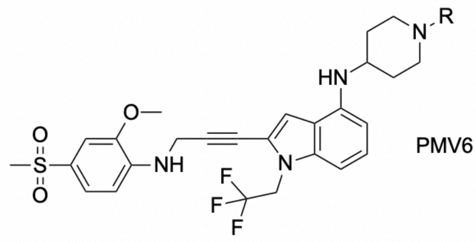

PMV6

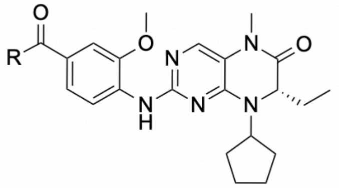

BI2536

8

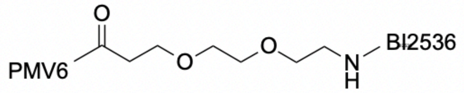

PEG2

9

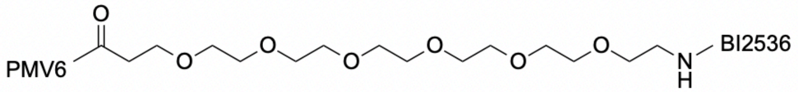

PEG6

10

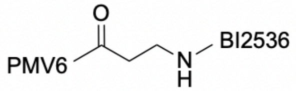

C3

11

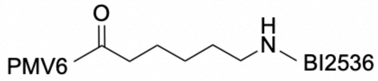

C6

12

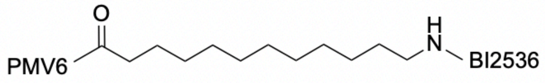

C12

13

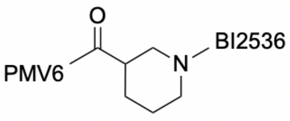

Nipecotic acid

14

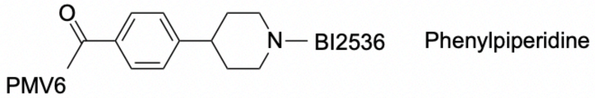

15

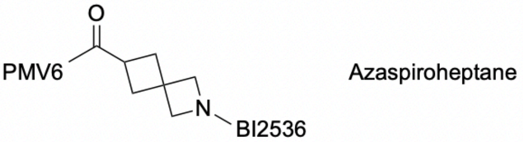

The general procedure for the synthesis of PEG2, C6, and phenylpiperidine compounds was based on the procedure for the synthesis of PMV6-PEG4-BI2536. In brief, the linker (PEG2: amino-PEG2-t-butyl ester, C6: t-butyl-6-aminohexanoate, phenylpiperidine: methyl 4-(piperidin-4-yl)benzoate) was coupled with a stoichiometric amount of (R)-4-(8-cyclopentyl-7-ethyl-5-methyl-6-oxo-5,6,7,8-tetrahydropteridin-2-ylamino)-3-methoxybenzoic acid in the presence of excess HATU (~2 equivalents) and triethylamine (~5 equivalents) in DMSO (1 mL). Subsequently, TFA (3 mL; for PEG2 and C6 linker) or 4M LiOH/H_2O solution (3 mL, phenylpiperidine linker) were added and the solutions were stirred for 4 hours at room temperature. The reactions were purified by reverse-phase HPLC (acetonitrile:water gradient up to 90%) and the resulting compounds were coupled with stoichiometric hydrolyzed PMV6-NBoc (75% TFA in DCM for 1 hour, solvents evaporated), in the presence of excess HATU (~2 equivalents) and triethylamine (~20 equivalents). The reactions were purified by reverse-phase HPLC (acetonitrile:water gradient up to 90%) to yield the products as yellow solids. For the synthesis of azaspiroheptane compound 2-(tert-Butoxycarbonyl)-2-azaspiro[3.3]heptane-6-carboxylic acid was used as the linker. The first coupling occurred with hydrolyzed PMV6-NBoc and the second coupling occurred with (R)-4-(8-cyclopentyl-7-ethyl-5-methyl-6-oxo-5,6,7,8-tetrahydropteridin-2-ylamino)-3-methoxybenzoic acid to yield the product as a yellow solid. For the synthesis of the remaining compounds (PEG6: amino-PEG6-acid, C12: 12-aminododecanoic acid, C3: beta-alanine, nipecotic acid: nipecotic acid), the reaction occurred in one-pot; with stoichiometric hydrolyzed PMV6-NBoc (1 equivalent), linker (1 equivalent), (R)-4-(8-cyclopentyl-7-ethyl-5-methyl-6-oxo-5,6,7,8-tetrahydropteridin-2-ylamino)-3-methoxybenzoic acid (1 equivalent), HATU (2 equivalents), and excess triethylamine (~20 equivalents). Purification by reverse-phase HPLC (acetonitrile:water gradient up to 90%) yielded the products as yellow solids. All reactions were performed at milligram scale, with 3 mg of PMV6-NBoc used per coupling. Overall yields across all steps were as follows: C12: 0.5 mg (9%), PEG2: 0.7 mg (13%), C6: 0.7 mg (14%), Spiro: 0.7 mg (14%), C3: 0.8 mg (17%), PEG6: 1.3 mg (22%), PhPip: 2.0 mg (38%), Nip: 2.4 mg (48%). Purified compounds were dissolved in CD_3CN and ^1H NMR was performed (400 MHz, CD_3CN). After NMRs were taken, solvent was evaporated under reduced pressure and the residue was redissolved in DMSO to create stock solutions. ^1H NMR (400 MHz, CD_3CN) and HRMS analysis (top: observed, bottom: theoretical isotope pattern) is provided for all compounds.

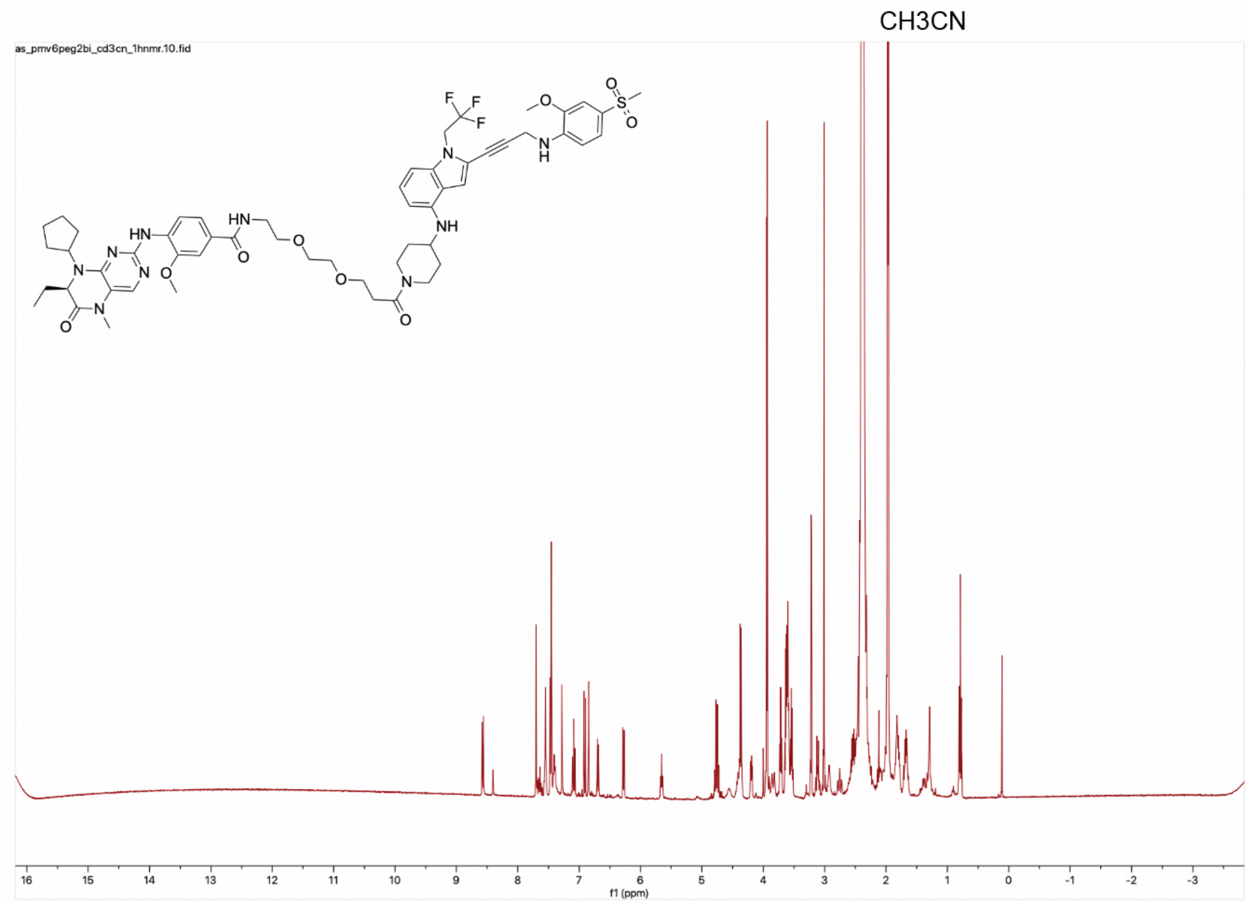
CH3CN
as_pmv6peg2bi_cd3cn_1hnmr.10.fid
f1 (ppm)

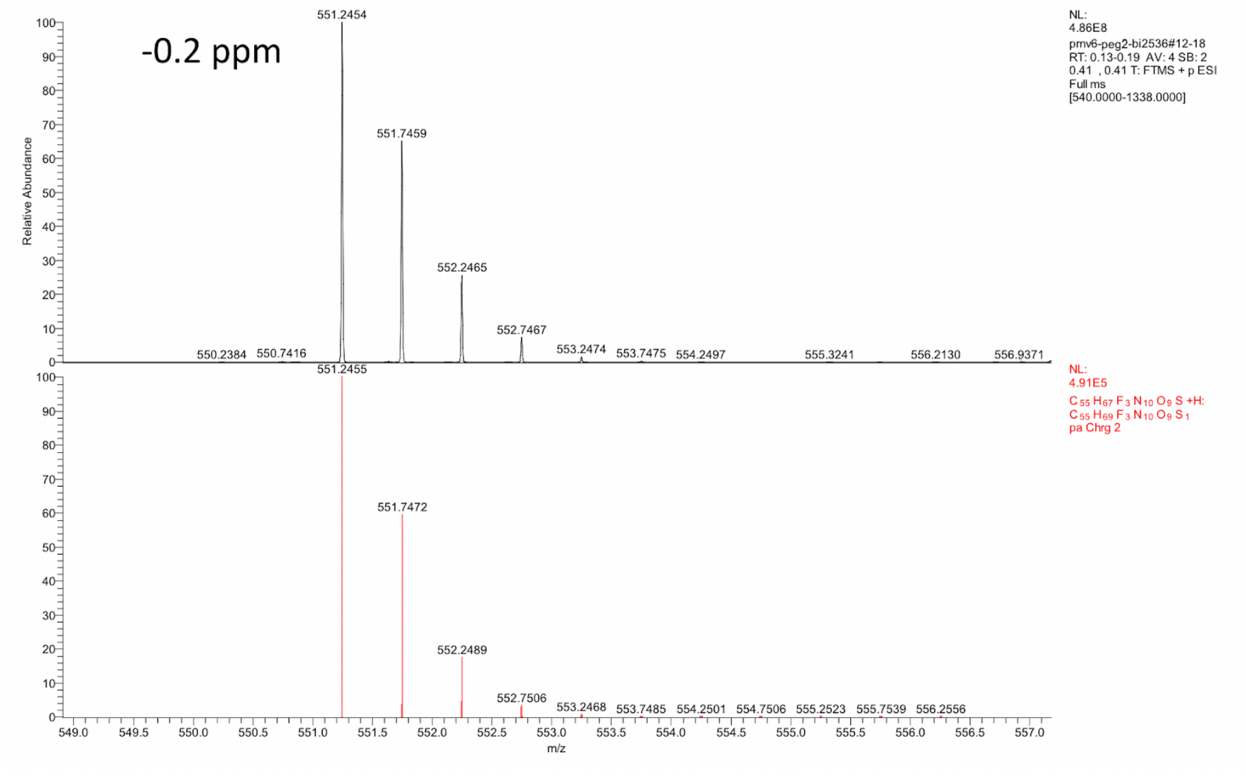
-0.2 ppm
Relative Abundance
551.2454
551.7459
552.2465
552.7467
553.2474
553.7475
554.2497
555.3241
556.2130
556.9371
550.2384
550.7416
NL:
4.86E8
pmv6-peg2-bi2536#12-18
RT: 0.13-0.19 AV: 4 SB: 2
0.41 , 0.41 T: FTMS + p ESI
Full ms
[540.0000-1338.0000]
551.2455
551.7472
552.2489
552.7506
553.2468
553.7485
554.2501
554.7506
555.2523
555.7539
556.2556
NL:
4.91E5
C 55 H67 F 3 N 10 O9 S +H:
C 55 H69 F 3 N 10 O9 S 1
pa Chrg 2
m/z

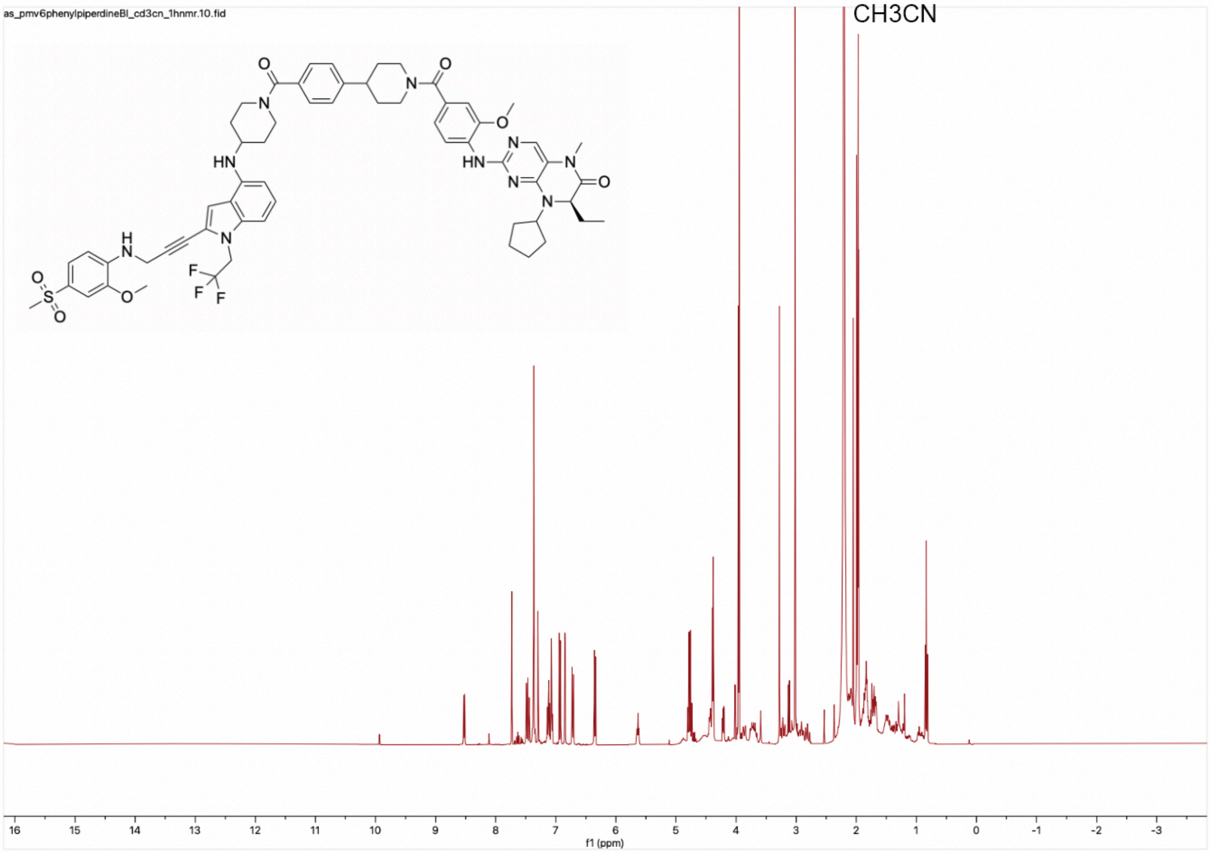
as_pmv6phenylpiperdineBI_cd3cn_1hnmr.10.fid
CH3CN
f1 (ppm)

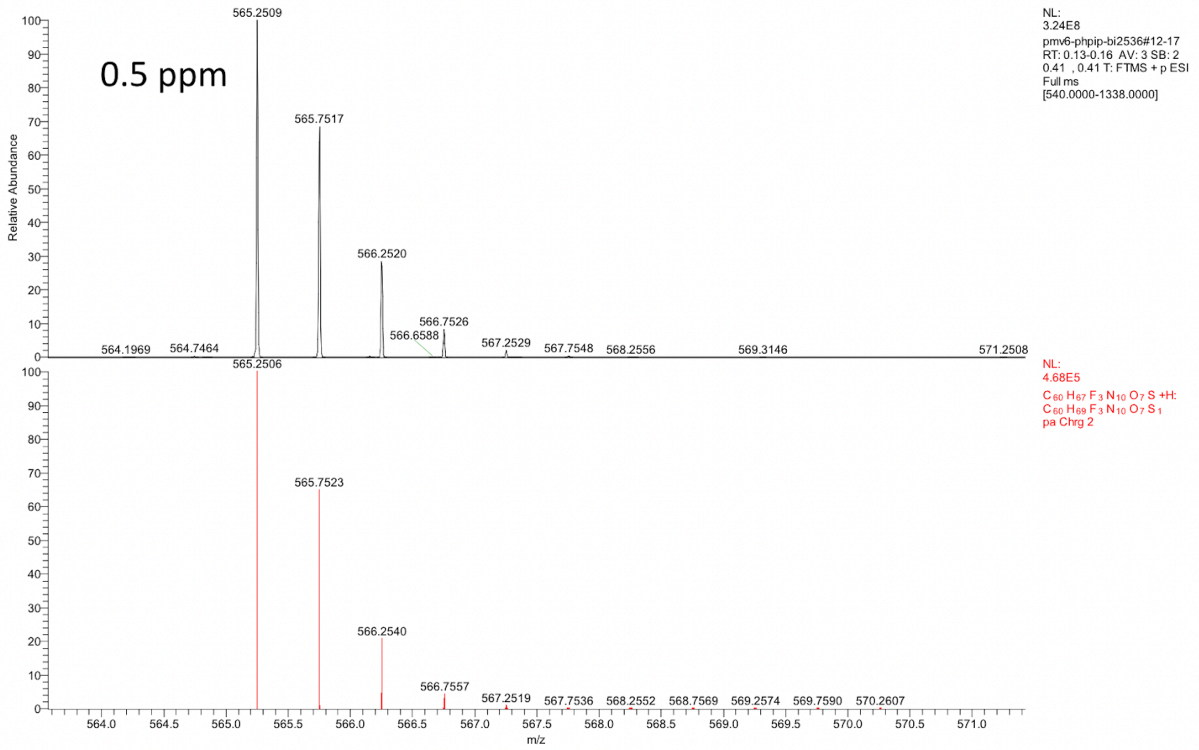
0.5 ppm
NL:
3.24E8
pmv6-phpip-bi2536#12-17
RT: 0.13-0.16 AV: 3 SB: 2
0.41 , 0.41 T: FTMS + p ESI
Full ms
[540.0000-1338.0000]
565.2509
565.7517
566.2520
566.7526
566.6588
567.2529
567.7548
568.2556
569.3146
571.2508
564.1969
564.7464
Relative Abundance
NL:
4.68E5
C60 H67 F3 N10 O7 S +H:
C60 H69 F3 N10 O7 S1
pa Chrg 2
565.2506
565.7523
566.2540
566.7557
567.2519
567.7536
568.2552
568.7569
569.2574
569.7590
570.2607
m/z

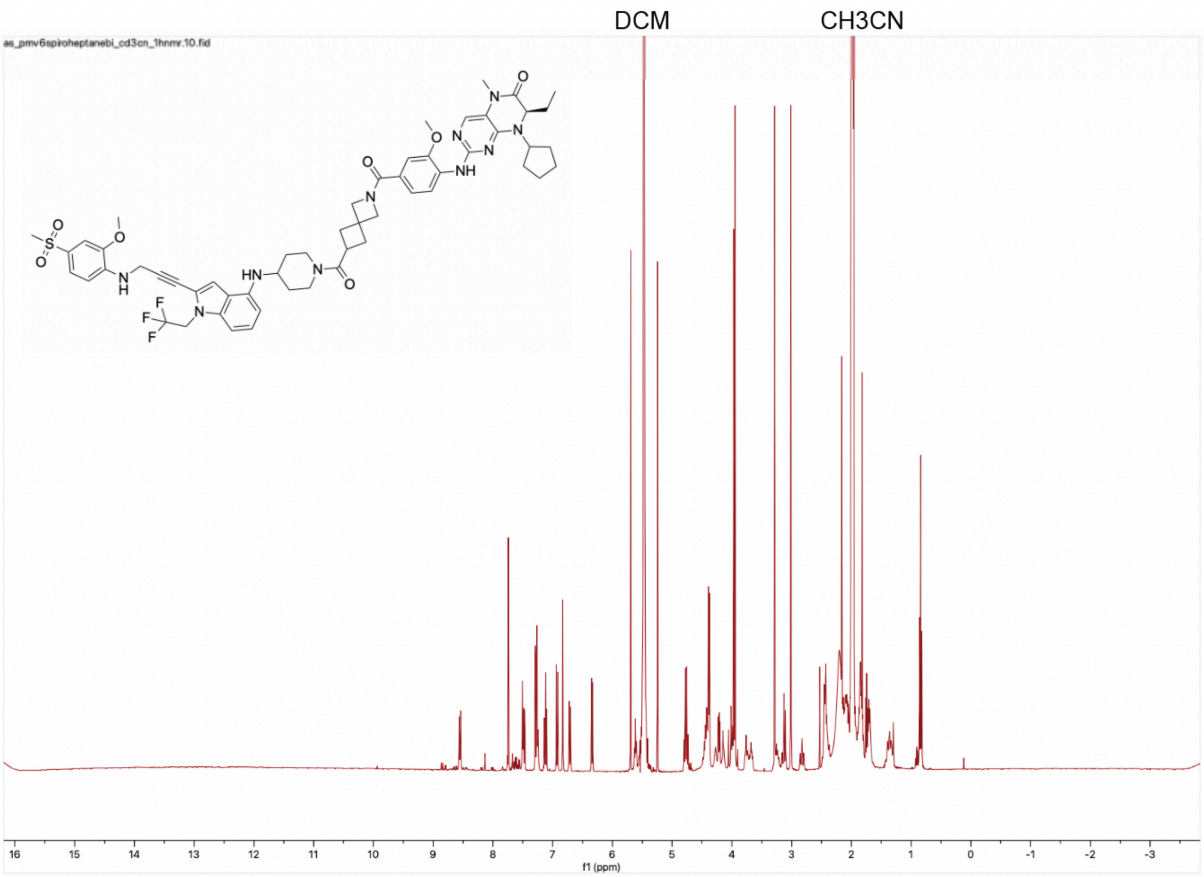
DCM
CH3CN
as_pmv6spiroheptanebi_cd3cn_1hnmr.10.fid
f1 (ppm)

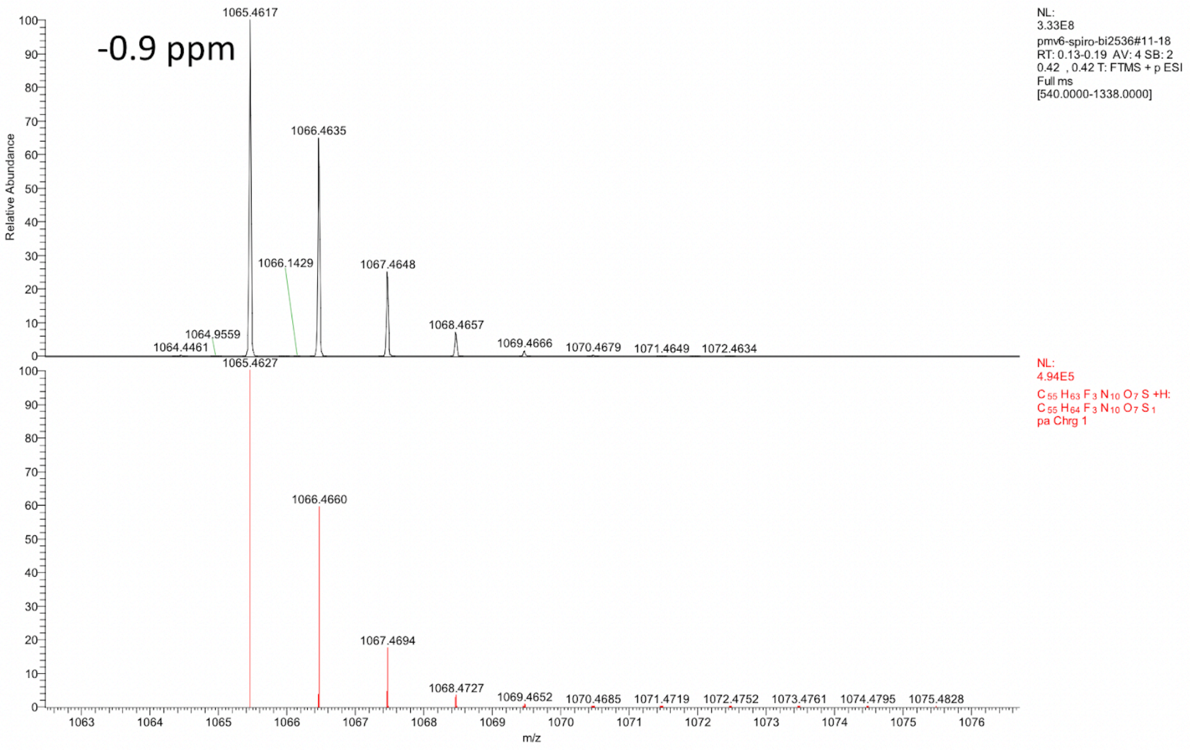
-0.9 ppm
1065.4617
1066.4635
1066.1429
1067.4648
1068.4657
1064.9559
1064.4461
1069.4666
1070.4679
1071.4649
1072.4634
NL:
3.33E8
pmv6-spiro-bi2536#11-18
RT: 0.13-0.19 AV: 4 SB: 2
0.42 , 0.42 T: FTMS + p ESI
Full ms
[540.0000-1338.0000]
1065.4627
1066.4660
1067.4694
1068.4727
1069.4652
1070.4685
1071.4719
1072.4752
1073.4761
1074.4795
1075.4828
NL:
4.94E5
C 55 H 63 F 3 N 10 O 7 S +H:
C 55 H 64 F 3 N 10 O 7 S 1
pa Chrg 1
Relative Abundance
m/z

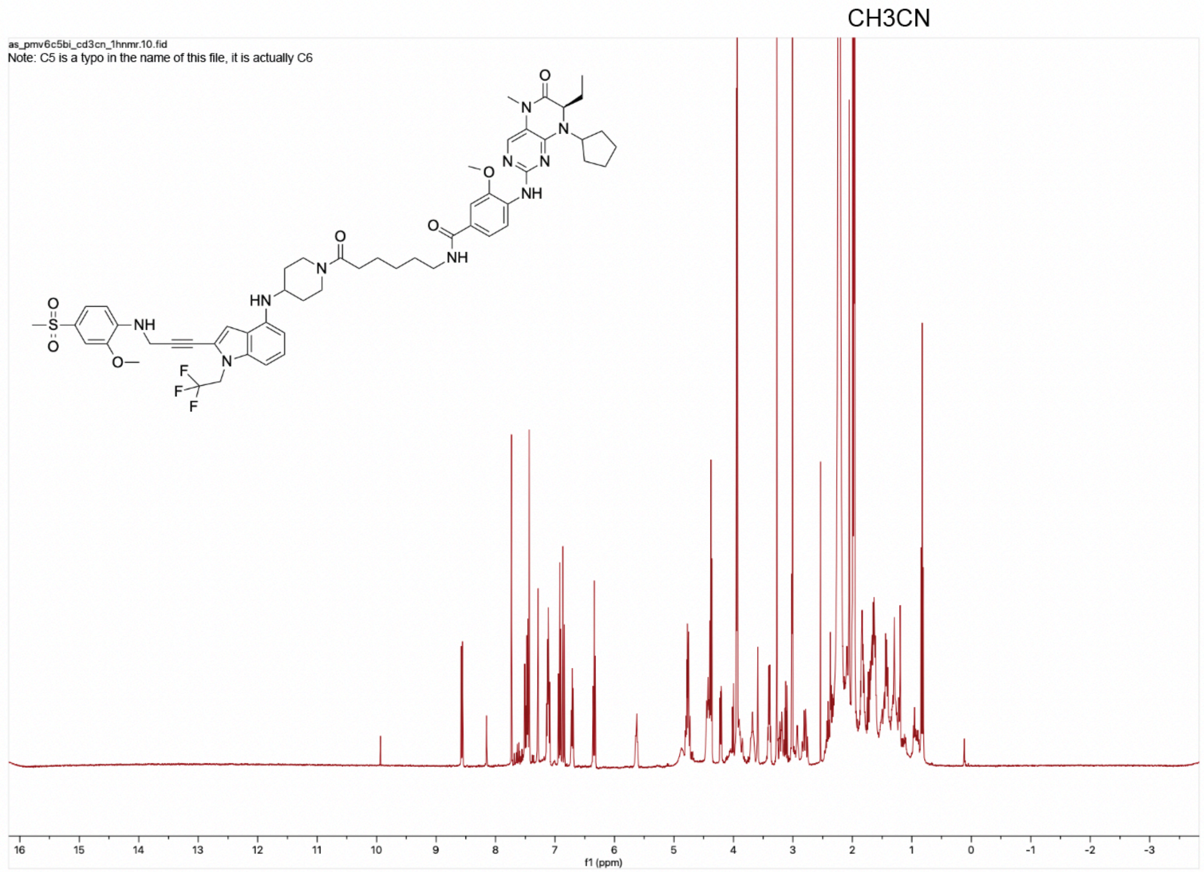
CH3CN
as_pmv6c5bi_cd3cn_1hnmr.10.fid
Note: C5 is a typo in the name of this file, it is actually C6
f1 (ppm)

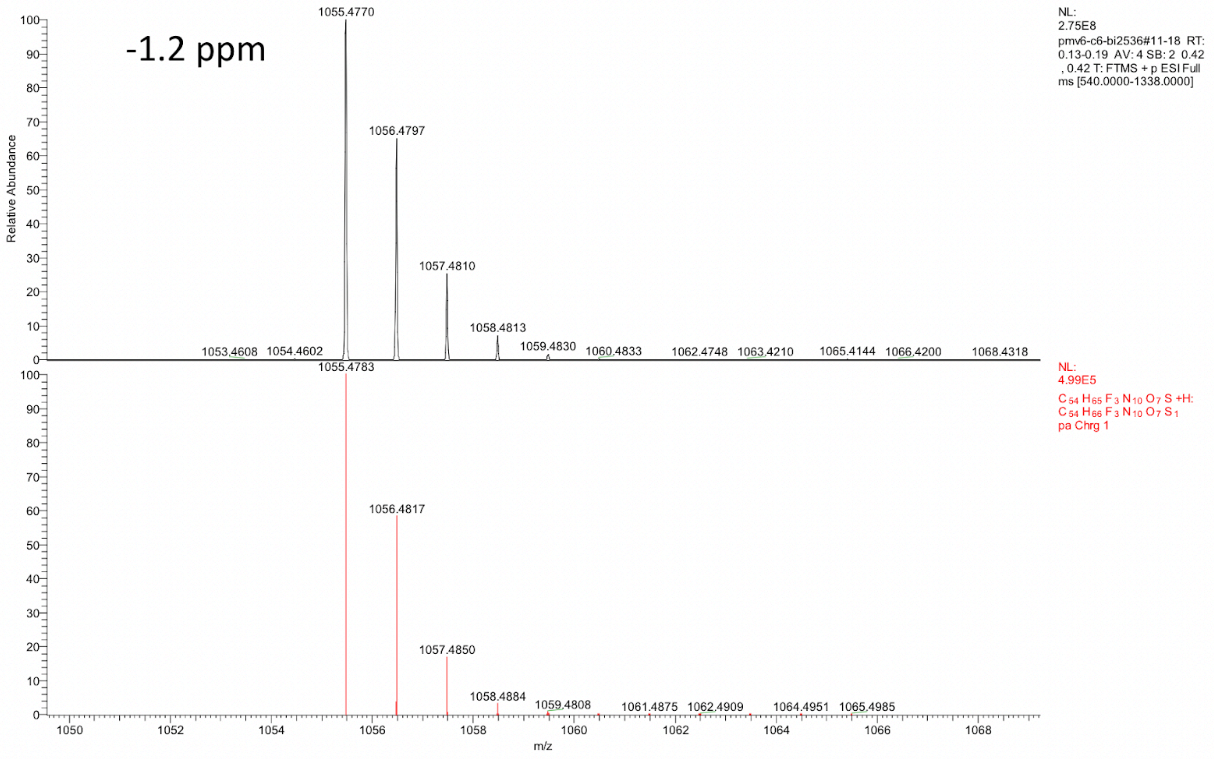
-1.2 ppm
NL:
2.75E8
pmv6-c6-bi2536#11-18 RT:
0.13-0.19 AV: 4 SB: 2 0.42
, 0.42 T: FTMS + p ESI Full
ms [540.0000-1338.0000]
1055.4770
1056.4797
1057.4810
1058.4813
1059.4830
1053.4608
1054.4602
1060.4833
1062.4748
1063.4210
1065.4144
1066.4200
1068.4318
NL:
4.99E5
C 54 H 65 F 3 N 10 O 7 S +H:
C 54 H 66 F 3 N 10 O 7 S 1
pa Chrg 1
1055.4783
1056.4817
1057.4850
1058.4884
1059.4808
1061.4875
1062.4909
1064.4951
1065.4985
Relative Abundance
m/z

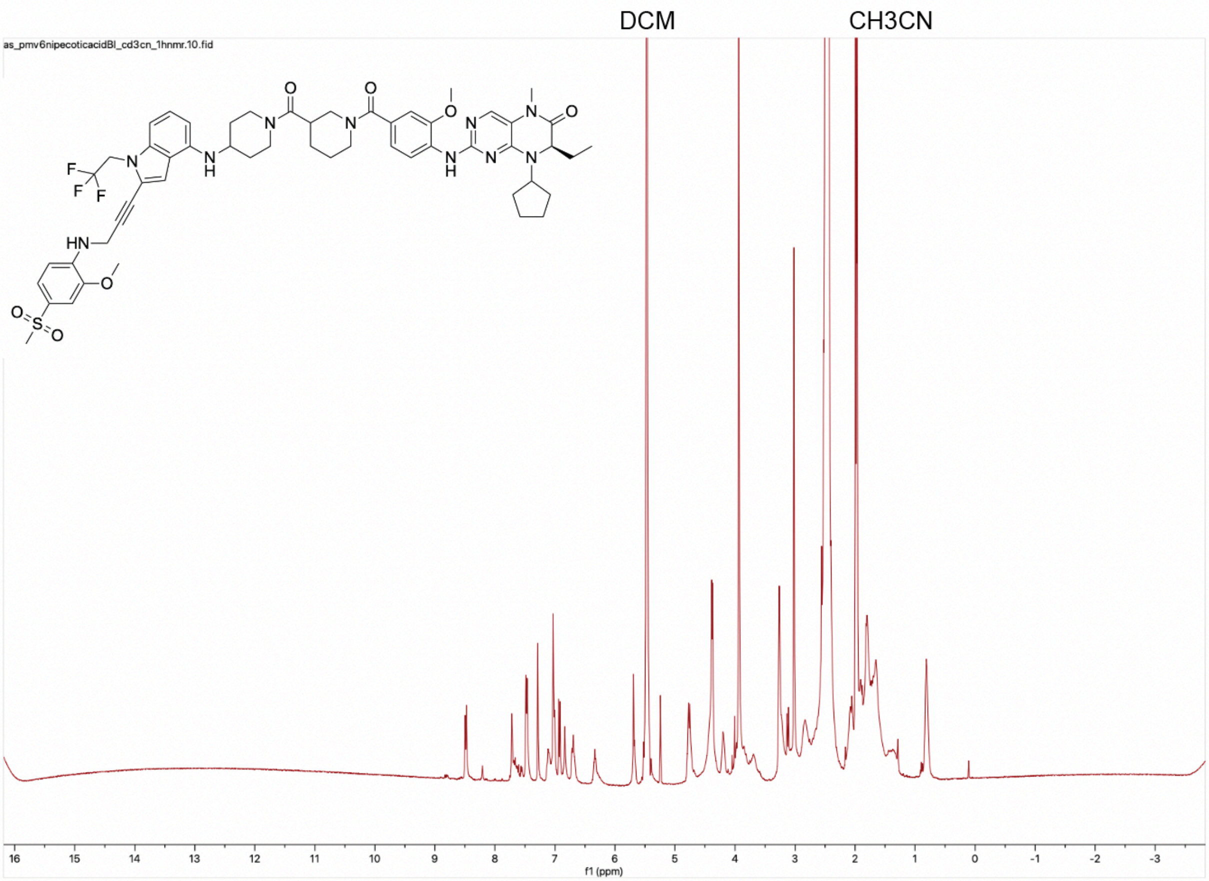
DCM
CH3CN
as_pmv6nipecoticacidBI_cd3cn_1hnmr.10.fid
f1 (ppm)

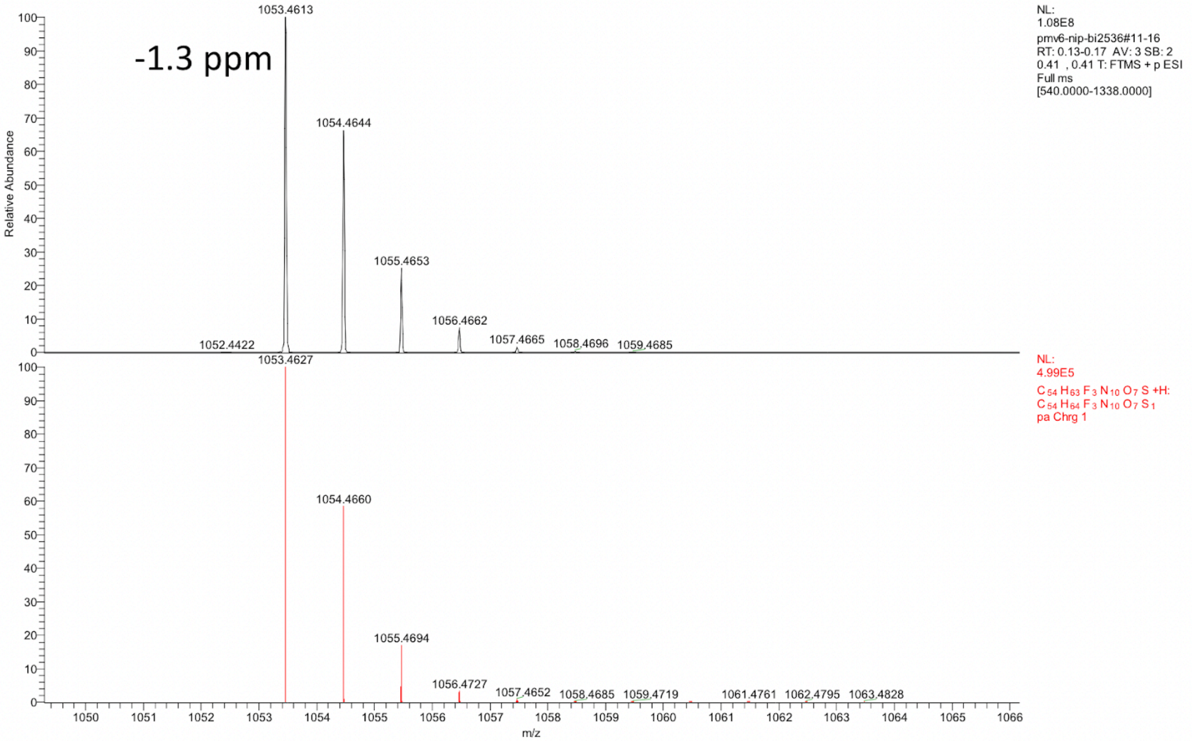
-1.3 ppm
NL:
1.08E8
pmv6-nip-bi2536#11-16
RT: 0.13-0.17 AV: 3 SB: 2
0.41 , 0.41 T: FTMS + p ESI
Full ms
[540.0000-1338.0000]
NL:
4.99E5
C54 H63 F3 N10 O7 S +H:
C54 H64 F3 N10 O7 S1
pa Chrg 1
Relative Abundance
m/z
1053.4613
1054.4644
1055.4653
1056.4662
1057.4665
1058.4696
1059.4685
1052.4422
1053.4627
1054.4660
1055.4694
1056.4727
1057.4652
1058.4685
1059.4719
1061.4761
1062.4795
1063.4828

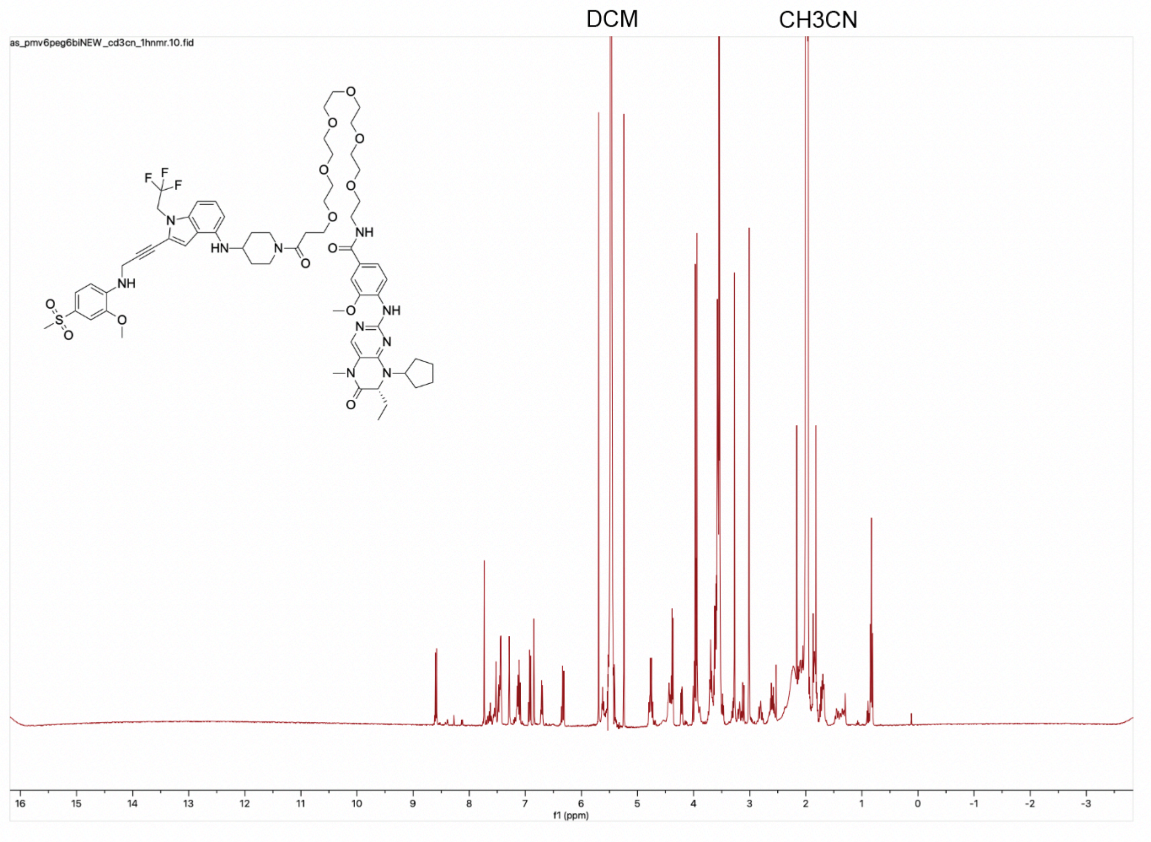
DCM
CH3CN
as_pmv6peg6biNEW_cd3cn_1hnmr.10.fid
f1 (ppm)

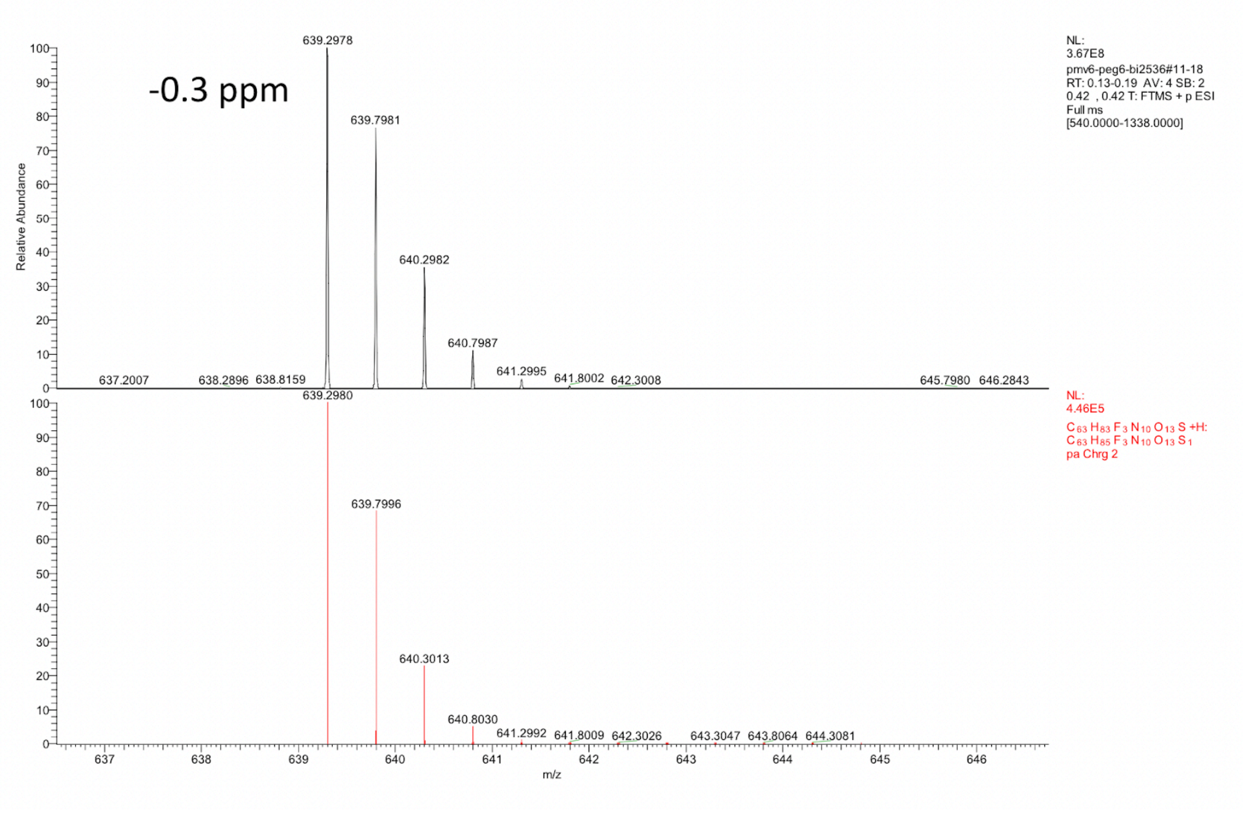
-0.3 ppm
NL:
3.67E8
pmv6-peg6-bi2536#11-18
RT: 0.13-0.19 AV: 4 SB: 2
0.42 , 0.42 T: FTMS + p ESI
Full ms
[540.0000-1338.0000]
Relative Abundance
639.2978
639.7981
640.2982
640.7987
641.2995
641.8002
642.3008
637.2007
638.2896
638.8159
645.7980
646.2843
NL:
4.46E5
C63 H83 F3 N10 O13 S +H:
C63 H85 F3 N10 O13 S1
pa Chrg 2
639.2980
639.7996
640.3013
640.8030
641.2992
641.8009
642.3026
643.3047
643.8064
644.3081
m/z

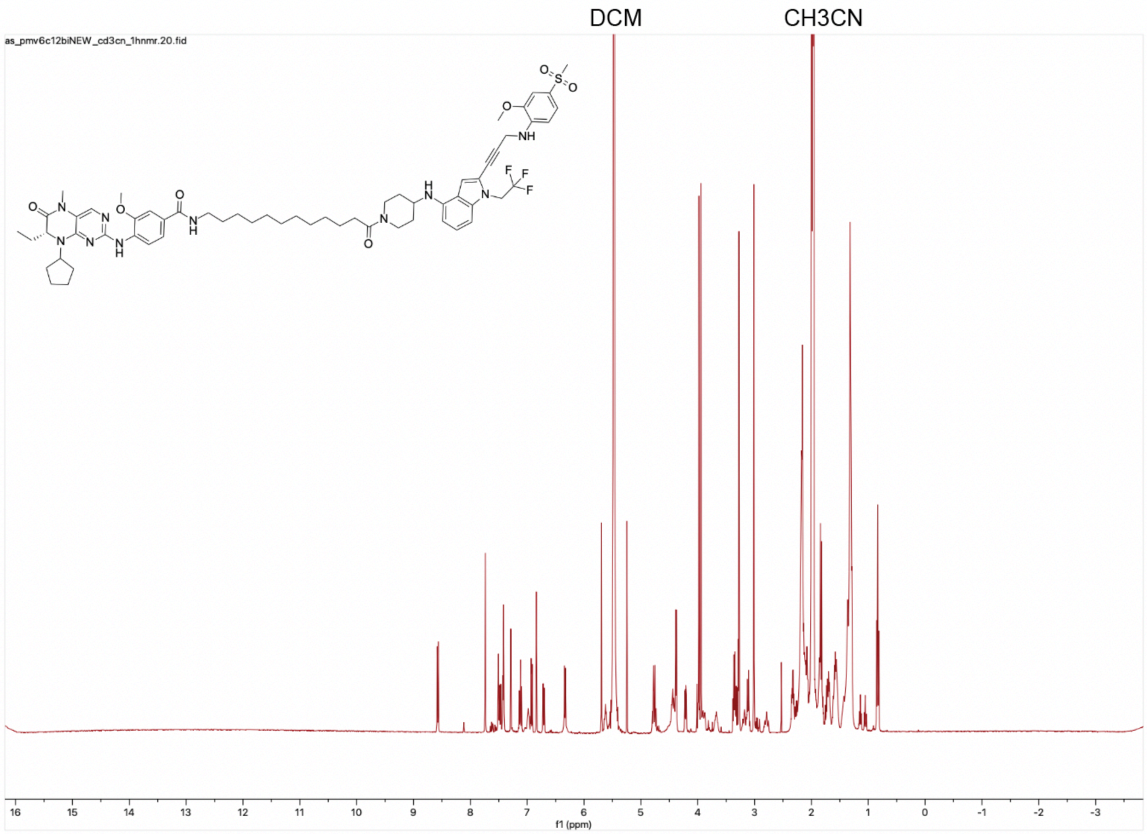
DCM
CH3CN
as_pmv6c12biNEW_cd3cn_1hnmr.20.fid
16
15
14
13
12
11
10
9
8
7
6
5
4
3
2
1
0
-1
-2
-3
f1 (ppm)

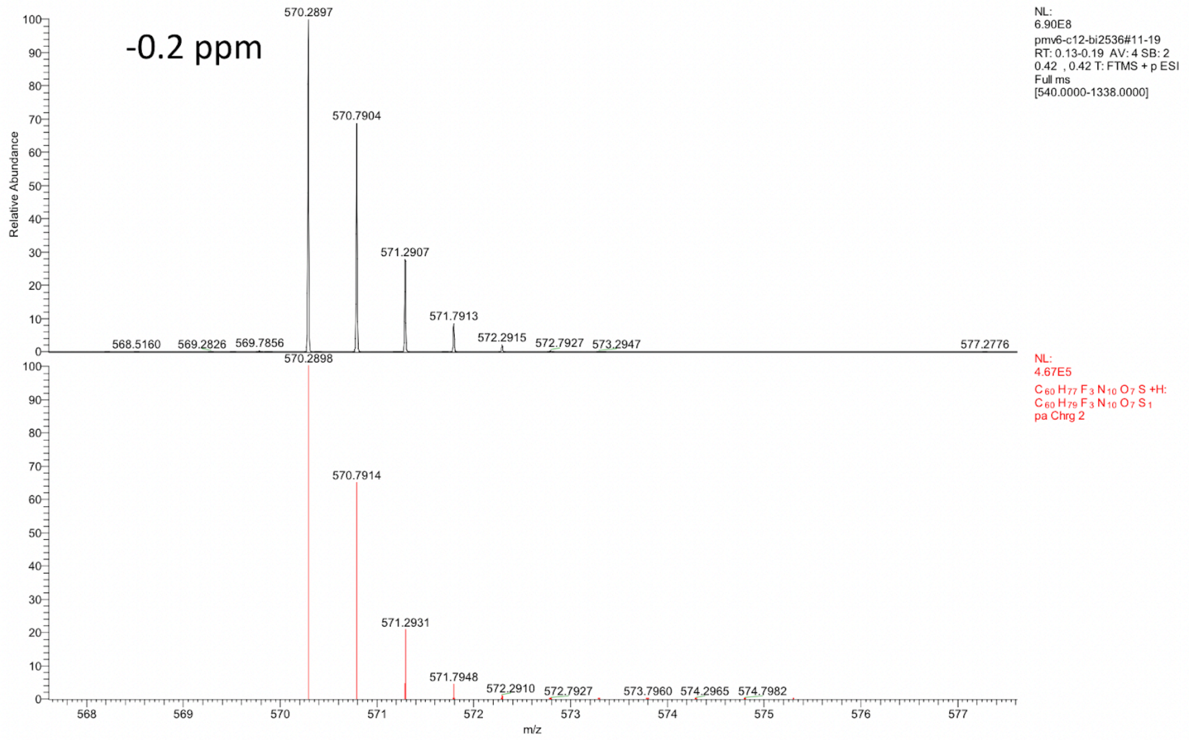
-0.2 ppm
570.2897
570.7904
571.2907
571.7913
572.2915
572.7927
573.2947
568.5160
569.2826
569.7856
577.2776
NL:
6.90E8
pmv6-c12-bi2536#11-19
RT: 0.13-0.19 AV: 4 SB: 2
0.42 , 0.42 T: FTMS + p ESI
Full ms
[540.0000-1338.0000]
Relative Abundance
570.2898
570.7914
571.2931
571.7948
572.2910
572.7927
573.7960
574.2965
574.7982
NL:
4.67E5
C60 H77 F3 N10 O7 S +H:
C60 H79 F3 N10 O7 S1
pa Chrg 2
m/z

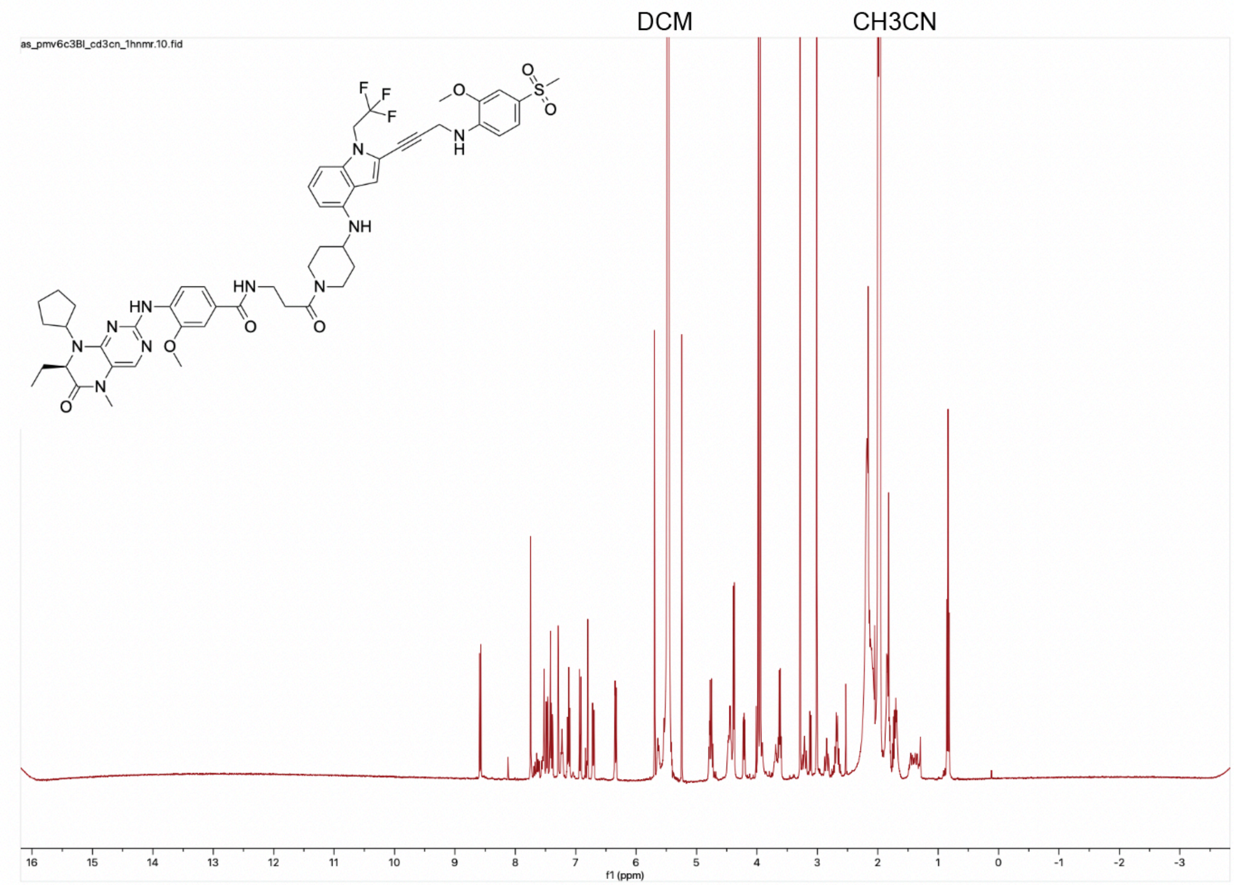
DCM
CH3CN
as_pmv6c3BI_cd3cn_1hnmr.10.fid
f1 (ppm)

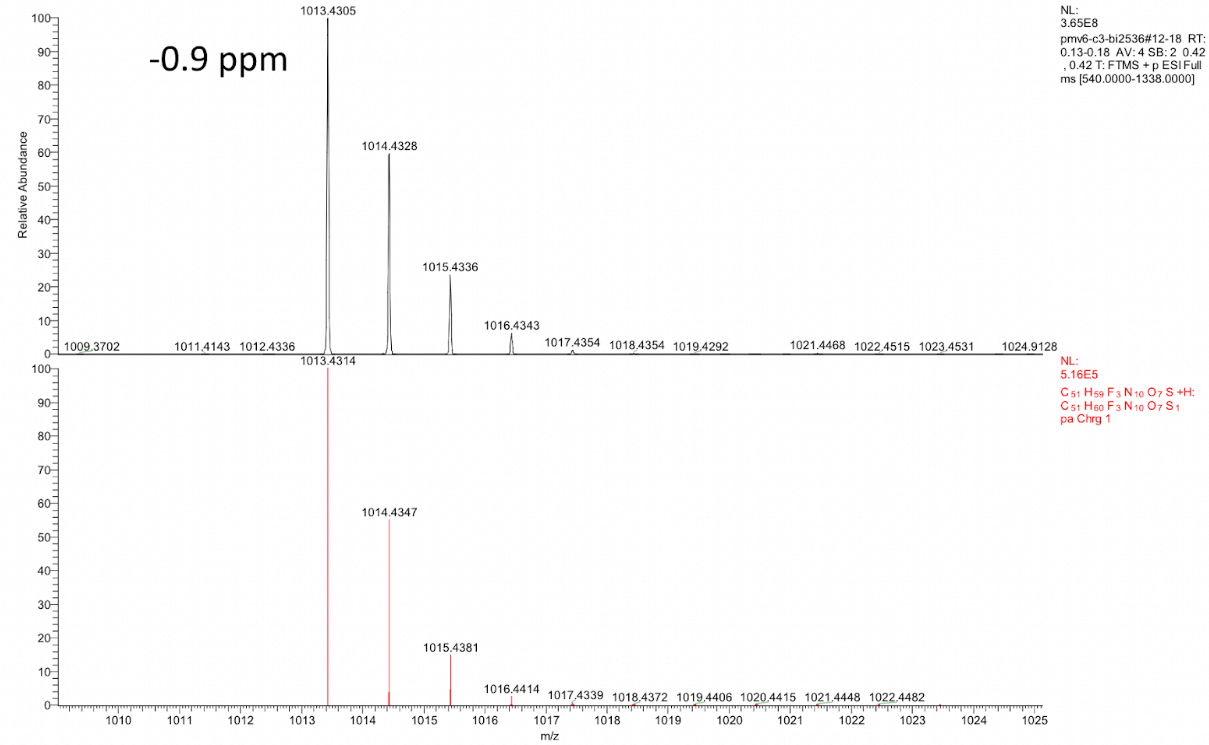
-0.9 ppm
NL:
3.65E8
pmv6-c3-bi2536#12-18 RT: 0.13-0.18 AV: 4 SB: 2 0.42 , 0.42 T: FTMS + p ESI Full ms [540.0000-1338.0000]
1013.4305
1014.4328
1015.4336
1016.4343
1017.4354
1018.4354
1019.4292
1021.4468
1022.4515
1023.4531
1024.9128
1009.3702
1011.4143
1012.4336
NL:
5.16E5
C51 H59 F3 N10 O7 S +H:
C51 H60 F3 N10 O7 S1
pa Chrg 1
1013.4314
1014.4347
1015.4381
1016.4414
1017.4339
1018.4372
1019.4406
1020.4415
1021.4448
1022.4482
Relative Abundance
m/z

PMV6-PEG4-Adavosertib.

26

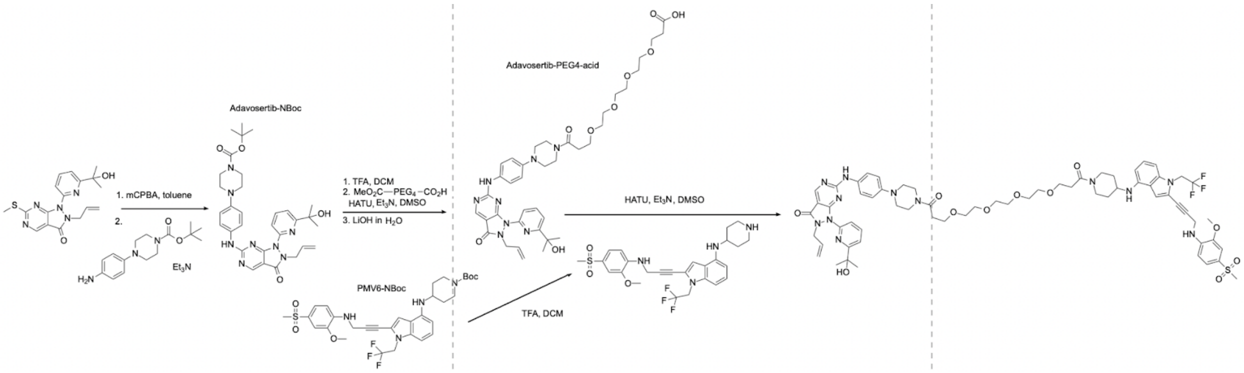

23 24 25 20 27 7

1-(4-(4-((2-allyl-1-(6-(2-hydroxypropan-2-yl)pyridin-2-yl)-3-oxo-2,3-dihydro-1H-pyrazolo[3,4-d]pyrimidin-6-yl)amino)phenyl)piperazin-1-yl)-16-(4-((2-(3-((2-methoxy-4-(methylsulfonyl)phenyl)amino)prop-1-yn-1-yl)-1-(2,2,2-trifluoroethyl)-1H-indol-4-yl)amino)piperidin-1-yl)-4,7,10,13-tetraoxahexadecane-1,16-dione (PMV6-PEG4-Adavosertib, PMV6-PEG4-Ada). 2-allyl-1-(6-(2-hydroxypropan-2-yl)pyridin-2-yl)-6-(methylthio)-1,2-dihydro-3H-pyrazolo[3,4-d]pyrimidin-3-one (50 mg, 0.140 mmol, AaronChem) was dissolved in 4 mL of toluene and 3-chloroperbenzoic acid (32 mg, 0.185 mmol) was added and stirred for 1 hour. 1-Boc-4-(4-aminophenyl)piperazine (46.6 mg, 0.168 mmol, Combi-Blocks) and triethylamine (100 μL, 0.718 mmol) were added to the solution and left to stir overnight. The reaction was purified by reverse-phase HPLC (acetonitrile:water gradient up to 90%) to yield adavosertib-NBoc as a green-brown solid (38 mg, 0.0648 mmol, 46.3% yield). Some of the resulting solid (30 mg, 0.0512 mmol) was dissolved in DCM (1 mL), and trifluoroacetic acid was added to the solution (1.5 mL). After stirring for 1 hour, solvent and reactants were removed under reduced pressure. The resulting residue was dissolved in DMSO (2 mL); HATU (40 mg, 0.105 mmol), 3-oxo-2,6,9,12,15-pentaoxaoctadecan-18-oic acid (acid-PEG4-mono-methyl ester, 50 μL, 0.162 mmol), and triethylamine (50 μL, 0.359 mmol) were added to the solution and the mixture was stirred for 30 minutes. LiOH (2M solution in H_2O, 2 mL) was added to the solution and left to stir for 1 hour. The reaction was purified by reverse-phase HPLC (acetonitrile:water gradient up to 90%) to yield adavosertib-PEG4-acid as a light yellow solid (25 mg, 0.0328 mmol, 64.0%). *tert*-butyl-4-((2-(3-((2-methoxy-4-(methylsulfonyl)phenyl)amino)prop-1-yn-1-yl)-1-(2,2,2-trifluoroethyl)-1H-indol-4-yl)amino)piperidine-1-carboxylate (PMV6-NBoc, 15.1 mg, 0.0238 mmol) was dissolved in DCM (1 mL), and trifluoroacetic acid (2 mL) was added to the solution. After stirring for 1 hour, solvents were removed under reduced pressure. The residue was redissolved in DMSO (1 mL) and adavosertib-PEG4-acid (25 mg, 0.0328 mmol), HATU (20 mg, 0.0526 mmol), and triethylamine (40 μL, 0.287 mmol) were added to the solution. After completion (30 minutes), the reaction was purified by reverse-phase HPLC (acetonitrile:water gradient up to 90%) to yield PMV6-PEG4-Adavosertib as a yellow solid (15.3 mg, 0.0120 mmol, 50.4% yield). ^{1}H NMR (400 MHz, DMSO-d6) δ 8.83 (s, 1H), 8.04 (t, J = 7.9 Hz, 1H), 7.76 (d, J = 8.1 Hz, 1H), 7.61 (t, J = 6.8 Hz, 3H), 7.40 (dd, J = 8.4, 1.9 Hz, 1H), 7.26 (d, J = 2.0 Hz, 1H), 7.10 – 6.86 (m, 4H), 6.71 (d, J = 8.3 Hz, 1H), 6.48 (t, J = 6.3 Hz, 1H), 6.23 (d, J = 7.8 Hz, 1H), 5.67 (ddt, J = 16.5, 10.2, 6.0 Hz, 1H), 5.51 (d, J = 7.9 Hz, 1H), 5.32 (s, 1H), 5.03 - 4.79 (m, 3H), 4.70 (d, J = 6.0 Hz, 1H), 4.35 (q, J = 5.8 Hz, 6H), 3.90 (s, 2H), 3.69 – 3.57 (m, 6H), 3.52 – 3.38 (m, 13H), 3.20 – 3.02 (m, 4H), 2.76 (t, J = 11.8 Hz, 1H), 2.60 (dt, J = 17.2, 6.9 Hz, 4H), 2.04 – 1.90 (m, 2H), 1.48 (s, 4H), 1.41 (d, J = 6.4 Hz, 4H), 1.36 – 1.14 (m, 1H), 1.30 (d, J = 11.5 Hz, 1H), 1.07 (t, J = 7.0 Hz, 7H). ^{13}C NMR (101 MHz, DMSO-d6) δ 169.30, 168.97, 168.06, 161.62, 161.46, 160.95, 156.45, 147.47 (d, *J* = 15.8 Hz), 146.58, 141.83, 140.98, 139.23, 138.29, 132.25 (d, *J* = 80.3 Hz), 129.05 – 124.63 (m), 122.57 (d, *J* =

164.8 Hz), 118.63 (d, J = 8.3 Hz), 118.46, 116.75, 116.58, 116.15, 109.09, 108.21, 107.60, 100.04, 99.08, 93.60, 73.75, 72.78, 71.51 – 68.75 (m), 67.32 (d, J = 8.0 Hz), 56.36 (d, J = 29.1 Hz), 51.20 – 48.17 (m), 47.05, 46.20 – 42.94 (m), 41.32, 39.16, 33.27 (d, J = 5.2 Hz), 32.93, 32.66, 31.88, 31.34, 30.87, 28.11 (d, J = 7.4 Hz), 18.97. HRMS (top: observed, bottom: theoretical isotope pattern; M+H, $C_{64}H_{77}F_3N_{12}O_{11}S$+2H] m/z theoretical 640.2827, found 640.2825.

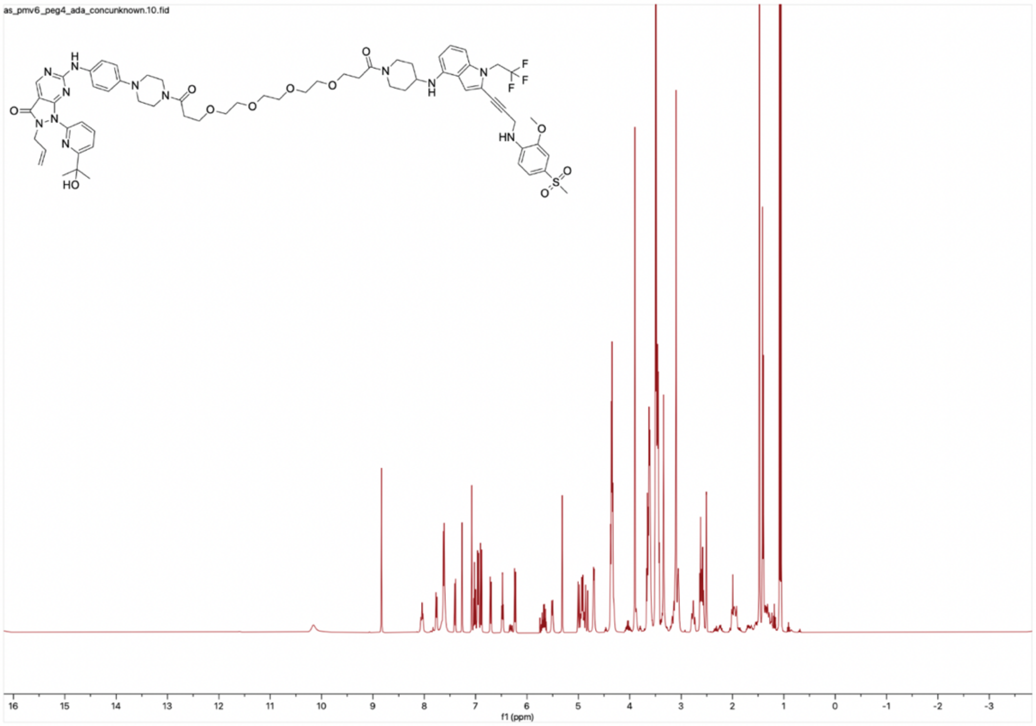

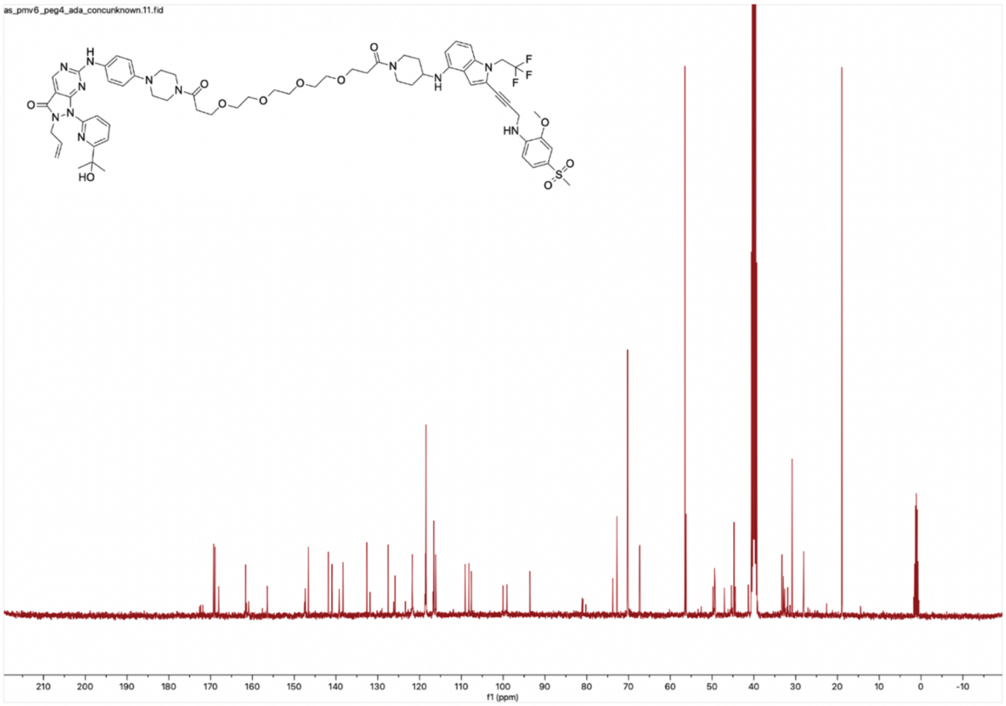

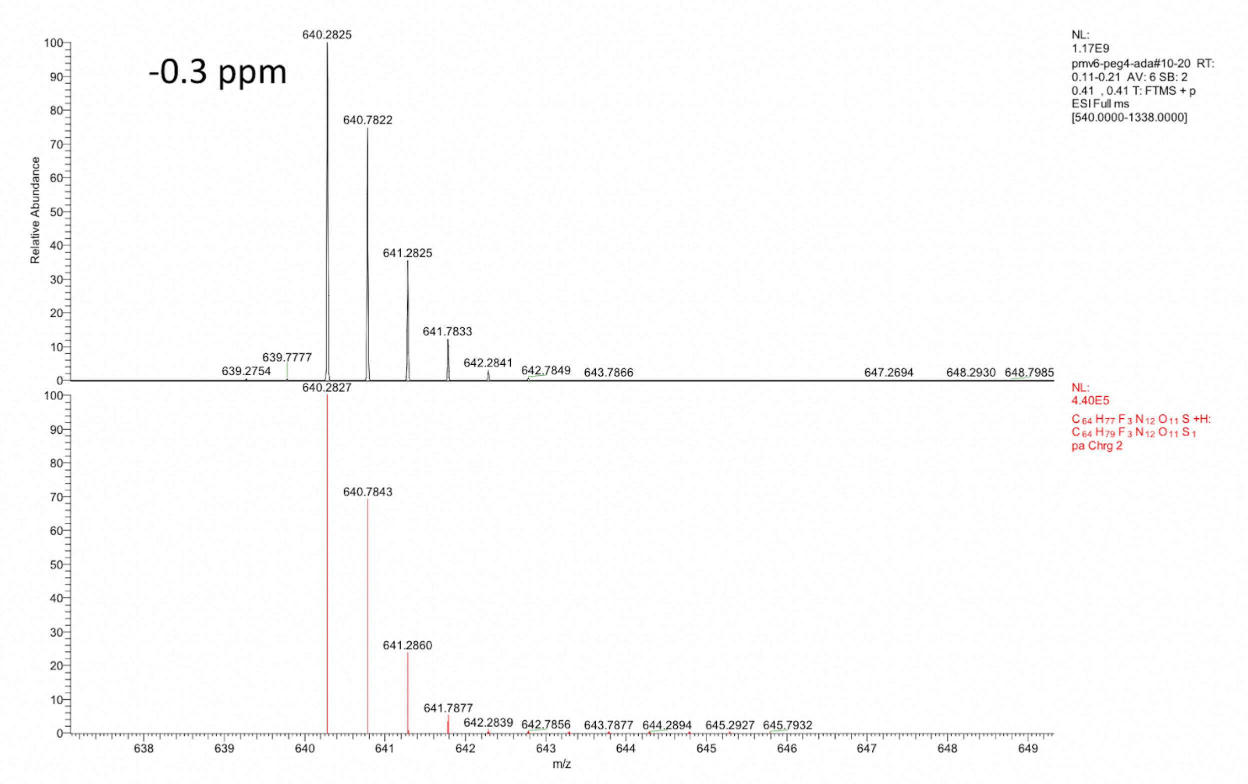

BI2536-C8-AP1867.

28

6

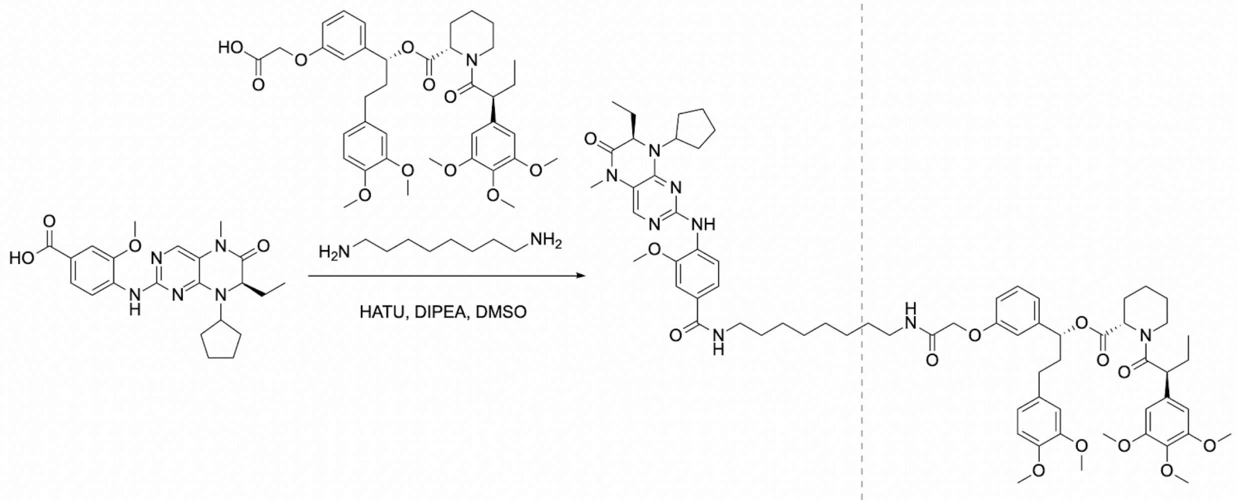

21

(R)-1-(3-(2-((8-(4-(((R)-8-cyclopentyl-7-ethyl-5-methyl-6-oxo-5,6,7,8-tetrahydropteridin-2-yl)amino)-3-methoxybenzamido)octyl)amino)-2-oxoethoxy)phenyl)-3-(3,4-dimethoxyphenyl)propyl-(S)-1-((S)-2-(3,4,5-trimethoxyphenyl)butanoyl)piperidine-2-carboxylate (BI2536-C8-AP1867). To a solution of (R)-4-(8-cyclopentyl-7-ethyl-5-methyl-6-oxo-5,6,7,8-tetrahydropteridin-2-ylamino)-3-methoxybenzoic acid (4 mg, 0.00941 mmol, 1ClickChemistry) in DMSO (1 mL), meta-AP1867 (6.5 mg,

0.00937 mmol, MedChemExpress), 1,8-diaminooctane (1.4 µL, 0.00952 mmol), HATU (8 mg, 0.0205 mmol), and DIPEA (20 µL, 0.115 mmol) were added to the solution. After completion (30 minutes), the reaction was purified by reverse-phase HPLC (acetonitrile:water gradient up to 90%) to yield BI2536-C8-AP1867 as an off-white solid (3.2 mg, 0.00261 mmol, 27.7% yield). A stock solution was created in DMSO-d6, and a portion of the stock solution was set aside for ^{1}H NMR characterization. Due to mixing with residual DCM in the NMR tube from cleaning, the portion of the stock that underwent NMR was discarded. ^{1}H NMR (400 MHz, DMSO-d6) spectrum is shown below. HRMS (top: observed, bottom: theoretical isotope pattern; M+H, $C_{68}H_{90}N_{8}O_{13}$+H] m/z theoretical 1227.6700, found 1227.6691.

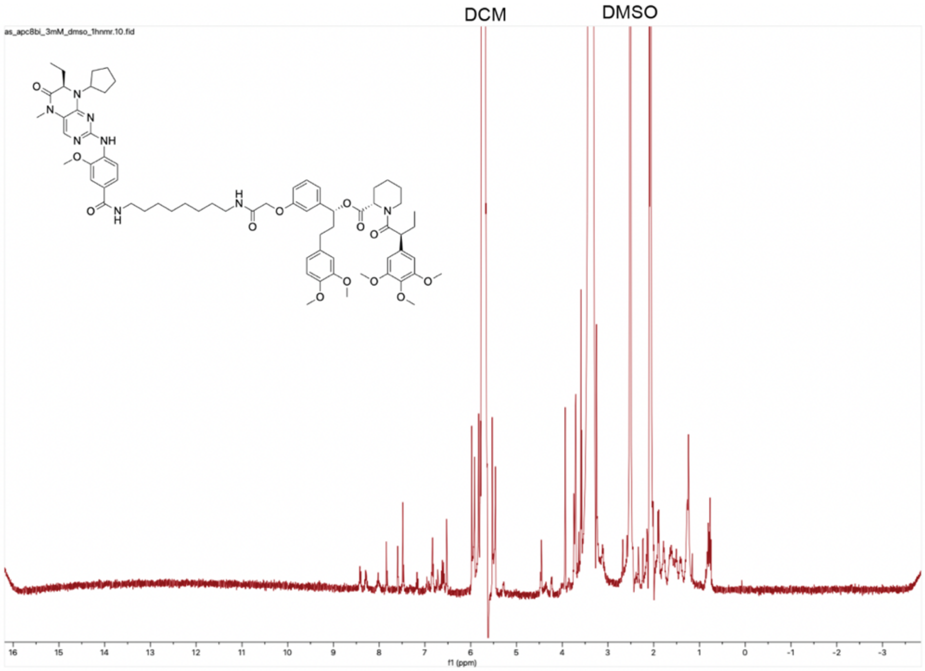

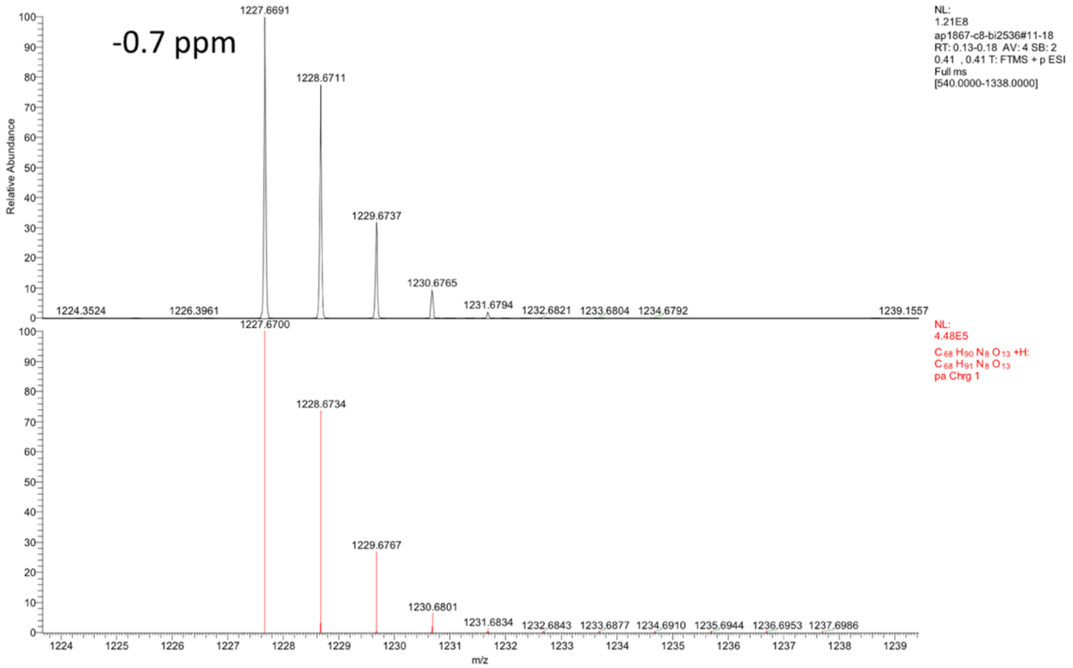

KG5-PEG4-BI2536.

21 **32**

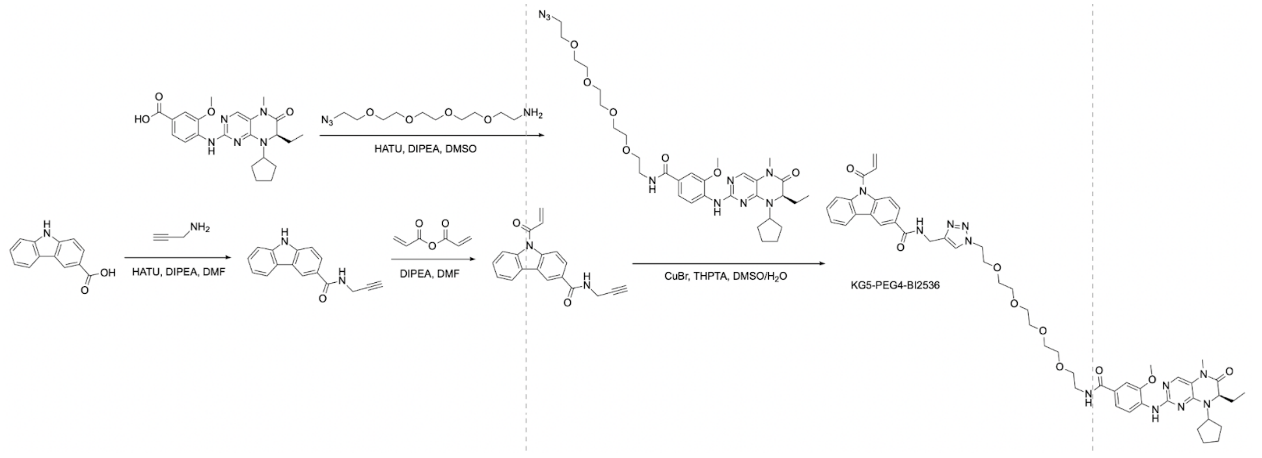

29 **30** **31** **3**

(R)-9-acryloyl-N-((1-(1-(4-((8-cyclopentyl-7-ethyl-5-methyl-6-oxo-5,6,7,8-tetrahydropteridin-2-yl)amino)-3-methoxyphenyl)-1-oxo-5,8,11,14-tetraoxa-2-azahexadecan-16-yl)-1H-1,2,3-triazol-4-yl)methyl)-9H-carbazole-3-carboxamide (KG5-PEG4-BI2536, KG5-BI2536). To a solution of 9H-carbazole-3-carboxylic acid (25 mg, 0.118 mmol, 1ClickChemistry) in DMF (1 mL), propargylamine (20 µL, 0.313 mmol), HATU (50 mg, 0.128 mmol), and DIPEA (50 µL, 0.288 mmol) were added to the solution. After completion (30 minutes), the reaction was purified by reverse-phase HPLC (acetonitrile:water gradient up to 90%) to yield N-(prop-2-yn-1-yl)-9H-carbazole-3-carboxamide as a white solid (20 mg, 0.0806 mmol, 68.3%). The resulting solid was dissolved in DMF (3 mL), and acrylic anhydride (60 µL, 0.521 mmol) and DIPEA (120 µL, 0.691 mmol) were added to the solution to react overnight. After completion, the reaction was purified by reverse-phase HPLC (acetonitrile:water gradient up to 90%) to yield 9-acryloyl-N-(prop-2-yn-1-yl)-9H-carbazole-3-carboxamide as a off-white solid (8.1 mg, 0.0268 mmol, 33.2% yield). To a solution of (R)-4-(8-cyclopentyl-7-ethyl-5-methyl-6-oxo-5,6,7,8-tetrahydropteridin-2-ylamino)-3-methoxybenzoic acid (7 mg, 0.0165 mmol, 1ClickChemistry) in DMSO (1 mL), N_3-PEG4-NH_2 (10 µL, 0.0382 mmol), HATU (8 mg, 0.0205 mmol), and DIPEA (20 µL, 0.115 mmol) were added to the solution. After completion (30 minutes), the reaction was purified by reverse-phase HPLC (acetonitrile:water gradient up to 90%) to yield (R)-N-(14-azido-3,6,9,12-tetraoxatetradecyl)-4-((8-cyclopentyl-7-ethyl-5-methyl-6-oxo-5,6,7,8-tetrahydropteridin-2-yl)amino)-3-methoxybenzamide as a off-white solid (5.5 mg, 0.00822 mmol, 49.8% yield). The resulting solid was dissolved in DMSO (1 mL) and combined with 9-acryloyl-N-(prop-2-yn-1-yl)-9H-carbazole-3-carboxamide (3.5 mg, 0.0116 mmol), CuBr (2 mg, 0.0140 mmol), THPTA (6 mg, 0.0138 mmol), and water (0.3 mL). After completion (10 minutes), the reaction was purified by reverse-phase HPLC (acetonitrile:water gradient up to 90%) to yield KG5-PEG4-BI2536 as an off-white solid (3.6 mg, 0.00370 mmol, 45.1% yield). A stock solution was created in DMSO, and a portion of the stock solution was set aside for ^{1}H NMR characterization. Due to mixing with residual DCM in the NMR tube from cleaning, the portion of the stock that underwent NMR was discarded. ^{1}H NMR (400 MHz, DMSO) spectrum is shown below. HRMS (top: observed, bottom: theoretical isotope pattern; M+H, $C_{51}H_{61}N_{11}O_9$+H] m/z theoretical 972.4726, found 972.4709.

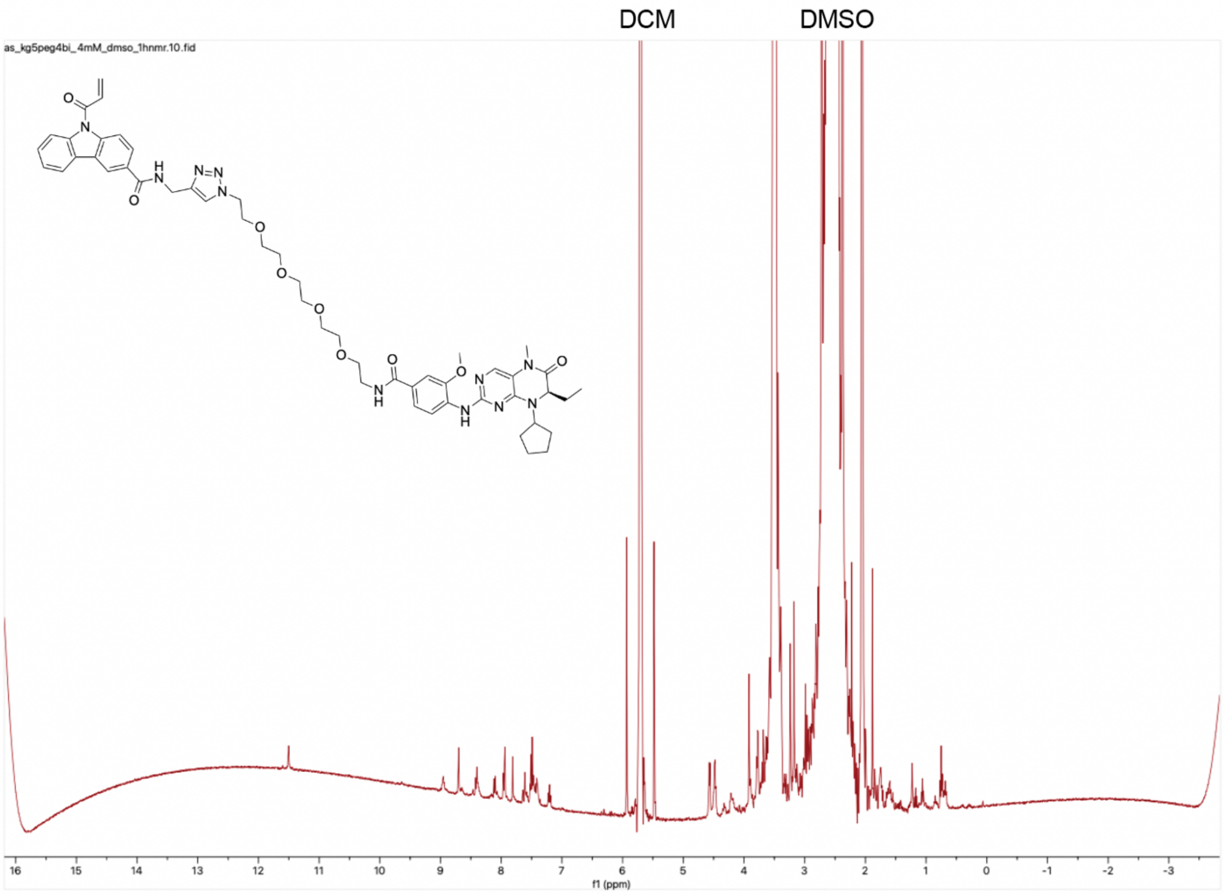
DCM
DMSO
as_kg5peg4bi_4mM_dmso_1hnmr.10.fid
f1 (ppm)
16 15 14 13 12 11 10 9 8 7 6 5 4 3 2 1 0 -1 -2 -3

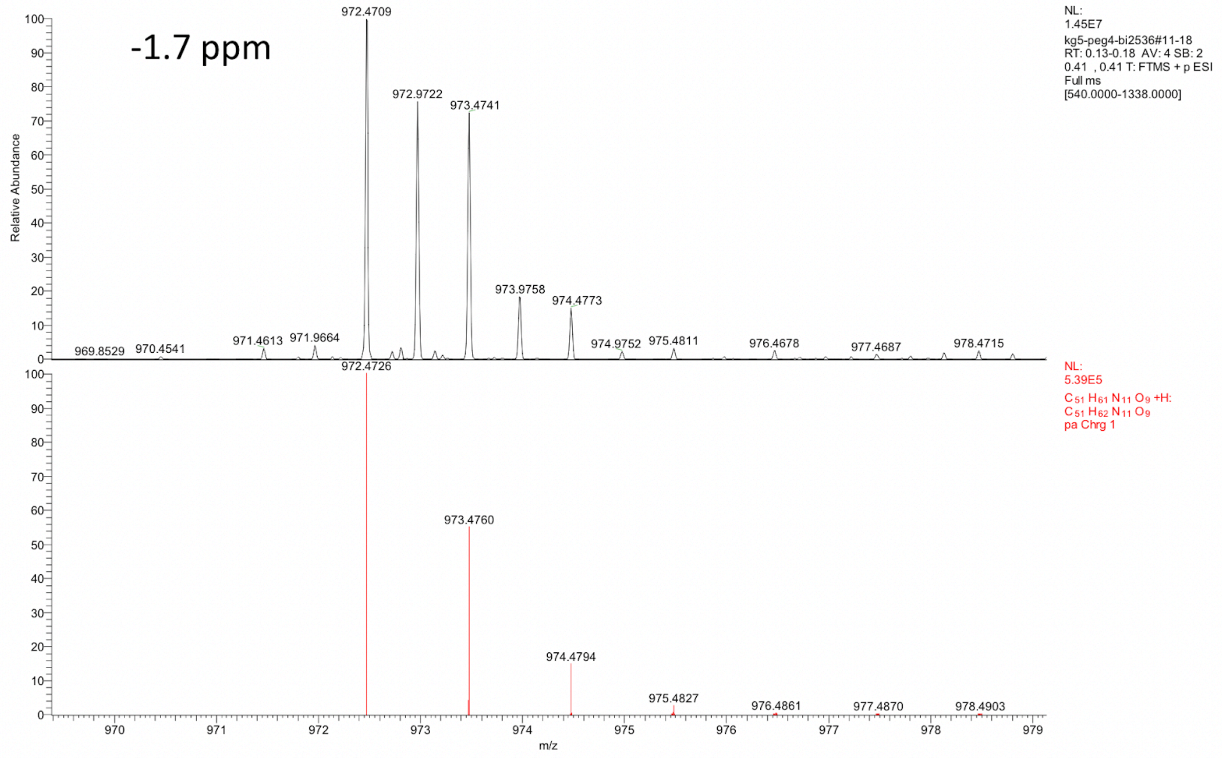
-1.7 ppm
972.4709
972.9722
973.4741
973.9758
974.4773
969.8529
970.4541
971.4613
971.9664
974.9752
975.4811
976.4678
977.4687
978.4715
NL:
1.45E7
kg5-peg4-bi2536#11-18
RT: 0.13-0.18 AV: 4 SB: 2
0.41 , 0.41 T: FTMS + p ESI
Full ms
[540.0000-1338.0000]
972.4726
973.4760
974.4794
975.4827
976.4861
977.4870
978.4903
NL:
5.39E5
C51 H61 N11 O9 +H:
C51 H62 N11 O9
pa Chrg 1
Relative Abundance
m/z

BI2536-PEG2-Halo (HLDA-131).

21 **33** **2**

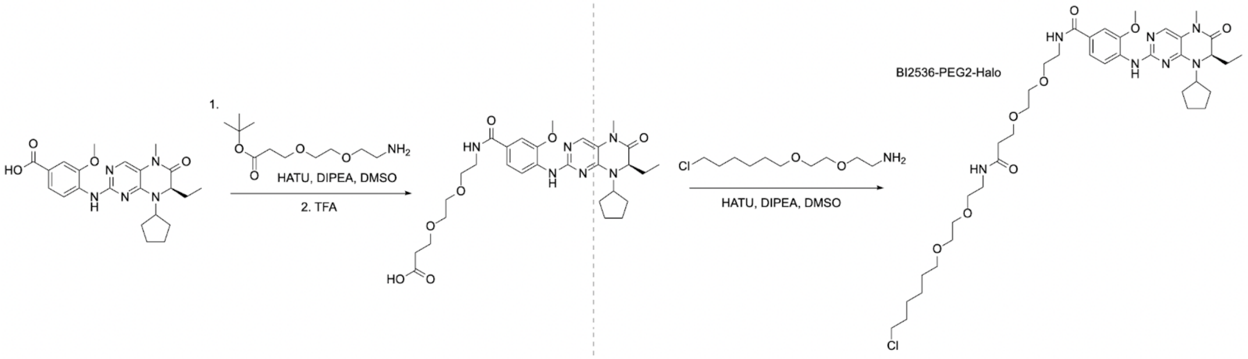

(R)-N-(22-chloro-9-oxo-3,6,13,16-tetraoxa-10-azadocosyl)-4-((8-cyclopentyl-7-ethyl-5-methyl-6-oxo-5,6,7,8-tetrahydropteridin-2-yl)amino)-3-methoxybenzamide (HLDA-131, BI2536-PEG2-Halo). To a solution of (R)-4-(8-cyclopentyl-7-ethyl-5-methyl-6-oxo-5,6,7,8-tetrahydropteridin-2-ylamino)-3-methoxybenzoic acid (10 mg, 0.0235 mmol, 1ClickChemistry) in DMSO (1 mL), NH_2-PEG2-$CH_2CH_2CO_2$tBu (20 µL, 0.0858 mmol), HATU (12 mg, 0.0315 mmol), and DIPEA (20 µL, 0.115 mmol) were added. After stirring for 30 minutes, trifluoroacetic acid (2 mL) was added and the reaction was stirred for 16 hours. After completion, the reaction was purified by reverse-phase HPLC (acetonitrile:water gradient up to 90%) to yield (R)-3-(2-(2-(4-((8-cyclopentyl-7-ethyl-5-methyl-6-oxo-5,6,7,8-tetrahydropteridin-2-yl)amino)-3-methoxybenzamido)ethoxy)ethoxy)propanoic acid (BI2536-PEG2-acid) as a yellow solid (7 mg, 0.0120 mmol, 51.1% yield). To a solution of the product in DMSO (1 mL), 2-(2-((6-chlorohexyl)oxy)ethoxy)ethan-1-amine hydrochloride (10 mg, 0.0385 mmol, MedChemExpress), HATU (8 mg, 0.0205 mmol), and DIPEA (20 µL, 0.115 mmol) were added to the solution. After completion (30 minutes), the reaction was purified by reverse-phase HPLC (acetonitrile:water gradient up to 90%) to yield HLDA-131 as a yellow solid (5.2 mg, 0.00658 mmol, 54.8% yield). ^{1}H NMR spectra matched what was previously reported[33]. HRMS (top: observed, bottom: theoretical isotope pattern; M+H, $C_{39}H_{60}ClN_7O_8$+H] m/z theoretical 790.4265, found 790.4254.

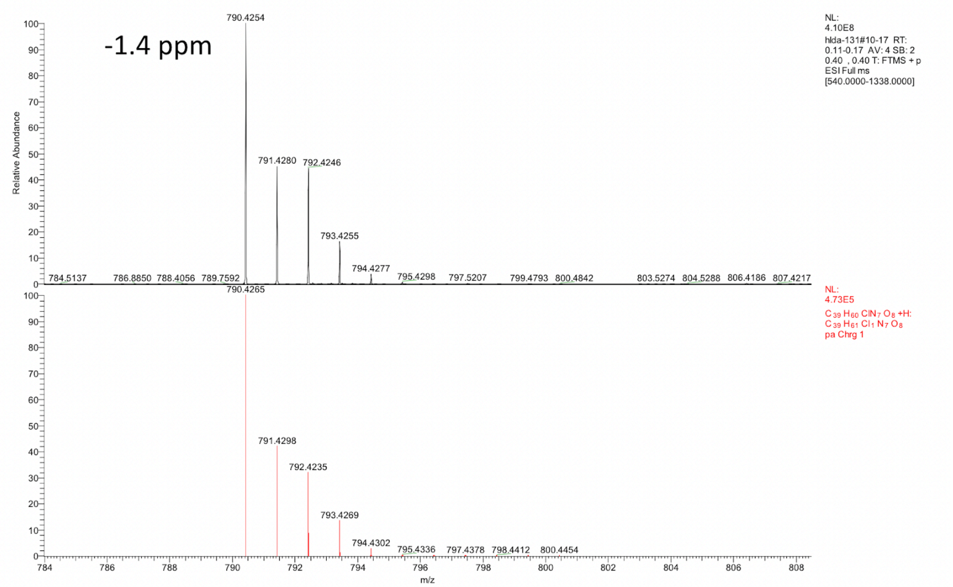

Adavosertib-PEG2-Halo.

25 **1**

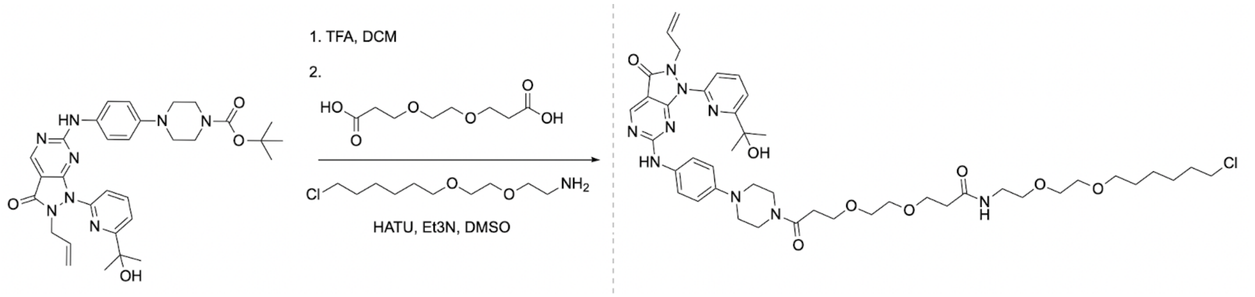

3-(2-(3-(4-(4-((2-allyl-1-(6-(2-hydroxypropan-2-yl)pyridin-2-yl)-3-oxo-2,3-dihydro-1H-pyrazolo[3,4-d]pyrimidin-6-yl)amino)phenyl)piperazin-1-yl)-3-oxopropoxy)ethoxy)-N-(2-(2-((6-chlorohexyl)oxy)ethoxy)ethyl)propanamide (Adavosertib-PEG2-Halo, Ada-PEG2-Halo). Adavosertib-NBoc (synthesis described in the PMV6-PEG4-Adavosertib section, 22.2 mg, 0.0379 mmol) was dissolved in DCM (1 mL) and TFA was added (3 mL); the mixture was left to stir for 1 hour and solvents were evaporated under reduced pressure. The residue was dissolved in DMSO (2 mL). PEG2 diacid (7.8 mg, 0.0379 mmol) and 2-(2-((6-chlorohexyl)oxy)ethoxy)ethan-1-amine hydrochloride (9.8 mg, 0.0378 mmol) were added, followed by triethylamine (75 µL, 0.539 mmol) and HATU (31.6 mg, 0.0832 mmol). After completion (30 minutes), the reaction was purified by reverse-phase HPLC (acetonitrile:water gradient up to 90%) to yield adavosertib-PEG2-Halo as a yellow solid (12.2 mg, 0.0139 mmol, 36.7% yield). ^{1}H NMR (400 MHz, DMSO-d6) δ 8.84 (s, 1H), 8.05 (t, *J* = 7.8 Hz, 1H), 7.88 (t, *J* = 5.6 Hz, 1H), 7.76 (d, *J* = 8.1 Hz, 1H), 7.65 – 7.58 (m, 1H), 7.60 (s, 1H), 6.96 (d, *J* = 8.7 Hz, 2H), 5.76 (s, 2H), 5.67 (ddt, *J* = 16.4, 10.2, 6.0 Hz, 1H), 5.32 (s, 1H), 5.04 – 4.96 (m, 1H), 4.83 (dq, *J* = 17.1, 1.5 Hz, 1H), 4.69 (d, *J* = 6.0 Hz, 2H), 3.69 – 3.55 (m, 9H), 3.48 (tp, *J* = 5.2, 2.7 Hz, 8H), 3.47 – 3.31 (m, 4H), 3.19 (q, *J* = 5.9 Hz, 2H), 3.12 (t, *J* = 5.1 Hz, 2H), 3.06 (t, *J* = 5.2 Hz, 2H), 2.63 (t, *J* = 6.6 Hz, 2H), 2.31 (t, *J* = 6.5 Hz, 2H), 1.75 – 1.63 (m, 2H), 1.50 (d, *J* = 6.8 Hz, 1H), 1.47 (s, 7H), 1.46 (s, 1H), 1.43 – 1.21 (m, 4H). ^{13}C NMR (101 MHz, DMSO-d6) δ 170.57, 169.26, 168.06, 161.60, 160.95, 156.49, 147.38, 139.27, 132.67, 131.85, 118.73, 116.75, 116.60, 72.79, 70.66, 70.08, 70.05, 69.98, 69.89, 69.58, 67.28, 55.38, 49.80, 49.32, 47.06, 45.81, 45.34, 41.32, 39.00, 36.51, 33.28, 32.48, 30.92, 29.53, 26.59, 25.39. HRMS (top: observed, bottom: theoretical isotope pattern; M+H, $C_{44}H_{62}ClN_9O_8$+H] m/z theoretical 880.4483, found 880.4473.

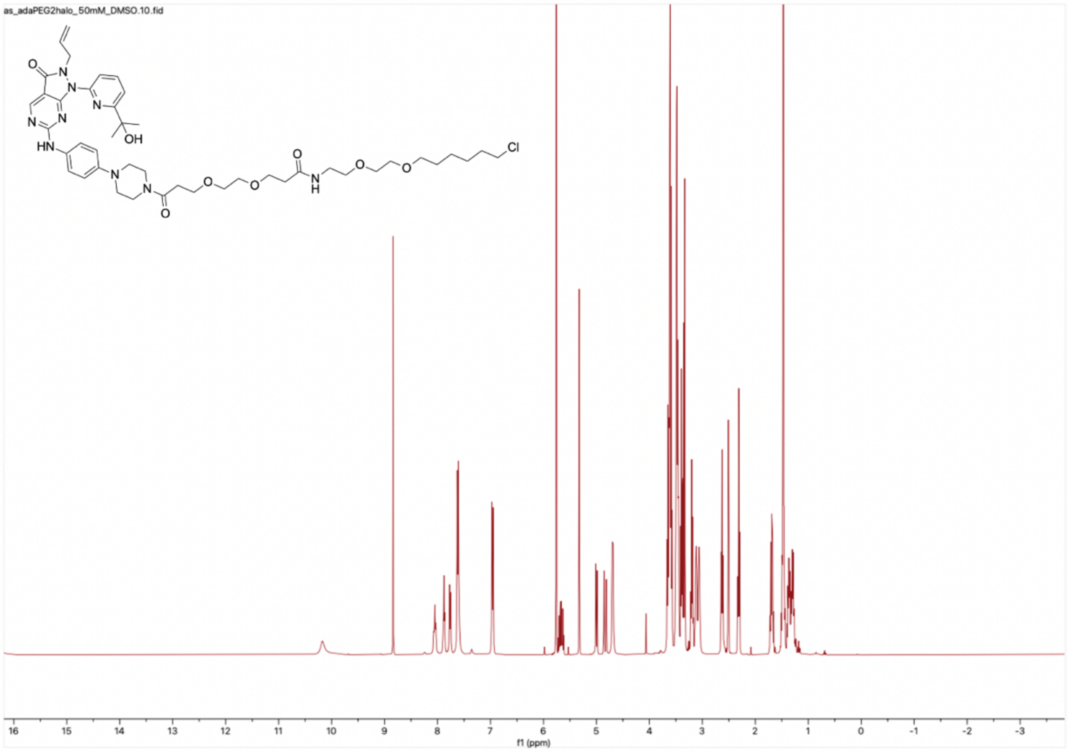

as_adaPEG2halo_50mM_DMSO.10.fid
f1 (ppm)

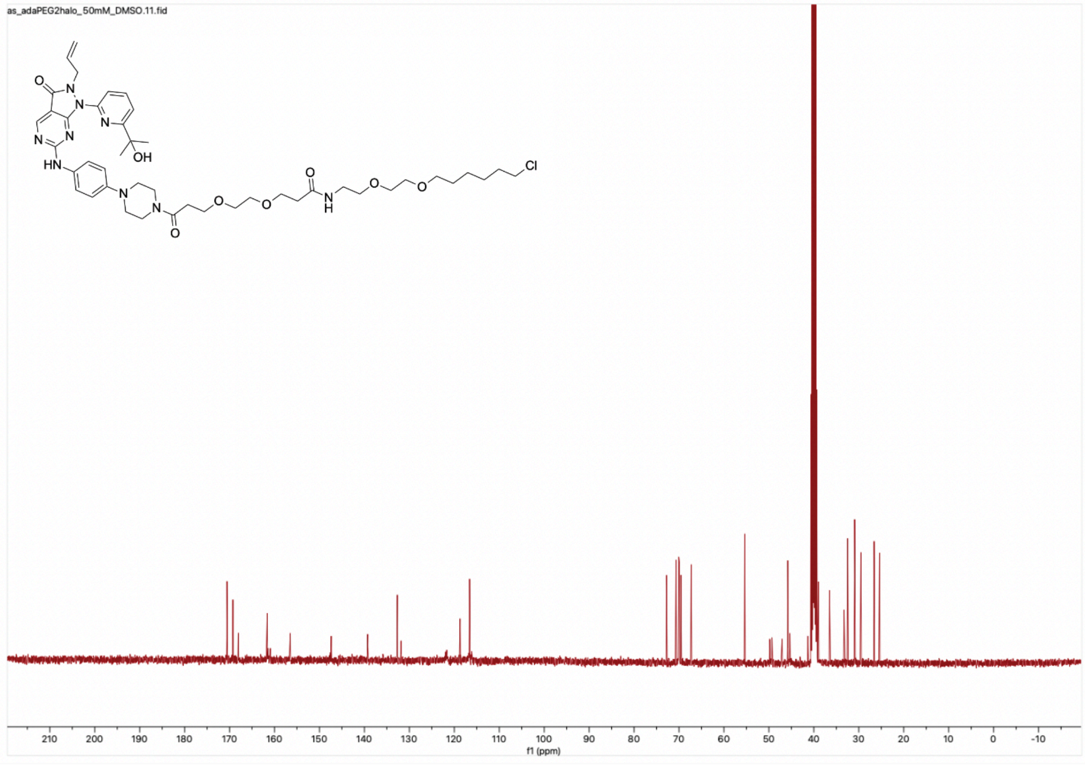

as_adaPEG2halo_50mM_DMSO.11.fid
f1 (ppm)

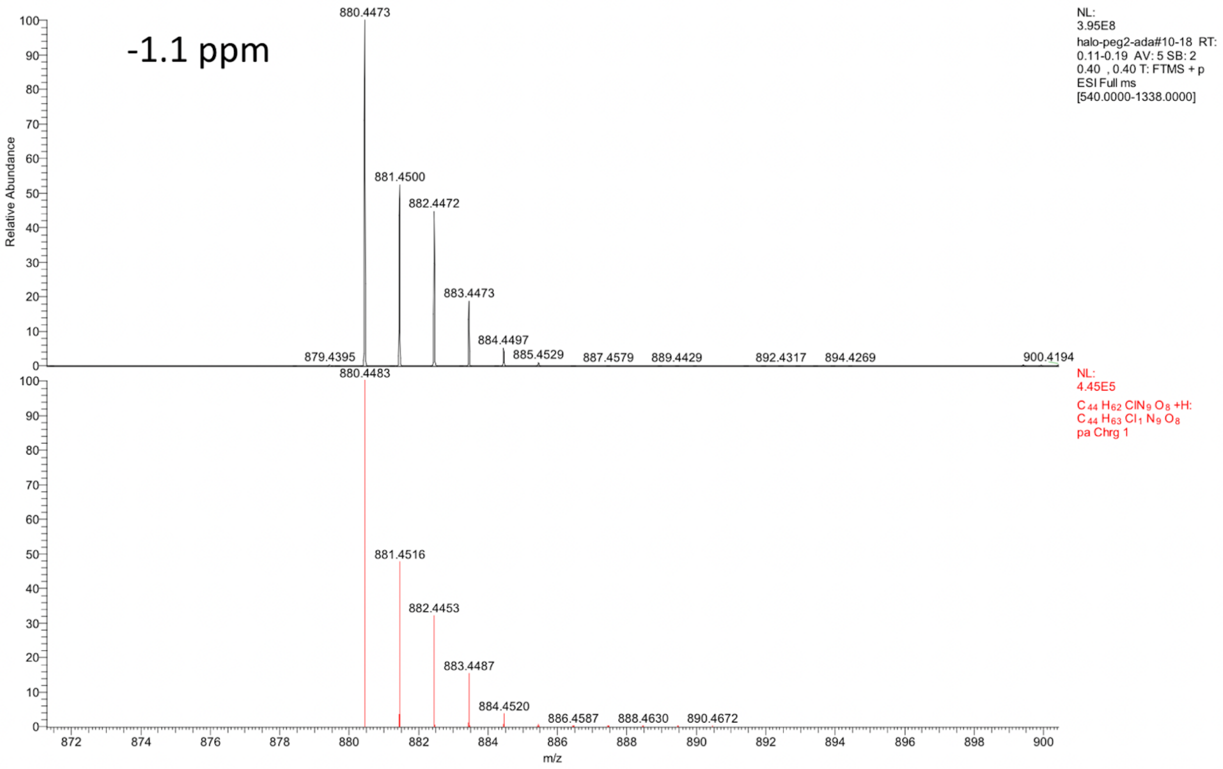

-1.1 ppm
NL:
3.95E8
halo-peg2-ada#10-18 RT:
0.11-0.19 AV: 5 SB: 2
0.40 , 0.40 T: FTMS + p
ESI Full ms
[540.0000-1338.0000]
Relative Abundance
880.4473
881.4500
882.4472
883.4473
884.4497
885.4529
887.4579
889.4429
892.4317
894.4269
900.4194
879.4395
NL:
4.45E5
C 44 H62 ClN9 O8 +H:
C 44 H63 Cl1 N9 O8
pa Chrg 1
880.4483
881.4516
882.4453
883.4487
884.4520
886.4587
888.4630
890.4672
m/z
