# Supplementary figures and images for: Mutant p53 protein accumulation is selectively targetable by proximity-inducing drugs
Source: Nat Chem Biol. 2025 Nov 3;22(5):783–92. doi: 10.1038/s41589-025-02051-7 (PMC13128454; doi:10.1038/s41589-025-02051-7)

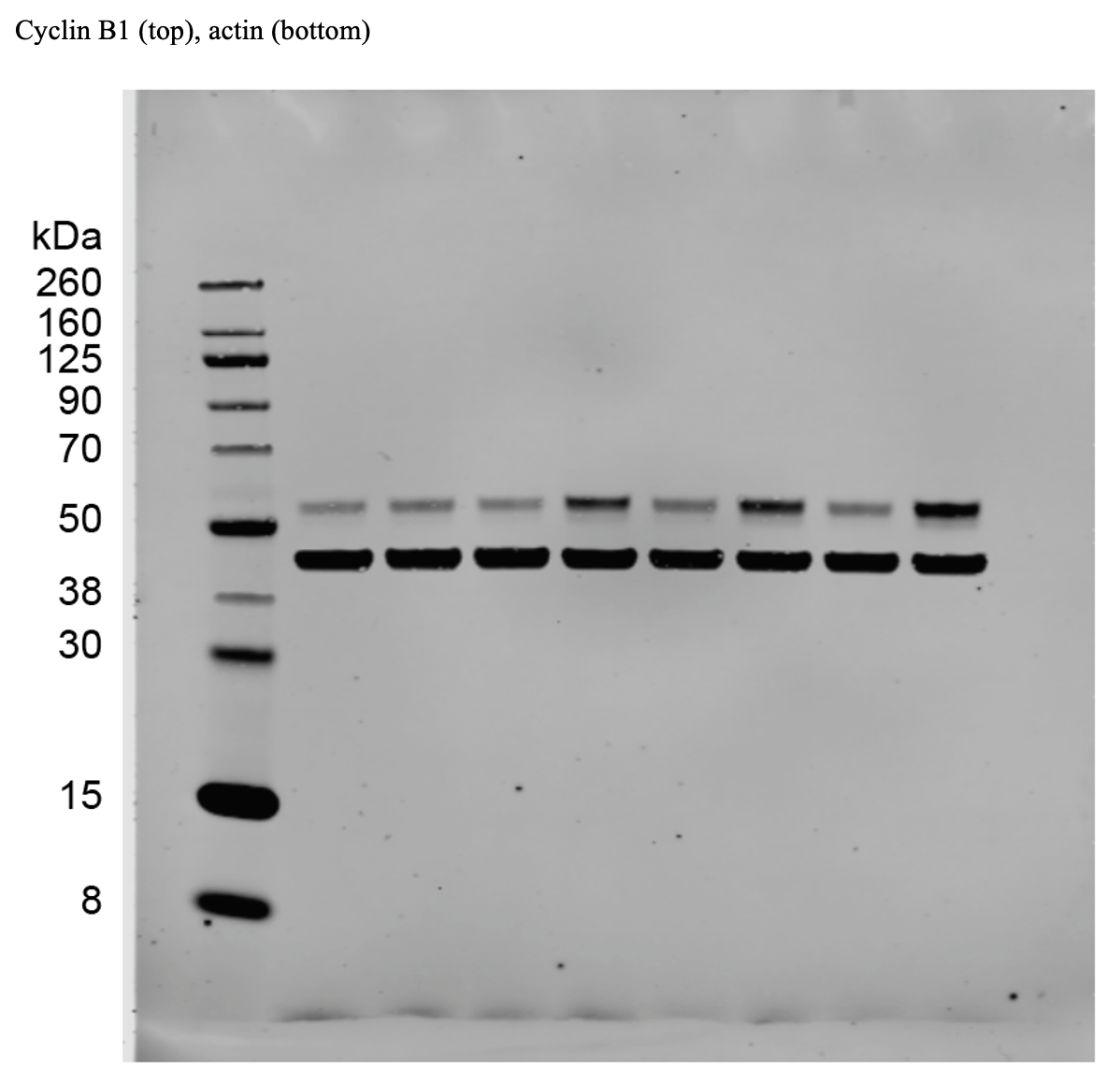

Supplement: Supplementary file 5 — Uncropped blots. [file 41589_2025_2051_MOESM5_ESM.zip › Fig3/CyclinB1_actin_blot.png]

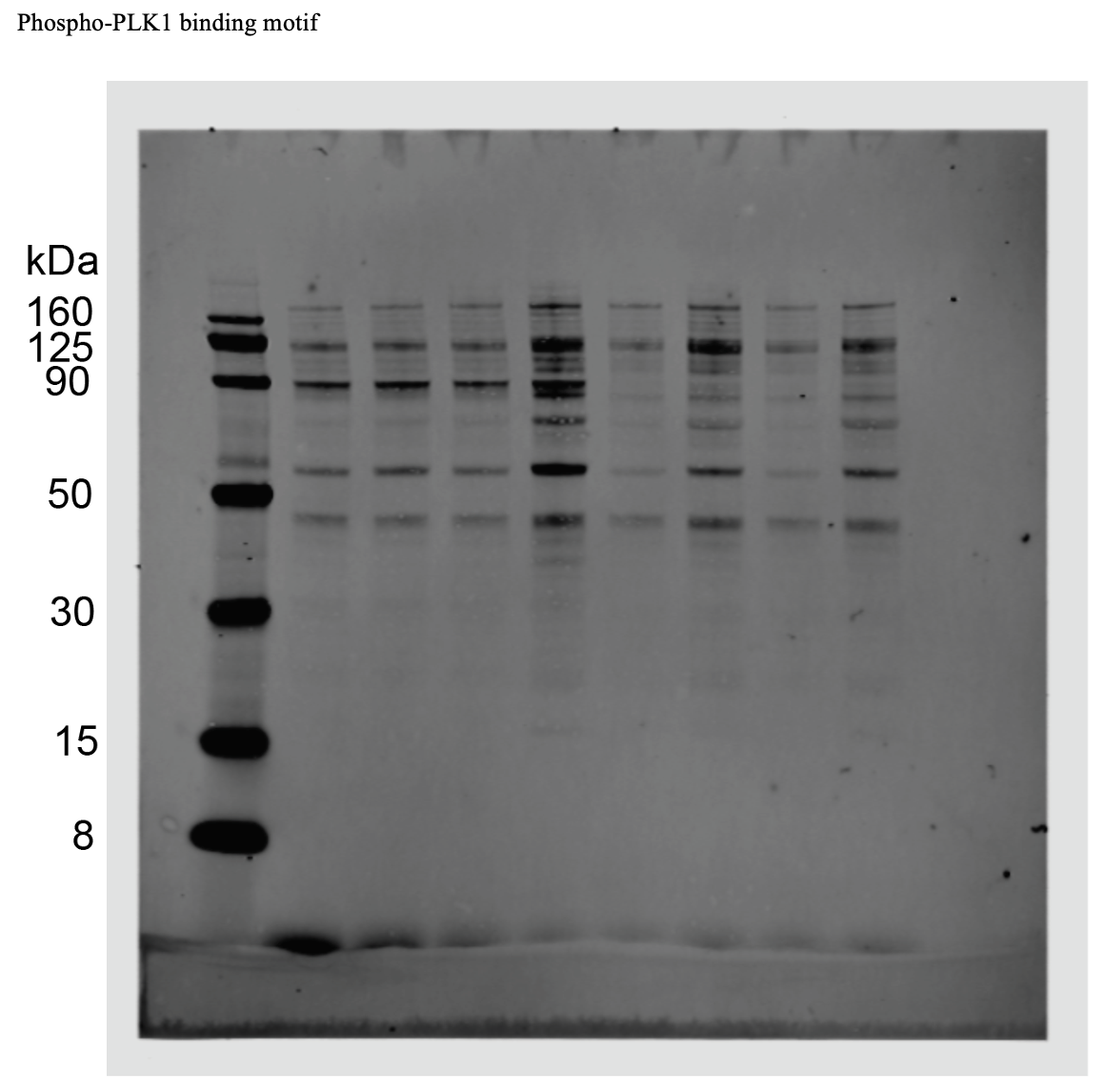

Supplement: Supplementary file 5 — Uncropped blots. [file 41589_2025_2051_MOESM5_ESM.zip › Fig3/PhosphoPLK1_blot.png]
